# Transition metal-free visible light photoredox-catalyzed remote C(sp^3^)−H borylation enabled by 1,5-hydrogen atom transfer

**DOI:** 10.1038/s42004-023-00960-z

**Published:** 2023-07-24

**Authors:** Beiqi Sun, Wenke Li, Qianyi Liu, Gaoge Zhang, Fanyang Mo

**Affiliations:** 1 School of Materials Science and Engineering, Peking University, Yiheyuan Road, Beijing, 100871 China; 2 College of Engineering, Peking University, Yiheyuan Road, Beijing, 100871 China

**Keywords:** Homogeneous catalysis, Photocatalysis, Synthetic chemistry methodology

## Abstract

The borylation of unreactive carbon-hydrogen bonds is a valuable method for transforming feedstock chemicals into versatile building blocks. Here, we describe a transition metal-free method for the photoredox-catalyzed borylation of unactivated C(sp^3^)−H bond, initiated by 1,5-hydrogen atom transfer (HAT). The remote borylation was directed by 1,5-HAT of the amidyl radical, which was generated by photocatalytic reduction of hydroxamic acid derivatives. The method accommodates substrates with primary, secondary and tertiary C(sp^3^)−H bonds, yielding moderate to good product yields (up to 92%) with tolerance for various functional groups. Mechanistic studies, including radical clock experiments and DFT calculations, provided detailed insight into the 1,5-HAT borylation process.

## Abstract (web summary)

Alkyl boronic esters are commonly used building blocks in organic synthesis, causing efficient C-H borylation methods to be in high demand. Here, the authors develop a method for the photoredox-catalyzed borylation of unreactive C(sp^3^)-H bonds, mediated by amidyl radicals to achieve C-H borylation at the *λ* position of carbonyl compounds.

## Introduction

**Fig. 1:**
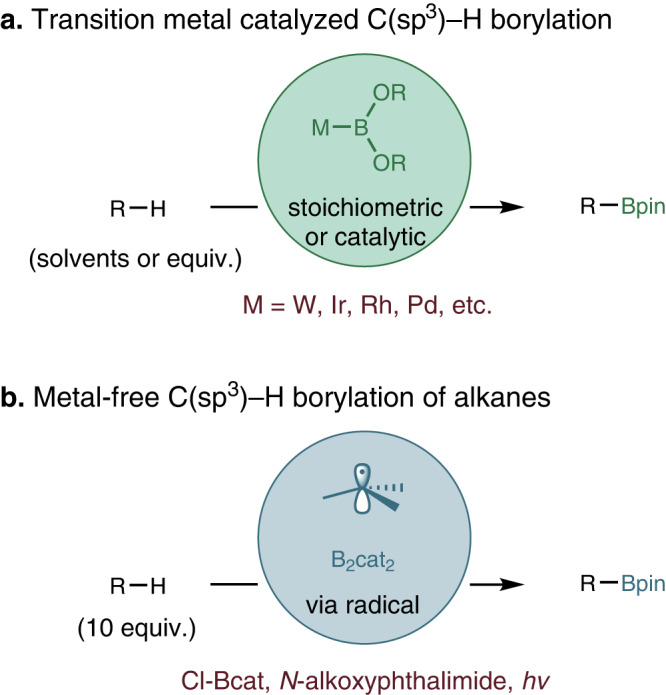
Unactivated C(sp^3^)−H bonds borylation. Previously reported: **a** Transition metal catalyzed C(sp^3^)−H borylation. **b** The metal-free photoinduced borylation of alkanes by Aggarwal group.

Alkyl boronic esters are highly versatile building blocks in organic synthesis because C−B bonds can be easily transformed into various useful functional groups^1^. C−H borylation is desirable and powerful because it directly transforms common C−H bonds into synthetically valuable C−B bonds. Borylation of nonactivated C(sp^3^)−H is one of the most challenging subjects in the area. In the past decades, a series of methods have been established^2–4^. and most of them require transition-metals as catalysts, including W^5^, Rh^6^, Pd^7–10^, Ir^11–24^ (Fig. 1a), and the reaction conditions could be very harsh (temperature 100–150 °C). In 2020, the Aggarwal group reported the metal-free photoinduced borylation of alkanes (Fig. 1b)^25^. However, due to the low reactivity of alkanes, the reaction requires high equivalency of alkane substrates (10 equiv.), and the yields based on diboron are relatively low (lower than 50% in most cases). Given that boronic esters are important in organic synthesis and medicinal chemistry, it is of great significance to develop substrate equivalent and efficient C−H borylation methods.

**Fig. 2:**
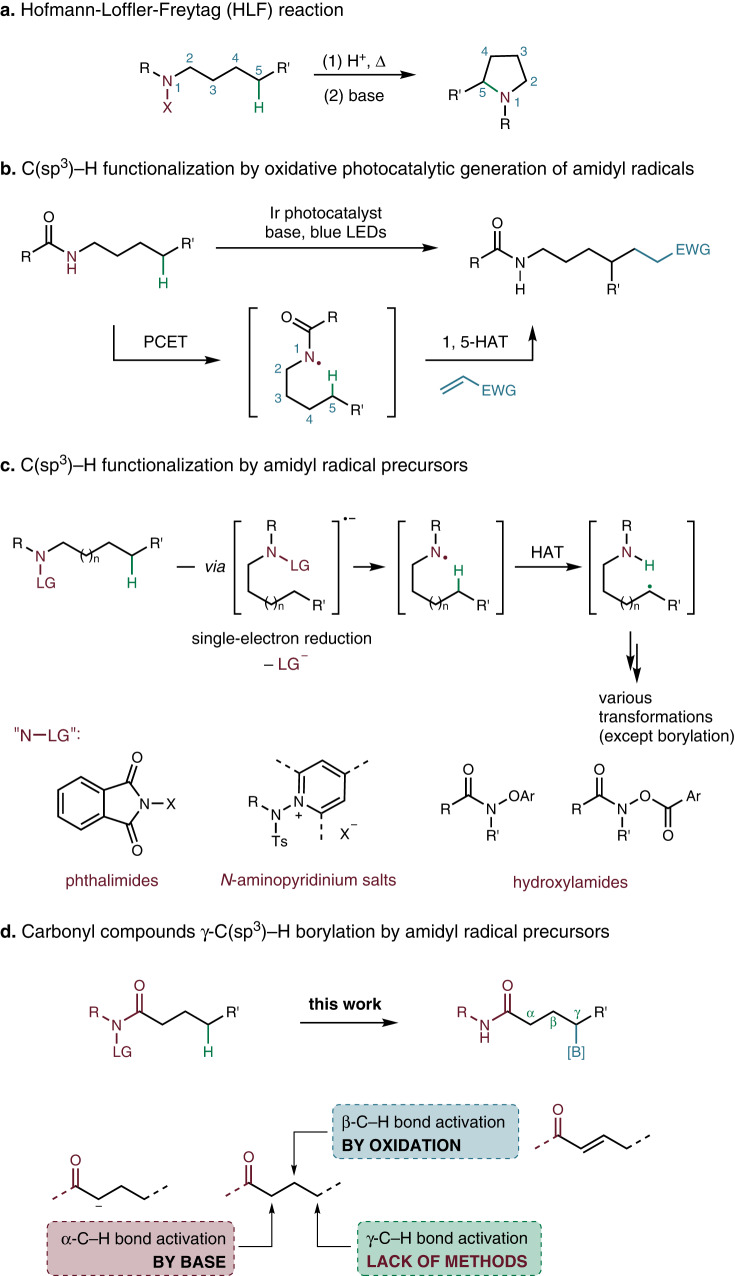
HLF type remote C−H functionalization reactions. **a** HLF reaction. **b** C(sp^3^)−H functionalization via amidyl radical by Knowles and Rovis group independently. **c** C(sp^3^)−H functionalization by amidyl radical precursors. “N-LG" showed some of the precursors. **d** This work: carbonyl compounds *γ*-C(sp^3^)−H borylation.

Hydrogen atom transfer (HAT) is a general strategy in organic synthesis, and it represents an attractive approach toward remote C−H functionalization^26–28^. The earliest example of remote C−H functionalization by means of HAT was Hofmann–Löffler–Freytag (HLF) reaction (Fig. 2a)^29,30^, the key step is the 1,5-hydrogen atom abstraction via nitrogen-centered radical, where the bond-dissociation energy (BDE) of N−H bond is higher than the C−H bond. In past decades, the nitrogen-centered radical-based HLF type reaction has shown its advances in regioselective functionalization of inert C−H bonds^31,32^, by trapping the carbon-centered radicals towards the formations of C−C^33,34^, C−O^35,36^, C−N^37–40^, and C−X (X=halogen)^41–44^ bonds. Particularly, amidyl radical is widely explored nitrogen-centered radical in above mentioned processes. In 2016, Knowles^45^ and Rovis^46^ independently developed oxidative photocatalytic generation of amidyl radicals by direct N−H bond cleavage, after 1,5-HAT process, followed by the Michael addition of the translocated carbon-centered radical, affording the remote functionalized amides (Fig. 2b). Another way to generate amidyl radical is using activated precursors^47^, for example, single-electron photoreduction of *N*-acyloxy(halo)phthalimides^48,49^, *N*-aminopyridinium salts^50–53^, and hydroxylamides^40,47,54–58^ give the corresponding amidyl radicals upon heterolytic cleavage of the respective leaving groups (Fig. 2c). Despite these advances, trapping the carbon-centered radical with diboron compounds to build C−B bonds, is still challenging and remains unexplored.

In our previous studies, we focused on developing transition metal-free radical borylation reactions^59–63^. We therefore turned our interest towards the use of amidyl radicals for remote C−H bond (*γ*-position of a carbonyl compound) borylation reaction (Fig. 2d). With a properly designed amidyl radical precursor that facilitates an intramolecular 1,5-HAT process, site-selective borylation of *γ*-C−H bond could be achieved. It is known that *α*-C−H bond and *β*-C−H bond could be simply activated^64^. For the direct *γ*-C−H functionalization, only a few of examples have been reported to date^65–68^. Thus, our research offers another significant aspect worthy of consideration. Here we present a method that does not require transition metals for the photoredox-catalyzed borylation of unreactive C(sp^3^)−H bonds initiated through 1,5-HAT.

## Results and discussion

### Reaction optimization

Recently, visible-light photoredox catalysis has been the leading efficient access to *N*-radicals under mild conditions. With their weak N−O bond, hydroxylamine derivatives have recently been exploited as nitrogen-centered radical precursors in visible-light photocatalysis, given their higher oxidation state, it would be necessary to apply reductive conditions^54,55,57^. Thus, 4-trifluoromethylbenzoyl hydroxamic acid derivative (**1a**) was used as the model substrate to establish optimal reaction parameters, including the equivalency of bis(catecholato)diboron (B_2_cat_2_), the choice of photocatalysts (Ir and Ru complexes, or organic photocatalysts), light sources, and additives (Table 1 and Supplementary Table 1 and Fig. 1). We investigated that with Ir(*p*-CF_3_ppy)_3_ (2%) as photocatalyst, 40 W 456 nm light source, Et_3_N as additive, the desired product was obtained in 45% GC yield. Other amines, such as DABCO, DIPEA or no additive (entry 2–4), were tested, and DIPEA gave improved yield (47%), almost same yield was obtained with a longer reaction time (30 h) (entry 5). Replacing photocatalyst to Ir(ppy)_3_, [Ru(bpy)_3_]Cl_2_, or 4CzIPN could not give the improved results (entry 6–8). Gratifyingly, the reaction gave 47% yield by using Eosin Y, which is a non transition metal photocatalyst, and importantly, much cheaper than Ir(*p*-CF_3_ppy)_3_ (entry 9). Furthermore, We examined the influence of the light source. 467 nm wavelength LED turned out to be better than 456 nm LED, leading a jump increasing yield (from 47% to 73%, entry 10). Higher equivalent photocatalyst (5%) resulted in 77% yield (entry 11). Reducing the B_2_cat_2_ loading from 3.0 to 2.5 equiv. led to a slightly improved yield (81% GC yield, isolated in 73%), and 4.0 equiv. B_2_cat_2_ gave significantly lower yield (entry 13), which showed that the product yield is not always proportional to the equivalent of borylating reagent. Decreasing the loading of additive DIPEA, the yield dropped to 54% (entry 14). Control experiments revealed that no borylation product formed in the absence of photocatalyst, light or additive (entry 15–17). To rule out the potential for substrate thermal decomposition, we conducted a control experiment by replacing the light with heating at 50 °C for 12 h in the absence of light. However, no borylation product was detected under these conditions (entry 18). Continuous irradiation is necessary, as turning off the light after 30 min resulted in no product formation (entry 19). The standard reaction in air gave a 76% yield, indicating that the reaction is not sensitive to oxygen (entry 20).

**Table 1:** Optimization of reaction conditions^a^.

Entry	B_2_cat_2_	PC (mol%)	Light	Additive	Yield^a,b^(%)
1	3.0	Ir(*p*-CF_3_ppy)_3_ (2 mol%)	456 nm	Et_3_N (2.0)	45
2	3.0	Ir(*p*-CF_3_ppy)_3_ (2 mol%)	456 nm	DABCO (2.0)	15
3	3.0	Ir(*p*-CF_3_ppy)_3_ (2 mol%)	456 nm	DIPEA (2.0)	47
4	3.0	Ir(*p*-CF_3_ppy)_3_ (2 mol%)	456 nm	none	trace
5^c^	3.0	Ir(*p*-CF_3_ppy)_3_ (2 mol%)	456 nm	DIPEA (2.0)	49
6	3.0	Ir(ppy)_3_ (2 mol%)	456 nm	DIPEA (2.0)	30
7	3.0	[Ru(bpy)_3_]Cl_2_ (2 mol%)	456 nm	DIPEA (2.0)	trace
8	3.0	4CzIPN (2 mol%)	456 nm	DIPEA (2.0)	trace
9	3.0	Eosin Y (2 mol%)	456 nm	DIPEA (2.0)	47
10	3.0	Eosin Y (2 mol%)	467 nm	DIPEA (2.0)	73
11	3.0	Eosin Y (5 mol%)	467 nm	DIPEA (2.0)	77
12	2.5	Eosin Y (5 mol%)	467 nm	DIPEA (2.0)	81 (73)
13	4.0	Eosin Y (5 mol%)	467 nm	DIPEA (2.0)	40
14	2.5	Eosin Y (5 mol%)	467 nm	DIPEA (1.5)	54
15	2.5	None	467 nm	DIPEA (2.0)	trace
16	2.5	Eosin Y (5 mol%)	none	DIPEA (2.0)	trace
17	2.5	Eosin Y (5 mol%)	467 nm	None	trace
18^d^	2.5	Eosin Y (5 mol%)	none	DIPEA (2.0)	trace
19^e^	2.5	Eosin Y (5 mol%)	467 nm	DIPEA (2.0)	trace
20^f^	2.5	Eosin Y (5 mol%)	467 nm	DIPEA (2.0)	76

^a^Reactions run on 0.2 mmol scale, using Kessil 40 W blue LED as light source.

^b^GC-fid yields are measured by using decane as an internal standard. The yield in bracket is isolated yield.

^c^The reaction time is 30 h.

^d^50 °C for 12 h, in the dark.

^e^Turned off the light after 30 min.

^f^The reaction was set up in air.

### Subtrate scope

**Fig. 3:**
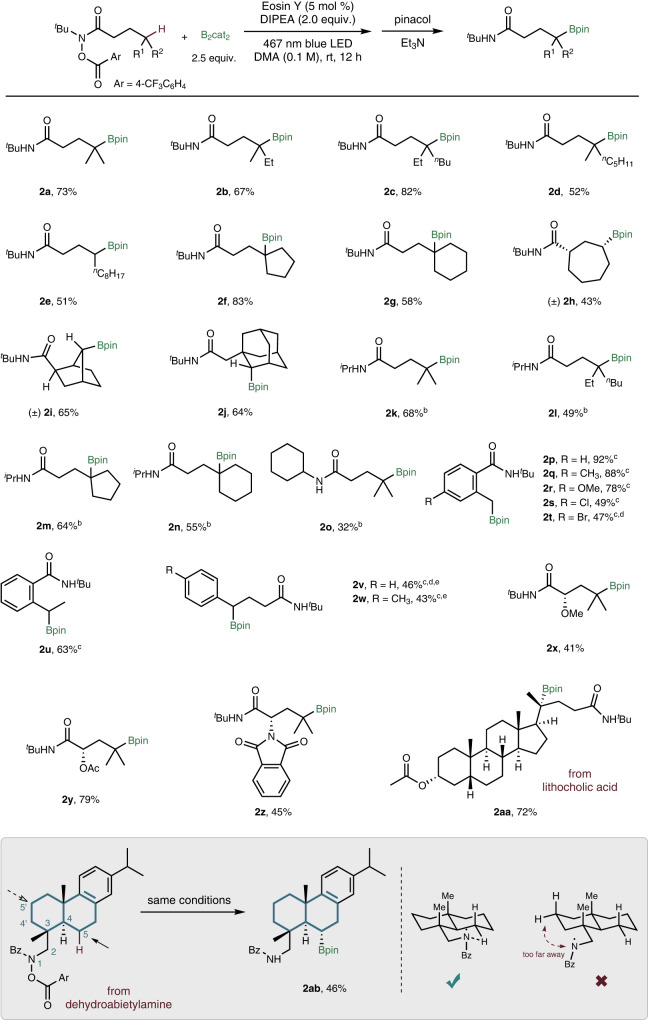
Substrate scope. **a** Reaction conditions: substrate (0.3 mmol), B_2_cat_2_ (0.75 mmol), Eosin Y (5%), DMA (3 mL), DIPEA (0.6 mmol), irradiated by Kessil 40 W blue LED for 12 h, then with pinacol (4.0 equiv.) and Et_3_N (1 mL); **b** Products were formed as mixtures of borylation product and hydrogenation side product; **c** Yields were of isolated products after in-situ oxidation with hydrogen peroxide (4.0 equiv.); **d**^1^H NMR yield was reported. **e** Reaction time was 24 h.

With the optimal reaction conditions, we next sought to explore the substrate scope of this visible light photoredox-catalyzed remote C(sp^3^)-H borylation. As summarized in Fig. 3, a variety of hydroxamic acid derivatives were synthesized and tested. The reactions of the substrates with tertiary C−H bonds in the *γ*-position all gave borylation products in good to excellent yields (**2a**–**2d**), while the reactions of the substrate possessing secondary C−H bonds could also afford moderate yield (**2e,** **2h-2j**). Substrates containing tertiary C−H bond in cyclic structure were also tested, and cyclopentyl (**2f**) and cyclohexyl (**2g**) products were formed in 83% and 58%, respectively. Specially, we found that this method can activate secondary C(sp^3^)-H bonds in cyclic substrates, producing the corresponding syn product **2h** with excellent diastereoselectivity. Moreover, for bicyclic substrate which have more than one *γ*-positions, such as norbornyl derivative, high site-selectivity was obtained (**2i**, 65%; **2j**, 64%) (as indicated by NMR NOE test, see Supplementary Data 2 Figs. 1–3 for details). With the replacement of tertbutyl group to isopropyl group or cyclohexyl group, the substrates can undergo the desired transformation to form borylation products in moderate to good yields (**2k**-**2o**), albeit the undesired hydrogenation by-products accompanied. It may cause by replacing the *N*-connected alkyl group from tertiary one to secondary one, which weakens the N−H bond, and the 1,5-HAT process was affected thermodynamically (vide infra, a DFT calculation was performed). Interestingly, we found that a benzene ring can be involved in this six-membered ring 1,5-HAT process, as exemplified in the cases of (**2p**-**2u**). For isolation of these products, we found the partial instability of the benzylic pinacol boronic esters in silica gel, and that in situ oxidation with H_2_O_2_ allowed isolation of the corresponding alcohols in high yields. Various functional groups such as methyl(**2q**), methoxy (**2r**), chlorine (**2s**) and bromine (**2t**) were well tolerated, and the yields were up to 92%. For secondary benzylic substrates(**2u**) or benzylic position on the carbon chain substrates(**2v**-**2w**), the products were obtained in good yields. *α*-hydroxy hydroxamic acids derivatives were also tested, including methoxy (**2x**), acetoxy (**2y**). Amino acid leucine-derived hydroxamic acid also underwent this remote borylation, giving the desired product in 45% (**2z**). Our method can also work on complex molecules, such as lithocholic acid and dehydroabietylamine derivatives (in the cases of **2aa** and **2ab**). It is worth noting that the last example (**2ab**) in the substrate scope is a different type other than the rest ones. The borylation of C−H bond took place on the alkyl chain of the amine site.

### Mechanistic studies

**Fig. 4:**
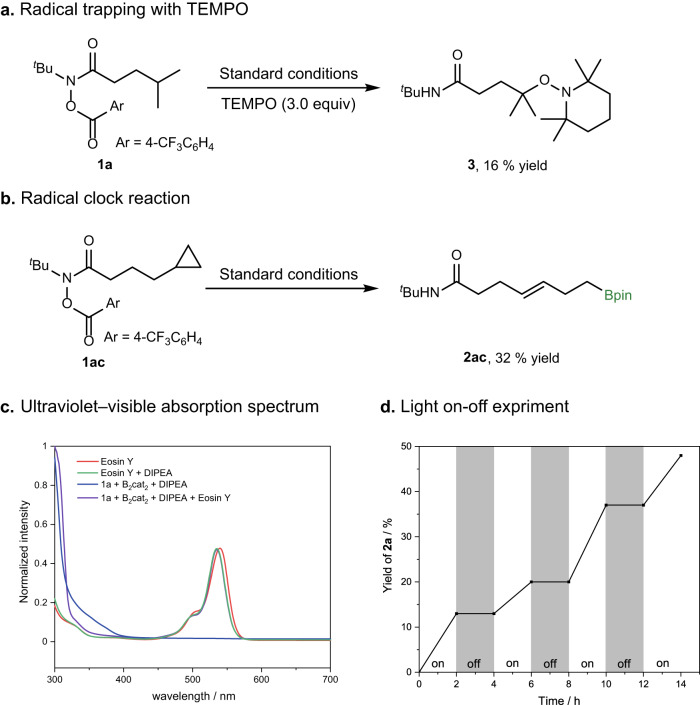
Mechanistic studies. **a** The standard reaction with TEMPO (3.0 equiv.) as a radical scavenger. **b** Radical clock ring-opening reaction of **1ac**. **c** In the ultraviolet-visible absorption spectrum, the photocatalyst Eosin Y was found to be the only absorbing species in the reaction near the excitation wavelength (*λ*_*max*_ = 467 nm). **d** The light on-off expriment showed no product formation in dark.

To gain further insight into the mechanism of this 1,5-HAT borylation reaction, we performed a series of mechanistic investigations. First, 2,2,6,6-tetramethyl-1-piperidinyloxy (TEMPO) was added to standard conditions, and the reaction yielded 16% of the corresponding TEMPO-trapped product **3** as a white solid, instead of the desired borylation product (Fig. 4a). Second, we designed and managed to synthesize the remote cyclopropyl substrate **1ac**, and the reaction of **1ac** in standard conditions gave the ring-opened terminal borylation product in 32% yield (Fig. 4b). These two experiments indicated the involvement of carbon radical intermediates in this borylation reaction. The photocatalyst Eosin Y was found to be the only absorbing species in the reaction near the excitation wavelength (*λ*_*max*_ = 467 nm) from ultraviolet-visible absorption spectroscopy (Fig. 4c and Supplementary Fig. 2). To determine whether the reaction involves an efficient radical chain mechanism, we performed a light on- off experiment showing no product formation in the dark phases (Fig. 4d and Supplementary Table 2 and Fig. 3).

**Fig. 5:**
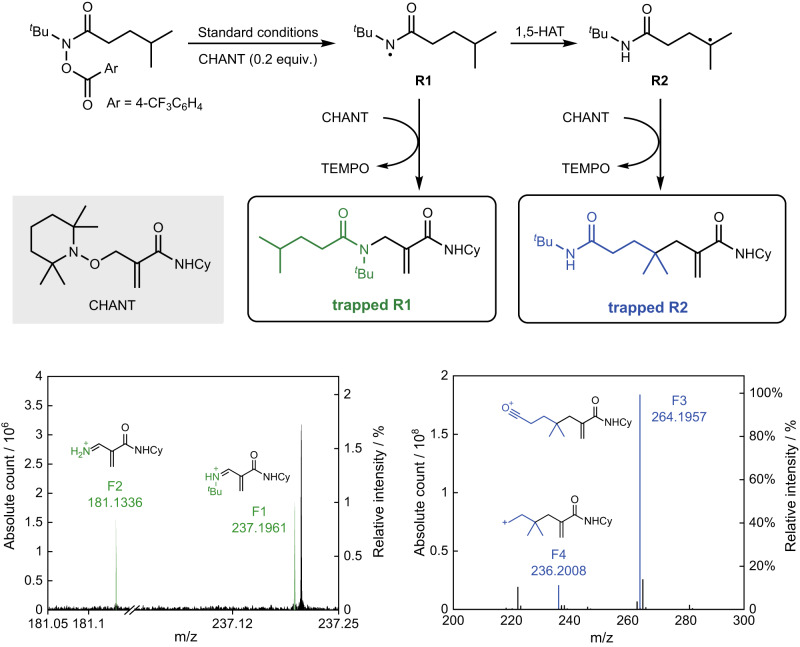
The radical trapping experiment with CHANT and MS analysis. **R1**: carbon-centered radical. **R2**: nitrogen-centered radical. Trapped **R1**: **R1** trapped by CHANT. Trapped **R2**: **R2** trapped by CHANT. CHANT: N-cyclohexyl-2-[(2,2,6,6-tetramethylpiperidin-1-yl)oxy]methylacrylamide. Cy = cyclohexyl. The fragments F1 and F2 were attributed to trapped **R1**; The fragments F3 and F4 were attributed to trapped **R2**.

**Fig. 6:**
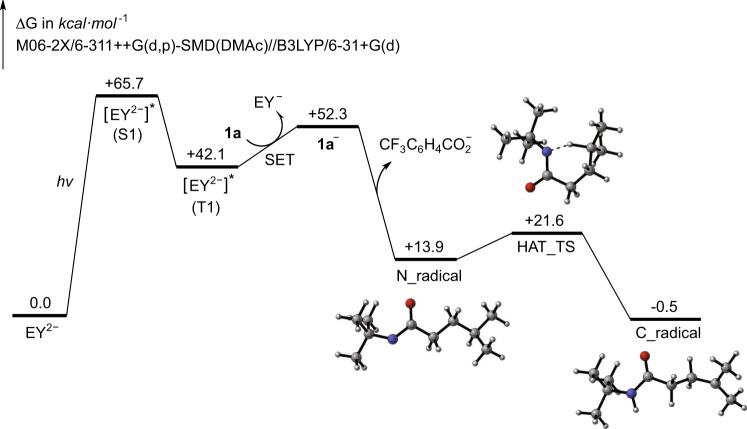
DFT-computed pathways for the generation of the *N*-centered radical and 1,5-HAT process. **1a** is reducted by EY^2−^(T1)* to give 1a^−^, followed the leaving of ${{{{{{{{\rm{CF}}}}}}}}}_{3}{{{{{{{{\rm{C}}}}}}}}}_{6}{{{{{{{{\rm{H}}}}}}}}}_{4}{{{{{{{{\rm{CO}}}}}}}}}_{2}^{-}$ group, which is thermodynamically favorable. The N_radical overcomes a relatively low barrier (7.7 kcal ⋅ mol^−1^) to give C_radical. The Gaussian 09 level of M06-2X/6-311++G(d,p)-SMD(DMAc)//B3LYP/6-31+G(d) was used.

**Fig. 7:**
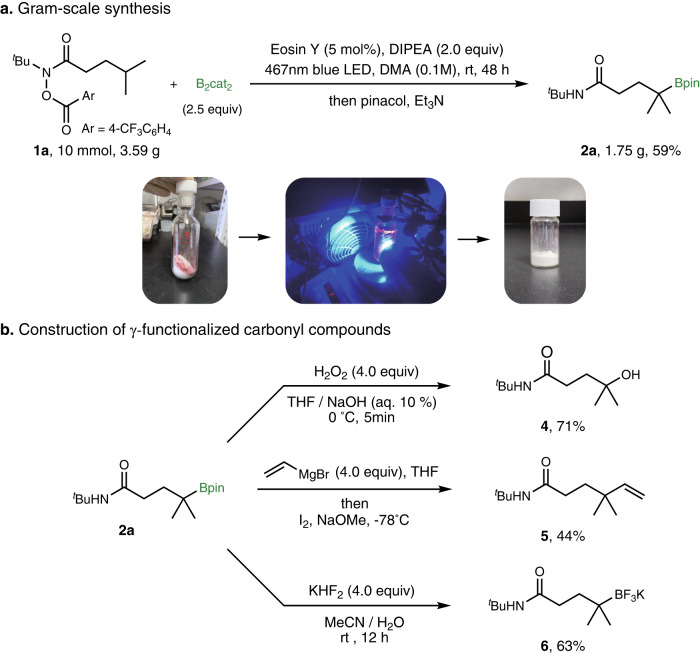
The gram-scale synthesis and further transformations of the borylation products. **a** Gram-scale synthesis set up in air, resulting 1.59 g **2a** as a white solid, the following pictures depict the step-by-step procedure involved in the synthesis. **b** Conversion of **2a** into *γ*-functionalized carbonyl compounds: oxidation to alcohol; vinylation of boronic ester; transformation to potassium trifluoroborate salts.

In recent days, the Chechik group reported a new type of radical scavenger called CHANT to detect short-lived radical intermediates (Supplementary Fig. 4)^69^. In this method, the radical trapping product is a bench-stable terminal alkene that can be analyzed by mass spectrometry (MS). To further testify the 1,5-HAT process, we chose CHANT as radical trapping reagent, and carried out the reaction in standard conditions (Fig. 5). The trapped radicals were then analyzed by electrospray ionization HRMS (Supplementary Tables 3, 4 and Figs. 5–7). The MS peak at m/z 337.2849 indicates the trapped radical intermediates, whereas it cannot distinguish the nitrogen-centered radical **R1** or the carbon-centered radical **R2**, since they are isomers. In order to distinguish them, the tandem mass spectrometry was analyzed at m/z 337.2849, the two low-intensity peaks F1 (m/z 237.1961) and F2 (m/z 181.1336) which contain two nitrogen atoms, could only be attributed to the trapped **R1**, the fragments showed that the acyl group RCO was dissociated. The most strong-intensity peaks F3 (m/z 264.1957) is a stable acylium ions, and F4 (m/z 236.2008) is F3 losing CO. They could only be attributed to the trapped **R2**, and many other strong peaks could also be attributed to it (see Supplementary Table 4 for details). This experiment proved that the existence of **R1** and **R2**. The intensity of the trapped **R2** is 100 times greater than the trapped **R1**. Assuming a similar ionization efficiency of the trapped **R1** and **R2** species, this suggests that the 1,5-HAT process is very fast and that the carbon-centered radical **R2** is the resting state for this radical process.

To better understand the photoredox mechanism of this 1,5-HAT process, we carried out density functional theory (DFT) calculations (see Supplementary Information section 6 for details). The computed free energy profile of the key steps in the photo irradiation process is shown in Fig. 6. Eosin Y transfers from ground state EY^2−^ to excited singlet state ${[{{{{{{{{\rm{EY}}}}}}}}}^{2-}]}^{* }$ (S1) upon being irradiated with the vertical excitation energy of +65.7 kcal mol^−1^ (equals to 2.8 eV, corresponds to the energy of 442 nm wavelength light)^70–72^. Then this singlet state transforms to its more stable triplet counterpart ${[{{{{{{{{\rm{EY}}}}}}}}}^{2-}]}^{* }$ (T1)^73^. Next, this triplet state species is oxidatively quenched by **1a** to give EY^−^ and **1a**^−^, which further undergoes N−O bond cleavage to form a nitrogen-centered radical, accompanied with the leaving of ${{{{{{{{\rm{CF}}}}}}}}}_{3}{{{{{{{{\rm{C}}}}}}}}}_{6}{{{{{{{{\rm{H}}}}}}}}}_{4}{{{{{{{{\rm{CO}}}}}}}}}_{2}^{-}$ group. The N_radical overcomes a relatively low barrier (7.7 kcal mol^−1^) to the six-member ring transition state HAT_TS, which allows the hydrogen atom to transfer from *C* atom to *N* atom to give the C_radical, this step is quite favorable thermodynamically. Subsequently, the C_radical is borylated by diboron to give the desired product^59^.

As shown in substrate scope studies, it is noted that the borylation reactivity is in a order of tertiary C−H bond substrates > secondary ones > primary ones. For tertiary C−H bond substrates, the reactivity of the *N-tert*-butyl substituted ones are better than that of the *N*-isopropyl ones. To further elucidate these reactivity patterns, we calculated the standard Gibbs free energy and the activation Gibbs free energy in the process of 1,5-HAT of radicals **I** to **IV** (Table 2 and Supplementary Tables 5–7). Our results indicate that the tertiary C−H bonds are the most thermodynamically and dynamically favorable for 1,5-HAT process, due to the more stability effect of *σ*-*p* hyperconjugation. When replacing tertbutyl with isopropyl in tertiary C−H bond substrates, the activation energy raises from 7.7 to 10.8 kcal mol^−1^ (**IV**), leading to the formation of a certain amount of hydrogenation product.Similar behavior was observed for secondary C−H bond substrates, where the activation energy increases from 7.7 to 10.4 kcal mol^−1^ (**II**). For the primary C−H bond substrates, the barrier is 13.1 kcal mol^−1^ (**I**), the reaction all gave hydrogenation products and no borylation products were obtained.

**Table 2:** The correlation between the type of remote C−H bonds and reaction parameters.

kcal mol^−1^	I	II	III	IV
ΔG^Θ^	−8.3	−12.2	−14.4	−14.7
ΔG^‡^	13.1	10.4	7.7	10.8

To further demonstrate the utility of this borylation method, a gram-scale reaction of **1a** was carried out under air (Fig. 7a and Supplementary Fig. 8), furnishing **2a** in 59% isolated yield. The resulting borylation products could be further transformed to a series of functional groups (Fig. 7b). For example, the tertiary alcohol in *γ*-position of carbonyl compounds could be obtained by oxidation of the boron compound **2a**. In addition, tertiary boronic esters underwent C−C bond formation with vinyl Grignard reagent to afford vinylation products **5**^74^. Treatment of **1a** with KHF_2_ yields the potassium trifluoroborate salt **6** in 63% yield.

**Fig. 8:**
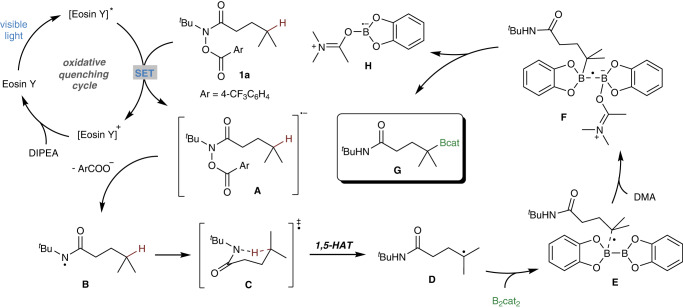
Proposed mechanism for the remote C(sp^3^)−H borylation reaction enabled by 1,5-HAT process. The carbon radical **D** is then borylated by B_2_cat_2_/DMA to give **G**.

Based on the experimental and computational results as well as studies on previously developed radical borylation reactions^59,62,75–79^, a proposed mechanism for the photoredox-catalyzed 1,5-HAT borylation process is presented in Fig. 8. First, substrate **1a** is reducted by exicited photocatalyst Eosin Y to give radical **A**, followed by N−O bond cleavage to give nitrogen-center radical **B**, due to the higer BDE of N−H bond, radical **B** forms the six-membered ring transition state **C** to allow intramolecular 1,5-HAT process to give carbon-centered radical **D**, which adds to diboron B_2_cat_2_ to deliver the adduct **E**, with the help of DMA, the adduct **F**’s B−B bond is dissociated to form C−B bond, affords the borylated product **G**, along with the DMA-Bcat radical **H**.

## Conclusion

In conclusion, we have developed a transition-metal free visible light photoredox-catalyzed remote C(sp^3^)-H borylation reaction, which enabled by 1,5-HAT process based on hydroxamic acid derivatives. A series of mechanism experiments, as well as DFT calculations resolved the radical mechanism in detail. This investigation afforded remote C−H bond functionalization of *γ* position of carbonyl compounds, thus provided scarce synthetic method of such type. We anticipate that this approach will find broad applications in synthetic community.

## Methods

### General procedure of C(sp^3^)−H borylation enabled by 1,5-HAT

In a glovebox under a nitrogen atmosphere, sequentially added B_2_cat_2_ (0.75 mmol, 1.5 equiv., 178 mg), the substrate (0.3 mmol, 1.0 equiv.), Eosin Y (5%, 0.015 mmol, 10.5 mg) and 3 mL DMA to a 10 mL Schlenk tube with a stir bar, followed by DIPEA (0.6 mmol, 2.0 equiv., 105 μL). The capped Schlenk tube was removed from the glovebox, and the reaction mixture was irradiated by 467 nm Kessil 40 W LED at a 3 cm distance for 12 h, with a fan for cooling. As the catechol boronate esters are sensitive to hydrolysis, RBcat was transformed to RBpin for isolation. After cooling to room temperature, pinacol (142 mg, 4.0 equiv.) and 1.0 mL triethylamine were added, and the mixture was stirred at room temperature for 1 h. Water was added to the reaction mixture, the aqueous layer was extracted with EtOAc (20 mL × 2). If phase separation was slow, brine was added. The organic phases were combined and washed with 50 mL of saturated brine, the organic phase was dried over anhydrous sodium sulfate and filtered, then concentrated under reduced pressure, purified by column chromatography to give the product. The product was monitored by thin-layer chromatography with phosphomolybdic acid stain.

## Supplementary information

Supplementary Information

Description of Additional Supplementary Files

Supplementary Data 1

Supplementary Data 2

## Data Availability

The authors declare that the data supporting the study are available within the article and Supplementary Information. For experimental details and compounds characterization, see Supplementary Information. For Cartesian coordinates of the structures, see Supplementary Data 1. For NMR spectra, see Supplementary Data 2.
