# Supplementary material for: Transition metal-free visible light photoredox-catalyzed remote C(sp3)−H borylation enabled by 1,5-hydrogen atom transfer
Source: Commun Chem. 2023 Jul 24;6:156. doi: 10.1038/s42004-023-00960-z (PMC10366130; doi:10.1038/s42004-023-00960-z)
Supplement: Supplementary file 1 — Supplementary Information [file 42004_2023_960_MOESM1_ESM.docx]

**Supplementary Information**

**Transition metal-free Visible Light Photoredox-catalyzed Remote C(sp^3^)−H Borylation Enabled by 1,5-Hydrogen Atom Transfer**

Beiqi Sun^1,2^, Wenke Li^2^, Qianyi Liu^2^, Gaoge Zhang^2^ and Fanyang Mo^1^*

*1* School of Materials Science and Engineering, Peking University, Yiheyuan Road, Beijing, 100871, China.*

*2 College of Engineering, Peking University, Yiheyuan Road, Beijing, 100871, China.*

**Corresponding author(s). E-mail(s): fmo@pku.edu.cn.*

**Contents**

[**Supplementary Methods** 3](#_Toc139720528)

[1. General information 3](#_Toc139720529)

[1.1 Structures of hydroxamic acid derivatives 4](#_Toc139720530)

[1.2 Structures of borylation products 5](#_Toc139720531)

[2. Preparation of substrates 6](#_Toc139720532)

[2.1 General procedure 6](#_Toc139720533)

[2.2 Characterization of substrates 7](#_Toc139720534)

[3. Reaction optimization 20](#_Toc139720535)

[4. Experimental procedures and characterization data 22](#_Toc139720536)

[4.1 General procedure 22](#_Toc139720537)

[4.2 Characterization of products 24](#_Toc139720538)

[4.3 Failed examples 38](#_Toc139720539)

[5. Mechanism studies 39](#_Toc139720540)

[5.1 Reaction with TEMPO 39](#_Toc139720541)

[5.2 Radical clock reaction 40](#_Toc139720542)

[5.3 Ultraviolet–visible absorption spectroscopy 42](#_Toc139720543)

[5.4 Light on-off experiment 43](#_Toc139720544)

[5.5 Reaction with radical trapping reagent CHANT and mass spectrum analysis 45](#_Toc139720545)

[6. DFT computational data 50](#_Toc139720546)

[6.1 Table of energies and lowest frequencies 50](#_Toc139720547)

[6.2 TD-DFT Computational data 52](#_Toc139720548)

[7. Further transformation of products and gram-scale synthesis 53](#_Toc139720549)

[7.1 Oxidation to alcohol 53](#_Toc139720550)

[7.2 Vinylation of boronic ester 54](#_Toc139720551)

[7.3 Transformation to potassium trifluoroborate salts 55](#_Toc139720552)

[7.4 Gram-scale synthesis 56](#_Toc139720553)

[**Supplementary References** 57](#_Toc139720554)

# Supplementary Methods

## General information

Unless otherwise noted, all experiments were carried out under an inert atmosphere in a nitrogen-filled glovebox or by standard Schlenk techniques. The glassware were purchased from SYNTHWARE. Solvents were purchased from TONGGUANG CHEMICAL, Beijing or BEIJING CHEMICAL, in GR (or CCER). Reaction solvents were purchased from J&K Scientific and stored in a nitrogen-filled glovebox over 4Å molecular sieves. Bis(catecholato)diboron (B_2_cat_2_) was purchased from Beijing Innochem Science & Technology co., LTD (Innochem) and stored in the glovebox. Purification of the products was conducted by column chromatography on silica gel (200 - 300 mesh, in some cases 300-400 mesh were used, from Qingdao, China). Thin-layer chromatography (TLC) was performed on silica gel plates (10 - 40 μm) purchased from WISH CHEMICAL, using UV light (254/ 366 nm) or phosphomolybdic acid (PMA) in ethanol (5%) for detection. The substrates were purchased from commercial sources unless otherwise noted.

NMR spectra were measured on a Bruker ARX400 (^1^H at 400 MHz, ^13^C at 101 MHz) magnetic resonance spectrometer. All spectral data was acquired at 295 K. Deuterated solvents were purchased from Cambridge Isotope Laboratories (CDCl_3_, deuteration > 99.8%, +0.03% V/V TMS) or Aldrich (DMSO-d6, deuteration > 99.9 %). The chemical shifts are reported as parts per million (ppm) referenced to residual protium or carbon of the solvents, CHCl_3_, δH (7.26 ppm) and δC (77.00 ppm), DMSO-d6 (δH 2.50 ppm) and (δC 39.5 ppm); Coupling constants are reported in Hertz (Hz). Data for ^1^H NMR spectra are reported as follows: chemical shift (ppm, referenced to protium; s = singlet, d = doublet, t = triplet, q = quartet, dd = doublet of doublets, td = triplet of doublets, ddd = doublet of doublet of doublets, m = multiplet, coupling constant (Hz), and integration). XRD data were collected by XtaLAB PRO 007HF(Cu) Single crystal X-ray diffractometer. Infrared spectra were recorded on a Thermal Fisher Nicolet iS50 Fourier transform spectrometer (FT-IR) and were reported in wave numbers (cm^-1^). HRMS data were obtained on a solariX XR Fourier Transform Ion Cyclotron Resonance Mass Spectrometer. Mass spectra of trapping reactions were recorded using positive electrospray ionization (Pos ESI) on a high resolution solariX XR FTMS mass spectrometer. UV-vis absorption spectrum was measured by a UV-visible spectrophotometer (Thermo Evolution^TM^ 220). GC-MS or FID data were measured using the Agilent Technologies 7890B GC and the Agilent Technologies 5977B MSD. The FID yields were all based on standard curves with 5 points and minimum 0.996 R^2^ value (or 4 points and minimum 0.997 R^2^ value).

### 1.1 Structures of hydroxamic acid derivatives

### 1.2 Structures of borylation products

## Preparation of substrates

### 2.1 General procedure

**The procedure was according to literature^1^.**

**Step 1:** To a solution of carboxylic acid (1.0 equiv.) and 3-5 drops of anhydrous DMF in anhydrous CH_2_Cl_2_ (0.5 M) at 0 ˚C, oxalyl chloride (1.5 equiv.) was added dropwise over 10 minutes. The reaction was vigorously stirred at room temperature for 3 h. The solvent was removed in vacuum. Anhydrous CH_2_Cl_2_ was added to remove the residual oxalyl chloride in vacuum. Then the resulting acyl chloride was dissolved in anhydrous DCM and used directly for the next step without further purification.

**Step 2:** A solution of the N-(tert-butyl)hydroxylamine hydrochloride (1.0 equiv., or N-isopropylhydroxylamine hydrochloride for **1k-1n**, or N-cyclohexylhydroxylamine hydrochloride for **1o**) in anhydrous THF (0.4 M) was cooled to 0 ºC, treated with DIPEA (2.0 equiv.) and stirred for 15 minutes. The acyl chloride (1.0 equiv.) in anhydrous acetonitrile was added dropwise over 15 minutes. The mixture was allowed to warm to room temperature overnight. The mixture was diluted with saturated NaHCO_3_ and EtOAc, the layers were separated. The aqueous layer was extracted with EtOAc (2 x), the combined organic layers were washed with 1 M HCl, saturated NaHCO_3_ and brine, successively, and then evaporated. Purification by column chromatography on silica gel eluting with Petroleum ether and EtOAc gave the hydroxamic acid.

**Step 3:** To a solution of hydroxamic acid (1.05 equiv.) in anhydrous CH_2_Cl_2_ (0.35 M) at 0 ˚C, Et_3_N (1.5 equiv.) was added dropwise. 4-trifluoromethyl-benzoyl chloride (1.0 equiv.) was then added dropwise over 5 minutes. The reaction was vigorously stirred at room temperature for 2 h. After removing the solvent, the resulting residue was added saturated NaHCO_3_ and THF and stirred for 30 minutes. Then, the layers were separated. The aqueous layer was extracted with EtOAc again, the combined organic layers were washed with 1 M HCl, saturated NaHCO_3_ and brine, successively, and then evaporated. Purification by column chromatography gave **1a-1aa**.

### 2.2 Characterization of substrates

**N-(tert-butyl)-4-methyl-N-((4-(trifluoromethyl)benzoyl)oxy)pentanamide (1a)**

76% overall yield, white solid.

**^1^H NMR** (400 MHz, CDCl_3_) δ 8.23 (d, J = 8.1 Hz, 2H), 7.80 (d, J = 8.2 Hz, 2H), 2.26 (dt, J = 15.0, 7.1 Hz, 1H), 2.10 (dt, J = 18.1, 7.0 Hz, 1H), 1.48 (s, 12H), 0.84 –0.76 (m, 6H).

**^13^C NMR** (101 MHz, CDCl_3_) δ 175.1, 164.6, 135.9 (q, J = 33.0 Hz), 130.5, 130.3, 126.1 (q, J = 3.7 Hz), 123.4 (q, J = 272.9 Hz), 63.0, 33.0, 32.4, 27.6, 27.6, 22.5, 22.4.

All data matched that reported in the literature^1^.

**N-(tert-butyl)-4-methyl-N-((4-(trifluoromethyl)benzoyl)oxy)hexanamide (1b)**

32% overall yield, white solid.

**^1^H NMR** (400 MHz, CDCl_3_) δ 8.24 (d, J = 8.3 Hz, 2H), 7.81 (d, J = 8.3 Hz, 2H), 2.34 – 2.19 (m, 1H), 2.18 – 2.03 (m, 1H), 1.70 – 1.58 (m, 2H), 1.49 (s, 9H), 1.42 – 1.40 (m, 1H), 1.32 – 1.22 (m, 2H), 0.86 – 0.74 (m, 6H).

**^13^C NMR** (101 MHz, CDCl_3_) δ 175.2, 164.6, 135.9 (d, J = 32.9 Hz), 130.5, 130.3, 126.2 (q, J = 3.9 Hz), 123.5 (d, J = 272.9 Hz), 63.0, 34.0, 30.8, 29.5, 27.6, 19.0, 11.4.

**HRMS** (ESI) (m/z): Calcd. C_19_H_27_F_3_NO_3_^+^ [M+H]^+^ : 374.1938. Found: 374.1944.

**IR** (ν/ cm^−1^, neat): 2962, 2919, 1756, 1678, 1461, 1414, 1365, 1325, 1256, 1241, 1204, 1168, 1131, 1065, 1013, 934, 864, 772, 703.

**N-(tert-butyl)-4-ethyl-N-((4-(trifluoromethyl)benzoyl)oxy)octanamide (1c)**

37% overall yield, colorless oil.

**^1^H NMR** (400 MHz, CDCl_3_) δ 8.23 (d, J = 8.1 Hz, 2H), 7.81 (d, J = 8.2 Hz, 2H), 2.23 (dt, J = 16.1, 8.0 Hz, 1H), 2.09 (dt, J = 15.8, 8.0 Hz, 1H), 1.59 – 1.53 (m, 2H), 1.49 (s, 9H), 1.23 – 1.11 (m, 9H), 0.85 – 0.75 (m, 6H).

**^13^C NMR** (101 MHz, CDCl_3_) δ 175.2, 164.6, 135.8 (q, J = 33.0 Hz), 130.4, 130.3, 126.1 (q, J = 3.7 Hz), 123.4 (q, J = 272.9 Hz), 62.9, 38.4, 32.6, 32.0, 28.8, 27.7, 27.5, 25.7, 23.0, 14.0, 10.7.

All data matched that reported in the literature^1^.

**N-(tert-butyl)-4-methyl-N-((4-(trifluoromethyl)benzoyl)oxy)nonanamide (1d)**

18% overall yield, colorless oil.

**^1^H NMR** (400 MHz, CDCl_3_) δ 8.24 (d, J = 8.1 Hz, 2H), 7.81 (d, J = 8.3 Hz, 2H), 2.32 – 2.18 (m, 1H), 2.17 – 2.05 (m, 1H), 1.72 – 1.57 (m, 2H), 1.49 (s, 9H), 1.42 (s, 1H), 1.30 – 1.11 (m, 8H), 0.87 – 0.74 (m, 6H).

**^13^C NMR** (101 MHz, CDCl_3_) δ 175.2, 164.6, 135.8 (q, J = 32.6 Hz), 126.0 (q, J = 3.7 Hz), 123.5 (q, J = 273.0 Hz), 62.9, 33.3, 30.9, 30.1, 27.7, 27.6, 26.6, 25.6, 25.3, 22.7, 22.4.

**HRMS** (ESI) (m/z): Calcd. C_22_H_33_F_3_NO_3_^+^ [M+H]^+^ : 416.2407. Found: 416.2409.

**IR** (ν/ cm^−1^, neat): 2958, 2927, 2859, 1769, 1679, 1513, 1460, 1412, 1365, 1323, 1235, 1204, 1173, 1134, 1114, 1065, 1045, 1011, 933, 862, 770, 701, 592.

**N-(tert-butyl)-N-((4-(trifluoromethyl)benzoyl)oxy)dodecanamide (1e)**

67% overall yield, white solid.

**^1^H NMR** (400 MHz, CDCl_3_) δ 8.23 (d, J = 8.2 Hz, 2H), 7.81 (d, J = 8.2 Hz, 2H), 2.27 (dt, J = 16.1, 8.1 Hz, 1H), 2.10 (dt, J = 15.3, 7.1 Hz, 1H), 1.49 (s, 9H), 1.33 – 1.19 (m, 22H).

**^13^C NMR** (101 MHz, CDCl_3_) δ 175.0, 164.6, 127.7, 126.1 (q, J = 3.6 Hz), 123.5 (q, J = 272.7 Hz), 34.4, 32.0, 29.7, 29.6, 29.5, 29.4, 29.3, 27.6, 27.6, 24.2, 22.8, 14.2.

**HRMS** (ESI) (m/z): Calcd. C_24_H_37_F_3_NO_3_^+^ [M+H]^+^ : 444.2720. Found: 444.2719.

**IR** (ν/ cm^−1^, neat): 2958, 2873, 1771, 1676, 1586, 1513, 1467, 1411, 1391, 1369, 1323, 1236, 1170, 1130, 1065, 1050, 1008, 861, 768, 700, 592.

**N-(tert-butyl)-3-cyclopentyl-N-((4-(trifluoromethyl)benzoyl)oxy)propanamide** **(1f)**

54% overall yield, white solid.

**^1^H NMR** (400 MHz, CDCl_3_) δ 8.23 (d, J = 8.1 Hz, 2H), 7.79 (d, J = 8.3 Hz, 2H), 2.28 (dt, J = 15.3, 7.5 Hz, 1H), 2.11 (dt, J = 15.7, 7.5 Hz, 1H), 1.71 – 1.53 (m, 5H), 1.52 – 1.37 (m, 13H), 1.05 – 0.92 (m, 2H).

**^13^C NMR** (101 MHz, CDCl_3_) δ 171.6, 164.4, 160.0, 135.3 (q, J = 33.0 Hz), 130.3, 130.0, 128.7, 127.8, 125.7 (q, J = 3.9 Hz), 123.4 (q, J = 272.9 Hz), 115.7, 110.5, 63.1, 55.2, 27.8, 19.4.

All data matched that reported in the literature^1^.

**N-(tert-butyl)-3-cyclohexyl-N-((4-(trifluoromethyl)benzoyl)oxy)propanamide** **(1g)**

70% overall yield, white solid.

**^1^H NMR** (400 MHz, CDCl_3_) δ 8.22 (d, J = 8.1 Hz, 2H), 7.79 (d, J = 8.2 Hz, 2H), 2.26 (dt, J = 15.8, 7.8 Hz, 1H), 2.10 (dt, J = 15.8, 7.7 Hz, 1H), 1.66 – 1.52 (m, 5H), 1.52 – 1.40 (m, 11H), 1.20 – 1.01 (m, 4H), 0.91 – 0.71 (m, 2H).

**^13^C NMR** (101 MHz, CDCl_3_) δ 175.3, 164.6, 135.9 (q, J = 33.0 Hz), 130.5, 130.3, 126.1 (q, J = 3.7 Hz), 123.4 (q, J = 272.9 Hz), 62.9, 37.2, 33.2, 33.2, 31.8, 31.6, 27.6, 26.6, 26.3.

All data matched that reported in the literature^1^.

**N-(tert-butyl)-N-((4-(trifluoromethyl)benzoyl)oxy)cycloheptanecarboxamide (1h)**

17% overall yield, white solid.

**^1^H NMR** (400 MHz, CDCl_3_) δ 8.22 (d, J = 8.1 Hz, 2H), 7.80 (d, J = 8.4 Hz, 2H), 2.34 – 2.22 (m, 1H), 1.93 – 1.63 (m, 5H), 1.63 – 1.53 (m, 1H), 1.47 (s, 13H), 1.33 – 1.17 (m, 2H).

**^13^C NMR** (101 MHz, CDCl_3_) δ 179.0, 164.7, 135.9 (q, J = 33.0 Hz), 126.2 (q, J = 3.7 Hz), 123.5 (q, J = 273.1 Hz), 62.9, 43.8, 31.0, 30.6, 28.2, 27.7, 26.9, 26.6.

**HRMS** (ESI) (m/z): Calcd. C_20_H_27_F_3_NO_3_^+^ [M+H]^+^ : 386.1938. Found: 386.1932.

**IR** (ν/ cm^−1^, neat): 2918, 2858, 1759, 1669, 1457, 1416, 1393, 1363, 1324, 1243, 1169, 1125, 1116, 1068, 1049, 1012, 869, 771, 701.

**N-(tert-butyl)-N-((4-(trifluoromethyl)benzoyl)oxy)bicyclo[2.2.1]heptane-2-carboxamide (1i)**

16% overall yield, white solid.

**^1^H NMR** (400 MHz, CDCl_3_) δ 8.26 (d, J = 8.3 Hz, 2H), 7.82 (d, J = 8.3 Hz, 2H), 2.60 – 2.50 (m, 1H), 2.21 (t, J = 4.8 Hz, 1H), 1.87 – 1.70 (m, 1H), 1.59 – 0.95 (m, 17H).

**^13^C NMR** (101 MHz, CDCl_3_) δ 175.7, 164.8, 135.8 (d), 130.5, 130.4, 126.2 (q, J = 3.7 Hz), 123.5 (d, J = 272.9 Hz), 63.3, 46.0, 41.0, 40.0, 37.1, 31.9, 29.0, 27.7, 27.6, 24.6.

**HRMS** (ESI) (m/z): Calcd. C_20_H_25_F_3_NO_3_^+^ [M+H]^+^ : 384.1781. Found: 384.1785.

**IR** (ν/ cm^−1^, neat): 2965, 2872, 1758, 1672, 1513, 1455, 1413, 1364, 1325, 1237, 1169, 1132, 1066, 1051, 1012, 941, 855, 768, 736, 717, 700, 675, 634.

**2-adamantantyl-N-(tert-butyl)-N-((4-(trifluoromethyl)benzoyl)oxy)acetamide (1j)**

11% overall yield, white solid.

**^1^H NMR** (400 MHz, CDCl_3_) δ 8.22 (d, J = 7.9 Hz, 2H), 7.80 (d, J = 8.2 Hz, 2H), 2.01 – 1.82 (m, 5H), 1.72 – 1.56 (m, 13H), 1.48 (s, 9H).

**^13^C NMR** (101 MHz, CDCl_3_) δ 172.7, 164.7, 135.9 (q, J = 33.0 Hz), 130.5, 130.3, 126.1 (q, J = 3.7 Hz), 123.4 (q, J = 273.0 Hz), 63.1, 47.5, 42.7, 36.9, 33.6, 28.8, 27.8.

All data matched that reported in the literature^1^.

**N-isopropyl-4-methyl-N-((4-(trifluoromethyl)benzoyl)oxy)pentanamide (1k)**

77% overall yield, white solid.

**^1^H NMR** (400 MHz, CDCl_3_) δ 8.24 (d, J = 8.3 Hz, 2H), 7.80 (d, J = 8.4 Hz, 2H), 4.82 (s, 1H), 2.41 – 2.15 (m, 2H), 1.56 (t, J = 5.9 Hz, 3H), 1.25 (d, J = 6.7 Hz, 6H), 0.86 (d, J = 6.1 Hz, 6H).

**^13^C NMR** (101 MHz, CDCl_3_) 164.0, 135.7 (q, J = 32.7 Hz), 130.5, 130.4, 126.0 (q, J = 3.6 Hz), 123.4 (q, J = 272.9 Hz), 51.4 – 49.8 (m), 33.2, 31.0, 27.7, 22.3, 19.5.

**HRMS** (ESI) (m/z): Calcd. C_17_H_23_F_3_NO_3_^+^ [M+H]^+^ : 346.1625. Found: 346.1618.

**IR** (ν/ cm^−1^, neat): 2958, 2873, 1771, 1676, 1513, 1467, 1411, 1391, 1323, 1236, 1170, 1130, 1065, 1051, 1009, 861, 768, 700.

**4-ethyl-N-isopropyl-N-((4-(trifluoromethyl)benzoyl)oxy)octanamide (1l)**

22% overall yield, colorless oil.

**^1^H NMR** (400 MHz, CDCl_3_) δ 8.16 (d, J = 8.3 Hz, 2H), 7.72 (d, J = 8.4 Hz, 2H), 4.74 (s, 1H), 2.18 (t, J = 8.2 Hz, 2H), 1.54 (td, J = 8.4, 5.3 Hz, 2H), 1.25 – 1.05 (m, 15H), 0.74 (q, J = 7.0 Hz, 6H).

**^13^C NMR** (101 MHz, CDCl_3_) δ 164.1, 135.8 (q, J = 33.4 Hz), 130.5, 126.0 (q, J = 3.7 Hz), 123.5 (q, J = 272.7 Hz), 50.5, 38.5, 32.6, 30.6, 28.9, 27.9, 25.7, 23.1, 19.6, 14.1, 10.8.

**HRMS** (ESI) (m/z): Calcd. C_21_H_31_F_3_NO_3_^+^ [M+H]^+^ : 402.2251. Found: 402.2249.

**IR** (ν/ cm^−1^, neat): 2960, 2929, 2873, 1772, 1676, 1460, 1412, 1391, 1323, 1237, 1171, 1131, 1065, 1051, 1010, 861, 768, 700.

**3-cyclopentyl-N-isopropyl-N-((4-(trifluoromethyl)benzoyl)oxy)propenamide (1m)**

24% overall yield, colorless oil.

**^1^H NMR** (400 MHz, CDCl_3_) δ 8.24 (d, J = 8.3 Hz, 2H), 7.80 (d, J = 8.4 Hz, 2H), 4.82 (s, 1H), 2.30 (t, J = 7.6 Hz, 2H), 1.81 – 1.63 (m, 5H), 1.63 – 1.43 (m, 4H), 1.25 (d, J = 7.1 Hz, 5H), 1.12 – 1.01 (m, 2H).

**^13^C NMR** (101 MHz, CDCl_3_) δ 164.1, 135.8 (q, J = 30.6, 29.4 Hz), 130.5, 130.5, 126.1 (q, J = 3.4 Hz), 123.5 (q, J = 273.0 Hz), 50.5, 39.7, 32.6, 32.3, 30.6, 25.2, 19.6.

**HRMS** (ESI) (m/z): Calcd. C_19_H_25_F_3_NO_3_^+^ [M+H]^+^ : 372.1780. Found: 372.1781.

**IR** (ν/ cm^−1^, neat): 2947, 2868, 1771, 1675, 1513, 1456, 1411, 1392, 1323, 1237, 1170, 1129, 1065, 1051, 1010, 861, 768, 700.

**3-cyclohexyl-N-isopropyl-N-((4-(trifluoromethyl)benzoyl)oxy)propenamide (1n)**

9% overall yield, colorless oil.

**^1^H NMR** (400 MHz, CDCl_3_) δ 8.16 (d, J = 8.3 Hz, 2H), 7.72 (d, J = 8.3 Hz, 2H), 4.73 (s, 1H), 2.21 (t, J = 7.9 Hz, 2H), 1.61 – 1.51 (m, 5H), 1.50 – 1.43 (m, 2H), 1.20 – 0.99 (m, 10H), 0.83 – 0.71 (m, 2H).

**^13^C NMR** (101 MHz, CDCl_3_) δ 164.0, 135.8 (q, J = 33.0 Hz), 130.5, 130.5, 126.0 (q, J = 3.7 Hz), 123.5 (q, J = 272.9 Hz), 50.4, 37.2, 33.2, 31.8, 30.5, 26.6, 26.3, 19.5.

**HRMS** (ESI) (m/z): Calcd. C20H27F3NO3+ [M+H]+ : 386.1938. Found: 386.1936.

**IR** (ν/ cm^−1^, neat): 2923, 2852, 1771, 1675, 1449, 1412, 1391, 1322, 1236, 1170, 1129, 1066, 1051, 1009, 956, 861, 768, 700.

**N-cyclohexyl-4-methyl-N-((4-(trifluoromethyl)benzoyl)oxy)pentanamide (1o)**

16% overall yield, colorless oil.

**^1^H NMR** (400 MHz, CDCl_3_) δ 8.22 (d, J = 8.3 Hz, 2H), 7.78 (d, J = 8.3 Hz, 2H), 4.41 (s, 1H), 2.26 (s, 2H), 1.92 (d, J = 9.8 Hz, 2H), 1.79 (d, J = 9.6 Hz, 2H), 1.69 – 1.23 (m, 8H), 1.10 – 0.98 (m, 1H), 0.84 (d, J = 6.2 Hz, 6H).

**^13^C NMR** (101 MHz, CDCl_3_) δ 164.1, 135.8 (q, J = 33.2, 32.8 Hz), 130.5, 130.5, 126.0 (q, J = 3.6, 3.2 Hz), 123.5 (q, J = 273.1 Hz), 58.0, 33.3, 31.0, 30.1, 27.7, 25.6, 25.4, 22.4.

**HRMS** (ESI) (m/z): Calcd. C_20_H_27_F_3_NO_3_^+^ [M+H]^+^ : 386.1938. Found: 386.1932.

**IR** (ν/ cm^−1^, neat): 2933, 2861, 1770, 1675, 1513, 1453, 1411, 1389, 1323, 1233, 1170,1131, 1066, 1012, 965, 894, 860, 768, 701, 591, 497.

**N-(tert-butyl)-2-methyl-N-((4-(trifluoromethyl)benzoyl)oxy)benzamide (1p)**

14% overall yield, white solid.

**^1^H NMR** (400 MHz, CDCl_3_) δ 7.74 (d, J = 7.7 Hz, 2H), 7.60 (d, J = 8.3 Hz, 2H), 7.20 (d, J = 7.4 Hz, 1H), 7.10 – 6.98 (m, 3H), 2.42 (s, 3H), 1.61 (s, 9H).

**^13^C NMR** (101 MHz, CDCl_3_) δ 171.6, 164.4, 136.2, 135.4 (q, J = 32.9 Hz), 134.5, 130.2, 130.2, 129.9, 129.1, 125.9, 125.7 (q, J = 3.7 Hz), 125.3, 123.4 (q, J = 272.5 Hz), 63.2, 27.8, 19.0.

All data matched that reported in the literature^1^.

**N-(tert-butyl)-2,4-dimethyl-N-((4-(trifluoromethyl)benzoyl)oxy)benzamide (1q)**

30% overall yield, white solid.

**^1^H NMR** (400 MHz, CDCl_3_) δ 7.78 (d, J = 8.3 Hz, 2H), 7.61 (d, J = 8.4 Hz, 2H), 7.11 (d, J = 7.8 Hz, 1H), 6.89 (s, 1H), 6.82 (d, J = 7.8 Hz, 1H), 2.38 (s, 3H), 2.16 (s, 3H), 1.60 (s, 9H).

**^13^C NMR** (101 MHz, CDCl_3_) δ 171.9, 164.4, 139.0, 135.3 (q, J = 32.7 Hz), 133.4, 130.9, 130.4, 130.0, 126.0, 125.9, 125.7 (q, J = 3.9 Hz), 123.4 (q, J = 272.9 Hz), 63.1, 27.8, 21.2, 19.0.

All data matched that reported in the literature^2^.

**N-(tert-butyl)-4-methoxy-2-methyl-N-((4-(trifluoromethyl)benzoyl)oxy)benzamide (1r)**

30% overall yield, colorless oil.

**^1^H NMR** (400 MHz, CDCl_3_) δ 7.85 (d, J = 8.3 Hz, 2H), 7.64 (d, J = 8.4 Hz, 2H), 7.20 (d, J = 8.4 Hz, 1H), 6.62 (d, J = 2.7 Hz, 1H), 6.56 (dd, J = 8.4, 2.7 Hz, 1H), 3.68 (s, 3H), 2.42 (s, 3H), 1.60 (s, 9H).

**^13^C NMR** (101 MHz, CDCl_3_) δ 171.6, 164.4, 160.0, 135.3 (q, J = 33.0 Hz), 130.3, 130.0, 128.7, 127.8, 125.7 (q, J = 3.9 Hz), 123.4 (q, J = 272.9 Hz), 115.7, 110.5, 63.1, 55.2, 27.8, 19.4.

All data matched that reported in the literature^2^.

**N-(tert-butyl)-4-chloro-2-methyl-N-((4-(trifluoromethyl)benzoyl)oxy)benzamide (1s)**

14% overall yield, colorless oil.

**^1^H NMR** (400 MHz, CDCl_3_) δ 7.81 (d, J = 8.3 Hz, 2H), 7.64 (d, J = 8.4 Hz, 2H), 7.16 (d, J = 8.2 Hz, 1H), 7.08 (d, J = 2.3 Hz, 1H), 7.01 (dd, J = 8.2, 2.3 Hz, 1H), 2.39 (s, 3H), 1.59 (s, 9H).

**^13^C NMR** (101 MHz, CDCl_3_) δ 170.5, 164.3, 137.0, 135.6 (q, J = 33.0 Hz), 134.8, 134.6, 130.2, 130.0, 129.9, 127.4, 125.9 (q, J = 3.7 Hz), 125.5, 123.4 (q, J = 272.9 Hz), 63.5, 27.7, 18.9.

All data matched that reported in the literature^2^.

**4-bromo-N-(tert-butyl)-2-methyl-N-((4-(trifluoromethyl)benzoyl)oxy)benzamide (1t)**

10% overall yield, colorless oil.

**^1^H NMR** (400 MHz, CDCl_3_) δ 7.82 (d, J = 8.3 Hz, 2H), 7.65 (d, J = 8.4 Hz, 2H), 7.26 (d, J = 2.1 Hz, 1H), 7.18 (dd, J = 8.2, 2.1 Hz, 1H), 7.10 (d, J = 8.2 Hz, 1H), 2.40 (s, 3H), 1.60 (s, 9H).

**^13^C NMR** (101 MHz, CDCl_3_) δ 170.5, 164.3, 137.2, 135.6 (q, J = 33.0 Hz), 135.1, 133.1, 130.0, 129.9, 128.4, 127.6, 125.9 (q, J = 3.9 Hz), 123.4 (q, J = 273.1 Hz), 123.1, 63.5, 27.7, 18.9.

**HRMS** (ESI) (m/z): Calcd. C_20_H_20_BrF_3_NO_3_^+^ [M+H]^+^:458.0573. Found: 458.0574.

**IR** (ν/ cm^−1^, neat): 2981, 1773, 1655, 1589, 1516, 1481, 1457, 1412, 1366, 1322, 1256, 1232, 1194, 1127, 1113, 1064, 1041, 1007, 883, 861, 823, 777, 753, 698, 677.

**N-(tert-butyl)-2-ethyl-N-((4-(trifluoromethyl)benzoyl)oxy)benzamide (1u)**

13% overall yield, white solid.

**^1^H NMR** (400 MHz, CDCl_3_) δ 7.71 (d, J = 8.0 Hz, 2H), 7.59 (d, J = 8.1 Hz, 2H), 7.20 (d, J = 7.6 Hz, 1H), 7.18 – 7.09 (m, 2H), 7.07 – 6.97 (m, 1H), 2.86 – 2.69 (m, 2H), 1.62 (s, 9H), 1.29 (t, J = 7.5 Hz, 3H).

**^13^C NMR** (101 MHz, CDCl_3_) δ 174.4, 164.5, 141.6, 135.7 (q, J = 33.0 Hz), 130.4, 130.1, 128.5, 128.3, 127.6, 126.0 (q, J = 3.7 Hz), 125.8, 123.4 (q, J = 272.9 Hz), 62.9, 35.0, 33.4, 27.5, 25.7.

All data matched that reported in the literature^3^.

**N-(tert-butyl)-4-phenyl-N-((4-(trifluoromethyl)benzoyl)oxy)butanamide (1v)**

74% overall yield, colorless oil.

**^1^H NMR** (400 MHz, CDCl_3_) δ 8.15 (d, J = 8.1 Hz, 2H), 7.78 (d, J = 8.2 Hz, 2H), 7.23 – 7.14 (m, 2H), 7.14 – 7.06 (m, 3H), 2.69 – 2.51 (m, 2H), 2.29 (dt, J = 15.4, 7.5 Hz, 1H), 2.11 (dt, J = 15.9, 7.2 Hz, 1H), 1.99 – 1.84 (m, 2H), 1.49 (s, 9H).

**^13^C NMR** (101 MHz, CDCl_3_) δ 174.4, 164.5, 141.6, 135.7 (q, J = 33.0 Hz), 130.4, 130.1, 128.4, 128.2, 127.6, 126.0 (q, J = 3.7 Hz), 125.8, 123.4 (q, J = 272.9 Hz), 62.8, 35.0, 33.4, 27.5, 25.7.

**HRMS** (ESI) (m/z): Calcd. C_22_H_25_F_3_NO_3_^+^ [M+H]^+^ : 408.1781. Found: 408.1782.

**IR** (ν/ cm^−1^, neat): 3026, 2983, 2935, 1769, 1673, 1455, 1411, 1365, 1323, 1251, 1234, 1170, 1131, 1065, 1045, 1011, 770, 746, 699.

All data matched that reported in the literature^3^.

**N-(tert-butyl)-4-(p-tolyl)-N-((4-(trifluoromethyl)benzoyl)oxy)butanamide (1w)**

3% overall yield, colorless oil.

**^1^H NMR** (400 MHz, CDCl_3_) δ 7.25 (d, J = 7.7 Hz, 2H), 7.15 (d, J = 7.8 Hz, 2H), 5.40 (s, 1H), 4.73 (dd, J = 7.5, 4.7 Hz, 1H), 3.74 (s, 1H), 2.34 (s, 3H), 2.24 (t, J = 6.7 Hz, 2H), 2.09 – 1.96 (m, 2H), 1.34 (s, 9H).

**^13^C NMR** (101 MHz, CDCl_3_) δ 174.6, 164.6, 138.6, 135.9 (q, J = 33.6 Hz), 135.3, 130.5, 130.2, 129.1, 128.5, 126.1 (q, J = 3.7 Hz), 123.5 (q, J = 272.7 Hz).

All data matched that reported in the literature^3^.

**(S)-N-(tert-butyl)-2-methoxy-4-methyl-N-((4-(trifluoromethyl)benzoyl)oxy)pentanamide (1x)**

(S)-2-hydroxy-4-methylpentanoic acid (2.64 g, 20 mmol) was dissolved in 40 mL DMF, after cooling to 0°C, sodium hydride (1.2 g, 60 % in mineral oil, 30mmol) was added in portion and stirring is continued for 1 hour. Then methyliodide (1.9 mL, 30 mmol) was added dropwise and the mixture is stirred for further 12 hours, while warming to room temperature. After cooling to 0°C, 10 ml of a 10 M solution of sodium hydroxide in water are added and the mixture is stirred for 2 hours. Then concentrated hydrochloric acid was added until pH of 1 was reached, water was added to the reaction mixture and the aqueous layer was extracted with EtOAc (3x). If phase separation was slow, brine was added. The organic phases were combined and washed with saturated brine, the organic phase was dried over anhydrous sodium sulfate and filtered, and then concentrated under reduced pressure. (S)-2-methoxy-4-methylpentanoic acid was obtained in 15% yield, yellow oil. The crude product is used directly in the next step.

Prepared according to general procedure giving **1x** in 10% overall yield, colorless oil.

**^1^H NMR** (400 MHz, CDCl_3_) (Rotamer mixture, ratio: 1:0.79) δ 8.19 (dd, J = 17.8, 8.3 Hz, 2H), 7.79 (t, J = 9.6 Hz, 2H), 3.85 – 3.58 (m, 1H), 3.24 (d, J = 8.9 Hz, 3H), 1.84 – 1.60 (m, 2H), 1.60 – 1.50 (m, 1H), 1.49 (s, 9H), 0.86 (d, J = 6.6 Hz, 3H), 0.81 – 0.57 (m, 3H).

**^13^C NMR** (101 MHz, CDCl_3_) (Rotamer mixture, ratio: 1:0.79) δ 174.1, 173.7, 164.6, 164.6, 136.1 (q, J = 33.0 Hz), 130.9, 130.5, 130.3, 129.9, 129.8, 126.2 (q, J = 3.7 Hz), 126.1, 126.0, 123.4 (q, J = 273.2 Hz), 82.7, 77.9, 63.7, 58.4, 57.3, 40.8, 40.7, 27.5, 27.2, 26.7, 24.8, 24.4, 23.5, 23.2, 22.0, 21.6.

**HRMS** (ESI) (m/z): Calcd. C_19_H_27_F_3_NO_4_^+^ [M+H]^+^ : 390.1887. Found: 390.1882.

**IR** (ν/ cm^−1^, neat): 2958, 1773, 1682, 1466, 1412, 1395, 1366, 1323, 1235, 1172, 1130, 1111, 1066, 1040, 1009, 861, 804, 770, 697, 651.

**(S)-1-(tert-butyl((4-(trifluoromethyl)benzoyl)oxy)amino)-4-methyl-1-oxopentan-2-yl acetate (1y)**

**1y** was prepared according to literature procedure^1^.

8% overall yield, white solid.

**^1^H NMR** (400 MHz, CDCl_3_) (Rotamer mixture, ratio: 1:0.54) δ 8.27 (t, J = 9.5 Hz, 3H), 7.82 (d, J = 8.3 Hz, 3H), 5.10 (dd, J = 10.9, 2.9 Hz, 1H), 5.03 (dd, J = 10.3, 3.2 Hz, 1H), 2.10 (s, 4H), 1.80 (tdd, J = 19.2, 12.2, 7.2 Hz, 2H), 1.52 (s, 17H), 0.92 (d, J = 6.5 Hz, 2H), 0.86 (d, J = 6.6 Hz, 3H), 0.68 (d, J = 6.5 Hz, 2H), 0.59 (d, J = 6.6 Hz, 3H).

**^13^C NMR** (101 MHz, CDCl_3_) (Rotamer mixture, ratio: 1:0.54) δ 173.2, 170.8, 170.3, 164.5, 163.7, 135.9 (q, J = 32.6 Hz), 130.6, 130.5, 130.1, 129.8, 126.2 (q, J = 3.7 Hz), 123.4 (q, J = 273.3 Hz), 70.6, 70.1, 64.1, 63.8, 39.6, 39.2, 27.3, 27.1, 24.6, 23.3, 23.2, 21.5, 21.2, 20.6.

All data matched that reported in the literature^1^.

**(S)-N-(tert-butyl)-2-(1,3-dioxoisoindolin-2-yl)-4-methyl-N-((4-(trifluoromethyl)benzoyl)oxy)pentanamide (1z)**

Prepared from commercially available phthaloyl-L-leucine.

8% overall yield, white solid.

**^1^H NMR** (400 MHz, CDCl_3_) (Rotamer mixture, ratio: 1:0.84) δ 8.14 (d, J = 8.4 Hz, 2H), 8.02 (d, J = 8.3 Hz, 2H), 7.83 – 7.64 (m, 11H), 4.93 (dd, J = 11.1, 3.9 Hz, 1H), 4.83 (dd, J = 9.9, 4.8 Hz, 1H), 2.58 – 2.44 (m, 1H), 2.22 – 2.10 (m, 1H), 1.94 – 1.84 (m, 1H), 1.83 – 1.73 (m, 1H), 1.47 (d, J = 15.5 Hz, 19H), 0.88 (dd, J = 10.3, 6.5 Hz, 6H), 0.79 (d, J = 6.4 Hz, 3H), 0.70 (d, J = 6.3 Hz, 2H).

**^13^C NMR** (101 MHz, CDCl_3_) (Rotamer mixture, ratio: 1:0.84) δ 170.3, 169.7, 167.8, 167.3, 164.4, 164.3, 136.1, 135.8, 135.5, 135.1, 134.2, 134.0, 131.8, 131.7, 130.5, 130.4, 130.0, 129.8, 127.4, 125.9, 125.8, 124.7, 123.4, 123.3, 122.0, 119.3, 64.3, 64.1, 51.3, 50.7, 37.5, 36.6, 27.5, 27.3, 25.0, 24.7, 23.2, 23.0, 21.5, 21.2.

All data matched that reported in the literature^1^.

**(3R,5R,8R,9S,10S,13R,14S,17R)-17-((S)-5-(tert-butyl((4-(trifluoromethyl)benzoyl)oxy)amino)-5-oxopentan-2-yl)-10,13-dimethylhexadecahydro-1H-cyclopenta[a]phenanthren-3-yl acetate (1aa)**

**1aa** was prepared according to literature procedure using lithocholic acid as starting material^4^.

9% overall yield, white solid.

**^1^H NMR** (400 MHz, CDCl_3_) δ 8.24 (d, J = 8.1 Hz, 2H), 7.82 (d, J = 8.2 Hz, 2H), 4.80 – 4.60 (m, 1H), 2.36 – 2.09 (m, 2H), 2.03 (s, 3H), 1.95 – 1.73 (m, 6H), 1.71 – 1.63 (m, 1H), 1.49 (s, 11H), 1.45 – 1.19 (m, 11H), 1.14 – 0.96 (m, 6H), 0.91 (s, 3H), 0.78 (d, J = 6.1 Hz, 3H), 0.58 (s, 3H).

**^13^C NMR** (101 MHz, CDCl_3_) δ 171.9, 163.7, 147.2, 145.7, 135.6 (q, J = 32.9 Hz), 134.7, 133.4, 130.9, 130.1, 128.1, 128.0, 127.0, 125.8 (q, J = 3.6 Hz), 124.2, 123.9, 123.3 (q, J = 273.2 Hz), 59.7, 45.6, 38.8, 38.4, 37.7, 37.0, 33.4, 30.2, 25.7, 24.0, 24.0, 19.3.

All data matched that reported in the literature^4^.

**N-(((1R,4aS,10aR)-7-isopropyl-1,4a-dimethyl-1,2,3,4,4a,9,10,10a-octahydrophenanthren-1-yl)methyl)-N-((4-(trifluoromethyl)benzoyl)oxy)benzamide (1ab)**

**1ab** was prepared according to literature procedure using dehydroabietylamine as starting material^4^.

30% overall yield, white solid.

**^1^H NMR** (400 MHz, CDCl_3_) δ 7.94 (d, J = 8.3 Hz, 2H), 7.65 (d, J = 8.6 Hz, 2H), 7.63 – 7.57 (m, 2H), 7.34 – 7.25 (m, 3H), 7.20 (d, J = 8.3 Hz, 1H), 7.01 (dd, J = 8.2, 2.2 Hz, 1H), 6.85 (d, J = 2.2 Hz, 1H), 2.90 – 2.70 (m, 3H), 2.35 (d, J = 3.1 Hz, 1H), 1.97 – 1.57 (m, 8H), 1.48 (td, J = 12.9, 4.0 Hz, 1H), 1.31 – 1.17 (m, 10H), 1.06 (s, 3H).

**^13^C NMR** (101 MHz, CDCl_3_) δ 171.9, 163.7, 147.2, 145.7, 135.6 (q, J = 32.9 Hz), 134.7, 133.4, 130.9, 130.1, 128.1, 128.0, 127.0, 125.8 (q, J = 3.6 Hz), 124.2, 123.9, 123.3 (q, J = 273.2 Hz), 59.7, 45.6, 38.8, 38.4, 37.7, 37.0, 33.4, 30.2, 25.7, 24.0, 24.0, 19.3.

All data matched that reported in the literature^4^.

## Reaction optimization

The optimization of the reaction was carried out using N-(tert-butyl)-4-methyl-N-((4-(trifluoromethyl)benzoyl)oxy)pentanamide **(1a)** as the test system. In a glovebox under a nitrogen atmosphere, a 10 mL Schlenk tube with a stir bar was sequentially added B_2_cat_2_, **1a** (0.2 mmol), photocatalyst, 2 mL solvent, and additive. The capped Schlenk tube was removed from the glovebox, and the reaction mixture was irradiated by Kessil 40 W blue LED at a 3 cm distance for a specific time, with a fan for cooling. After cooling to room temperature, pinacol (4.0 equiv.) and 1.0 mL triethylamine were added, and the mixture was stirred at room temperature for 1 hour. After that, then n-decane (~25 mg) was added as internal standard, the reaction mixture was washed with H_2_O (5 mL), extracted using ethyl acetate (5 mL x 2), and the yield was measured by GC-FID. The results are shown in **Supplementary Table 1**.

**Supplementary Table 1** Optimization of reaction conditions.

| entry^a^ | B_2_cat_2_ (equiv.) | PC (equiv.) | light source | additive (equiv.) | yield^b^ (%) |
| --- | --- | --- | --- | --- | --- |
| 1 | 3.0 | Ir(p-CF_3_ppy)_3_ (2%) | 456 nm | Et_3_N (2.0) | 45 |
| 2 | 3.0 | Ir(p-CF_3_ppy)_3_ (2%) | 456 nm | DABCO (2.0) | 15 |
| 3 | 3.0 | Ir(p-CF_3_ppy)_3_ (2%) | 456 nm | DIPEA (2.0) | 47 |
| 4 | 3.0 | Ir(p-CF_3_ppy)_3_ (2%) | 456 nm | none | trace |
| 5^c^ | 3.0 | Ir(p-CF_3_ppy)_3_ (2%) | 456 nm | DIPEA (2.0) | 49 |
| 6 | 3.0 | Ir(ppy)_3_ (2%) | 456 nm | DIPEA (2.0) | 30 |
| 7 | 3.0 | [Ru(bpy)_3_]Cl_2_ (2%) | 456nm | DIPEA (2.0) | trace |
| 8 | 3.0 | 4CzIPN (2%) | 456 nm | DIPEA (2.0) | trace |
| 9 | 3.0 | Eosin Y (2%) | 456 nm | DIPEA (2.0) | 47 |
| 10 | 3.0 | Eosin Y (2%) | 467 nm | DIPEA (2.0) | 73 |
| 11 | 3.0 | Eosin Y (5%) | 467 nm | DIPEA (2.0) | 77 |
| **12** | **2.5** | **Eosin Y (5%)** | **467 nm** | **DIPEA (2.0)** | **81 (73)** |
| 13 | 4.0 | Eosin Y (5%) | 467 nm | DIPEA (2.0) | 40 |
| 14 | 2.5 | Eosin Y (5%) | 467 nm | DIPEA (1.5) | 54 |
| 15 | 2.5 | none | 467 nm | DIPEA (2.0) | trace |
| 16 | 2.5 | Eosin Y (5%) | none | DIPEA (2.0) | trace |
| 17 | 2.5 | Eosin Y (5%) | 467 nm | none | trace |
| 18^d^ | 2.5 | Eosin Y (5%) | none | DIPEA (2.0) | trace |
| 19^e^ | 2.5 | Eosin Y (5%) | 467 nm | DIPEA (2.0) | trace |
| 20^f^ | 2.5 | Eosin Y (5%) | 467 nm | DIPEA (2.0) | 76 |

a: Reaction Condition: **1a** (0.2 mmol), B_2_cat_2_ (Bis(catecholato)diboron), photocatalyst, DMA (Dimethylacetamide) (2 mL), additive, then irradiated by Kessil 40 W blue LED for certain time. After the reaction, pinacol (4.0 equiv.) and Et_3_N (1 mL) were added, stirred for 1h. Decane is used as an internal standard.

b: GC-fid yield, the yield in bracket is isolated yield.

c: The reaction time was 30 h.

d : 50 °C for 12 h, in the dark.

e : Turned off the light after 30 min.

f : The reaction was set up in air.

## Experimental procedures and characterization data

### 4.1 General procedure

In a glovebox under a nitrogen atmosphere, sequentially added B_2_cat_2_ (0.75 mmol, 1.5 equiv., 178 mg), the substrate (0.3 mmol, 1.0 equiv.), Eosin Y (5%, 0.015 mmol, 10.5 mg) and 3 mL DMA to a 10mL Schlenk tube with a stir bar, followed by DIPEA (0.6 mmol, 2.0 equiv., 105 μL). The capped Schlenk tube was removed from the glovebox, and the reaction mixture was irradiated by 467nm Kessil 40W LED at a 3 cm distance for 12 hours, with a fan for cooling. As the catechol boronate esters are sensitive to hydrolysis, RBcat was transformed to RBpin for isolation. After cooling to room temperature, pinacol (142 mg, 4.0 equiv.) and 1.0 mL triethylamine were added, and the mixture was stirred at room temperature for 1 hour. Water was added to the reaction mixture, the aqueous layer was extracted with EtOAc (20 mL x 2). If phase separation was slow, brine was added. The organic phases were combined and washed with 50 mL of saturated brine, the organic phase was dried over anhydrous sodium sulfate and filtered, then concentrated under reduced pressure, purified by column chromatography to give the product. The product was monitored by thin-layer chromatography with phosphomolybdic acid (PMA) stain.


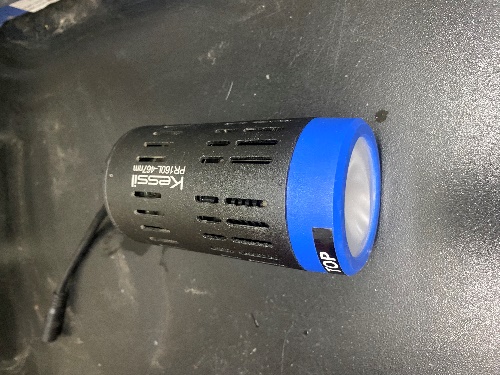

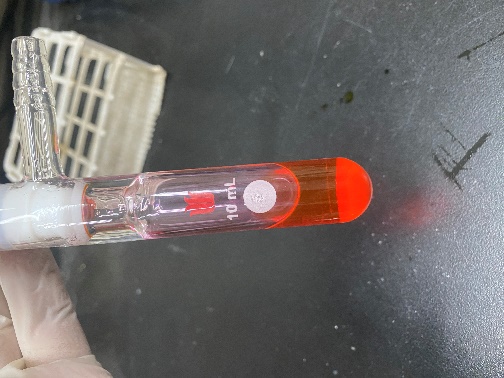

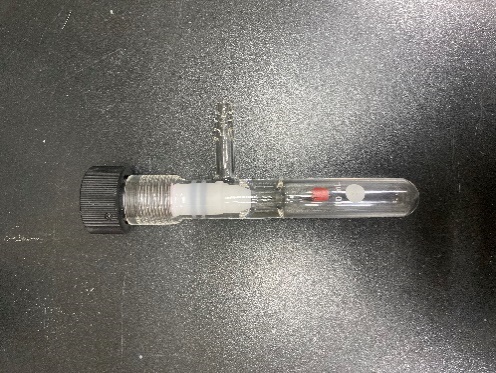


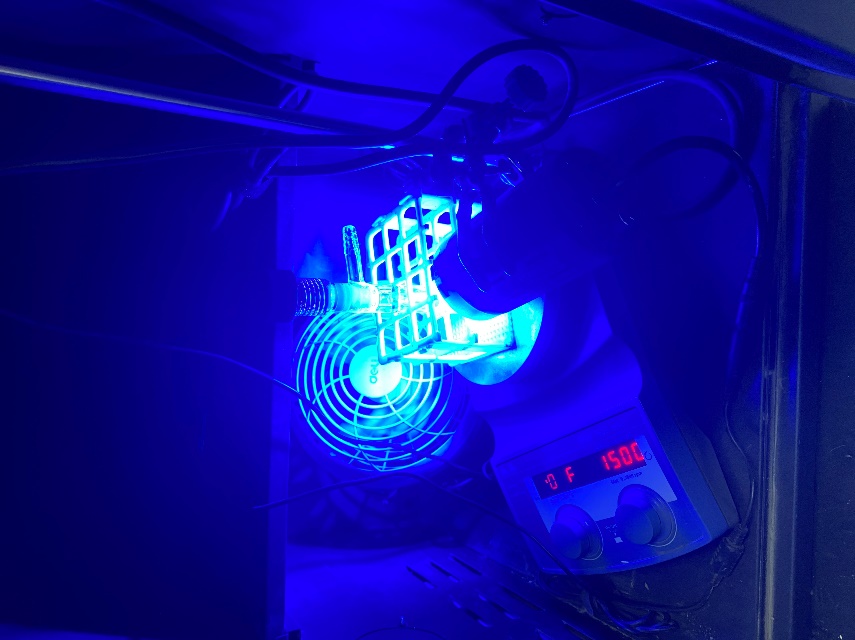


**Supplementary Figure 1.** Standard reaction setup. *(left)*: SYNTHWARE 10 mL Schlenk tube*. (middle):* Kessil PR160L-467 nm LED light. *(right):* The reaction mixture before irradiation. *(below):* The Standard reaction setup, with a fan for cooling.

### 4.2 Characterization of products

**N-(tert-butyl)-4-methyl-4-(4,4,5,5-tetramethyl-1,3,2-dioxaborolan-2-yl)pentanamide (2a)**

65.1 mg, 73% isolated yield, white solid. R_f_ = 0.3 (PE/ EA = 5:1), using PE/ EA = 10:1 as eluent (1% Et_3_N).

**^1^H NMR** (400 MHz, CDCl_3_) δ 5.33 (s, 1H), 2.11 – 2.00 (m, 2H), 1.61 – 1.52 (m, 2H), 1.34 (s, 9H), 1.23 (s, 12H), 0.93 (s, 6H).

**^13^C NMR** (101 MHz, CDCl_3_) δ 173.2, 83.1, 50.9, 36.7, 35.1, 28.8, 24.7, 24.6. The signal of the α-B-carbon was not observed.

**^11^B NMR** (160 MHz, CDCl_3_) δ 34.62.

**HRMS** (ESI) (m/z): Calcd. C_16_H_33_BNO_3_^+^ [M+H]^+^: 298.2548. Found: 298.2540. Calcd. C_16_H_32_BNNaO_3_^+^ [M+Na]^+^: 320.2367. Found: 320.2363.

**IR** (ν/ cm^−1^, neat): 3281, 2971, 2925, 2864, 1642, 1557, 1477, 1452, 1365, 1306, 1294, 1130, 969, 854, 697.

**N-(tert-butyl)-4-methyl-4-(4,4,5,5-tetramethyl-1,3,2-dioxaborolan-2-yl)hexanamide (2b)**

62.6 mg, 67% isolated yield, white solid. R_f_ = 0.3 (PE/ EA = 5:1), using PE/ EA = 10:1 as eluent (1% Et_3_N).

**^1^H NMR** (400 MHz, CDCl_3_) δ 5.33 (s, 1H), 2.17 (s, 1H), 2.12 – 1.99 (m, 2H), 1.75 – 1.64 (m, 1H), 1.54 – 1.39 (m, 2H), 1.34 (s, 9H), 1.24 (s, 12H), 0.93 – 0.79 (m, 6H).

**^13^C NMR** (101 MHz, CDCl_3_) δ 173.3, 83.1, 50.9, 34.5, 34.4, 31.3, 28.8, 24.8, 24.8, 20.8, 10.0. The signal of the α-B-carbon was not observed.

**^11^B NMR** (160 MHz, CDCl_3_) δ 34.67.

**HRMS** (ESI) (m/z): Calcd. C_17_H_35_BNO_3_^+^ [M+H]^+^: 312.2705. Found: 312.2700.

**IR** (ν/ cm^−1^, neat): 3281, 3085, 2972, 2923, 1642, 1556, 1457, 1371, 1302, 1258, 1136, 1110, 1084, 964, 853, 691.

**N-(tert-butyl)-4-ethyl-4-(4,4,5,5-tetramethyl-1,3,2-dioxaborolan-2-yl)octanamide (2c)**

87.0 mg, 82% isolated yield, colorless oil. R_f_ = 0.3 (PE/ EA = 5:1), using PE/ EA = 10:1 as eluent (1% Et_3_N.

**^1^H NMR** (400 MHz, CDCl_3_) δ 5.4 (d, J = 8.9 Hz, 1H), 2.0 – 1.9 (m, 2H), 1.6 – 1.5 (m, 1H), 1.5 – 1.4 (m, 2H), 1.3 (s, 1H), 1.3 (s, 11H), 1.2 (s, 15H), 0.9 – 0.7 (m, 7H).

**^13^C NMR** (101 MHz, CDCl_3_) δ 172.5, 82.0, 49.9, 32.8, 32.6, 28.8, 27.8, 26.0, 25.7, 23.9, 22.6, 13.1, 8.1, 0.0.

**^11^B NMR** (160 MHz, CDCl_3_) δ 34.79.

**HRMS** (ESI) (m/z): Calcd. C_20_H_41_BNO_3_^+^ [M+H]^+^: 354.3174. Found: 354.3170.

**IR** (ν/ cm^−1^, neat): 3306, 2927, 2873, 1643, 1545, 1454, 1388, 1372, 1306, 1252, 1224, 1166, 1137, 1067, 969, 855, 801, 689.

**N-(tert-butyl)-4-methyl-4-(4,4,5,5-tetramethyl-1,3,2-dioxaborolan-2-yl)nonanamide (2d)**

55.2 mg, 52% isolated yield, white solid. R_f_ = 0.4 (PE/ EA = 3:1), using PE/ EA = 10:1 as eluent (1% Et_3_N).

**^1^H NMR** (400 MHz, CDCl_3_) δ 5.28 (s, 1H), 2.07 – 1.84 (m, 2H), 1.52 (dddd, J = 83.2, 13.3, 11.3, 5.6 Hz, 2H), 1.21 (d, J = 43.9 Hz, 29H), 0.89 – 0.73 (m, 6H).

**^13^C NMR** (101 MHz, CDCl_3_) δ 173.3, 83.0, 50.9, 39.0, 34.7, 34.4, 32.7, 28.8, 25.3, 24.8, 22.6, 21.3, 14.0. The signal of the α-B-carbon was not observed.

**HRMS** (ESI) (m/z): Calcd. C_20_H_41_BNO_3_^+^ [M+H]^+^: 354.3174. Found: 354.3168.

**IR** (ν/ cm^−1^, neat): 3288, 2962, 2922, 2854, 1642, 1556, 1507, 1456, 1387, 1369, 1344, 1306, 1286, 1259, 1228, 1210, 1163, 1138, 1061, 1023, 1002, 965, 856, 833, 771, 701, 639.

**N-(tert-butyl)-4-(4,4,5,5-tetramethyl-1,3,2-dioxaborolan-2-yl)dodecanamide (2e)**

57.5 mg, 51% isolated yield, white solid. R_f_ = 0.3 (PE/ EA = 3:1), using PE/ EA = 3:1 as eluent (1% Et_3_N).

**^1^H NMR** (400 MHz, CDCl_3_) δ 5.22 (s, 1H), 2.12 – 2.03 (m, 2H), 1.77 – 1.52 (m, 3H), 1.49 (s, 2H), 1.34 (s, 12H), 1.27 (d, J = 13.8 Hz, 20H), 0.88 (td, J = 6.9, 2.0 Hz, 3H).

**^13^C NMR** (101 MHz, CDCl_3_) δ 172.8, 83.0, 51.0, 37.6, 31.9, 31.3, 29.9, 29.5, 29.3, 29.1, 28.8, 27.7, 24.9, 22.7, 14.1. The signal of the α-B-carbon was not observed.

**HRMS** (ESI) (m/z): Calcd. C_22_H_45_BNO_3_^+^ [M+H]+: 382.3487. Found: 382.3487.

**IR** (ν/ cm^−1^, neat): 3304, 3073, 2961, 2923, 2854, 1644, 1546, 1454, 1386, 1368, 1314, 1262, 1225, 1143, 1106, 968, 857, 804, 686, 579.

**N-(tert-butyl)-3-(1-(4,4,5,5-tetramethyl-1,3,2-dioxaborolan-2-yl)cyclopentyl)propanamide (2f)**

80.3 mg, 83% isolated yield, white solid. R_f_ = 0.3 (PE/ EA = 5:1), using PE/ EA = 10:1 as eluent (1% Et_3_N).

**^1^H NMR** (400 MHz, CDCl_3_) δ 5.43 (s, 1H), 2.02 – 1.89 (m, 2H), 1.75 – 1.61 (m, 2H), 1.60 – 1.37 (m, 6H), 1.24 (s, 9H), 1.13 (s, 14H).

**^13^C NMR** (101 MHz, CDCl_3_) δ 173.1, 82.9, 50.8, 36.3, 35.1, 34.4, 28.7, 25.2, 24.6. The signal of the α-B-carbon was not observed.

**HRMS** (ESI) (m/z): Calcd. C_18_H_35_BNO_3_^+^ [M+H]^+^: 324.2705. Found: 324.2708.

**IR** (ν/ cm^−1^, neat): 3301, 2920, 2853, 1642, 1549, 1453, 1386, 1372, 1303, 1282, 1256, 1226, 1142, 1106, 1043, 971, 857, 808, 700, 634.

**N-(tert-butyl)-3-(1-(4,4,5,5-tetramethyl-1,3,2-dioxaborolan-2-yl)cyclohexyl)propanamide (2g)**

58.7 mg, 58% isolated yield, white solid. R_f_ = 0.3 (PE/ EA = 5:1), using PE/ EA = 10:1 as eluent (1% Et_3_N).

**^1^H NMR** (400 MHz, CDCl_3_) δ 5.25 (s, 1H), 2.10 – 2.01 (m, 2H), 1.84 (d, J = 13.0 Hz, 2H), 1.71 (s, 2H), 1.67 – 1.52 (m, 5H), 1.33 (s, 9H), 1.25 (s, 12H), 1.19 – 1.08 (m, 1H), 0.93 (td, J = 12.7, 3.4 Hz, 2H).

**^13^C NMR** (101 MHz, CDCl_3_) δ 173.1, 83.1, 51.0, 36.3, 34.9, 34.3, 28.8, 26.6, 25.0, 24.9. The signal of the α-B-carbon was not observed.

**HRMS** (ESI) (m/z): Calcd. C_19_H_37_BNO_3_^+^ [M+H]^+^: 338.2861. Found: 338.2860.

**IR** (ν/ cm^−1^, neat): 3289, 3079, 2973, 2921, 2852, 1641, 1552, 1452, 1390, 1361, 1303, 1229, 1134, 969, 860, 682.

**(±) (1S,3R)-N-(tert-butyl)-3-(4,4,5,5-tetramethyl-1,3,2-dioxaborolan-2-yl)cycloheptane-1-carboxamide (2h)**

43% yield, white solid. R_f_ = 0.35 (PE/ EA = 5:1), using PE/ EA = 15:1 as eluent (1% Et_3_N).

**^1^H NMR** (400 MHz, CDCl_3_) δ 5.19 (s, 1H), 2.11 – 2.01 (m, 1H), 1.91 – 1.39 (m, 12H), 1.33 (s, 9H), 1.23 (s, 12H).

**^13^C NMR** (101 MHz, CDCl_3_) δ 176.9, 83.2, 50.9, 50.4, 33.3, 32.5, 29.6, 29.0, 28.2, 26.1, 24.9. The signal of the α-B-carbon was not observed.

**HRMS** (ESI) (m/z): Calcd. C_18_H_35_BNO_3_^+^ [M+H]^+^: 324.2705. Found: 324.2702.

**IR** (ν/ cm^−1^, neat): 3310, 2972, 2902, 2848, 1637, 1542, 1450, 1360, 1338, 1313, 1276, 1224, 1144, 1101, 1067, 968, 870, 850, 687.

**(1R,4R,7R)-N-(tert-butyl)-7-(4,4,5,5-tetramethyl-1,3,2-dioxaborolan-2-yl)bicyclo[2.2.1]heptane-2-carboxamide (2i)**

62.6 mg, 65% isolated yield, white solid. R_f_ = 0.4 (PE/ EA = 5:1), using PE/ EA = 15:1 as eluent (1% Et_3_N).

**^1^H NMR** (400 MHz, CDCl_3_) δ 5.25 (s, 1H), 2.62 – 2.49 (m, 1H), 2.42 (d, J = 4.2 Hz, 1H), 2.24 (t, J = 4.3 Hz, 1H), 1.69 – 1.52 (m, 3H), 1.51 – 1.43 (m, 1H), 1.34 (s, 11H), 1.21 (s, 12H).

**^13^C NMR** (101 MHz, CDCl_3_) δ 173.3, 82.9, 51.0, 49.0, 43.1, 40.4, 37.3, 31.8, 30.8, 29.0, 24.7, 24.6. The signal of the α-B-carbon was not observed.

**HRMS** (ESI) (m/z): Calcd. C_18_H_33_BNO_3_^+^ [M+H]^+^: 322.2548. Found: 322.2550.

**IR** (ν/ cm^−1^, neat): 3376, 3326, 2974, 2872, 1671, 1648, 1525, 1452, 1369, 1308, 1287, 1249, 1214, 1173, 1124, 1068, 959, 849, 743, 706, 674.

**N-(tert-butyl)-2-((1S,2S,3R,5R,7S)-2-(4,4,5,5-tetramethyl-1,3,2-dioxaborolan-2-yl)adamantan-1-yl)acetamide** **(2j)**

72.0 mg, 64% isolated yield, white solid. R_f_ = 0.35 (PE/ EA = 5:1), using PE/ EA = 15:1 as eluent (1% Et_3_N).

**^1^H NMR** (400 MHz, CDCl_3_) δ 6.41 (s, 1H), 2.28 (d, J = 12.5 Hz, 1H), 2.08 (q, J = 3.4 Hz, 1H), 2.00 – 1.91 (m, 3H), 1.86 – 1.56 (m, 10H), 1.46 – 1.40 (m, 1H), 1.34 (s, 9H), 1.27 (d, J = 3.4 Hz, 12H).

**^13^C NMR** (101 MHz, CDCl_3_) δ 170.7, 83.1, 51.6, 50.8, 43.8, 42.8, 38.6, 36.9, 34.9, 34.2, 30.4, 28.9, 28.7, 28.6, 25.2, 24.6. The signal of the α-B-carbon was not observed.

**HRMS** (ESI) (m/z): Calcd. C_22_H_39_BNO_3_^+^ [M+H]^+^: 376.3018. Found: 376.3016.

**IR** (ν/ cm^−1^, neat): 3310, 2972, 2902, 2848, 1637, 1542, 1450, 1360, 1338, 1313, 1276, 1224, 1144, 1101, 1067, 968, 870, 850, 687.

**N-isopropyl-4-methyl-4-(4,4,5,5-tetramethyl-1,3,2-dioxaborolan-2-yl)pentanamide (2k)**

57.9 mg, 55% yield (calculated by ^1^H NMR), white solid. R_f_ = 0.4 (PE/ EA = 1:1), using PE/ EA = 3:1 – 1:1 as eluent (1% Et_3_N).

**^1^H NMR** (400 MHz, DMSO-*d6*) (mixture) δ 7.58 (d, J = 7.7 Hz, 1H), 3.85 – 3.72 (m, 1H), 1.98 – 1.91 (m, 2H), 1.42 – 1.35 (m, 2H), 1.17 (s, 12H), 1.01 (d, J = 6.6 Hz, 6H), 0.84 (s, 6H).Although we tried lots of methods, it’s hard to separate the product and its hydrogenation side product, the spectra showed a mixture of them, the yield of product was calculated according to the ratio of the area of the signals δ 2.05 – 1.98 and δ 1.98 – 1.91(CH_2_ connected to carbonyl group), side product and product mole ratio = 0.87 / 2.07 = 0.42:1, product yield = 57.9 / (0.42*157.3+1*283.2) / 0.3 = 55%.

**^13^C NMR** (101 MHz, DMSO-*d6*) (mixture) δ 171.8, 171.7, 83.2, 36.6, 34.8, 34.0, 32.8, 27.7, 25.1, 25.0, 22.9, 22.8. The signal of the α-B-carbon was not observed.

**HRMS** (ESI) (m/z): Calcd. C_15_H_31_BNO_3_^+^ [M+H]^+^: 284.2392. Found: 284.2391.

**IR** (ν/ cm^−1^, neat): 3240, 3070, 2972, 2936, 2864, 1630, 1551, 1473, 1365, 1308,1276, 1251, 1201, 1166, 1134, 1049, 1017, 966, 852, 804, 756, 718, 691, 627, 581.

**4-ethyl-N-isopropyl-4-(4,4,5,5-tetramethyl-1,3,2-dioxaborolan-2-yl)octanamide (2l)**

63.3 mg, 49% yield (calculated by ^1^H NMR), white solid. R_f_ = 0.4 (PE/ EA = 1:1), using PE/ EA = 3:1 – 1:1 as eluent (1% Et_3_N).

**^1^H NMR** (400 MHz, CDCl_3_) (mixture) δ 5.48 – 5.13 (m, 1H), 4.09 – 3.90 (m, 1H), , 2.03 – 1.94 (m, 2H), 1.63 – 1.53 (m, 2H), 1.26 – 1.18 (m, 6H), 1.16 (s, 12H), 1.07 (d, J = 6.6 Hz, 8H), 0.88 – 0.76 (m, 6H). The spectra showed a mixture of the product and its hydrogenation side product, the yield of product was calculated according to the ratio of the area of the signals δ 2.08 – 2.03 and δ 2.03 – 1.94 (CH_2_ connected to carbonyl group), side product and product mole ratio = 0.84 / 2.02 = 0.42:1, product yield = 63.3 / (0.42*213.4+1*339.3) / 0.3 = 49%.

**^13^C NMR** (101 MHz, CDCl_3_) (mixture) δ 173.2, 172.6, 132.5, 128.7, 127.2, 83.0, 41.1, 38.6, 34.4, 33.8, 32.7, 32.5, 29.6, 29.1, 28.8, 27.0, 26.7, 25.6, 24.9, 23.1, 22.8, 22.8, 14.1, 10.8, 9.1. The signal of the α-B-carbon was not observed.

**HRMS** (ESI) (m/z): Calcd. C_19_H_39_BNO_3_^+^ [M+H]^+^: 340.3018. Found: 340.3026.

**IR** (ν/ cm^−1^, neat): 3258, 2968, 2926, 1635, 1553, 1457, 1377, 1308, 1254, 1212, 1190, 1165, 1142, 981, 966, 856, 789, 714, 685, 602.

**N-isopropyl-3-(1-(4,4,5,5-tetramethyl-1,3,2-dioxaborolan-2-yl)cyclopentyl)propenamide (2m)**

72.4 mg, 53% yield (calculated by ^1^H NMR), white solid. R_f_ = 0.3 (PE/ EA = 3:1), using PE/ EA = 3:1 as eluent (1% Et_3_N).

**^1^H NMR** (400 MHz, CDCl_3_) (mixture) δ 5.38 (d, J = 37.9 Hz, 1H), 4.07 (ddt, J = 10.0, 7.8, 6.5 Hz, 1H), 2.20 – 2.07 (m, 2H), 1.92 (s, 0H), 1.76 (td, J = 9.7, 8.4, 5.2 Hz, 2H), 1.70 – 1.47 (m, 6H), 1.26 (s, 2H), 1.23 (s, 6H), 1.14 (dd, J = 6.6, 1.1 Hz, 6H). The spectra showed a mixture of the product and its hydrogenation side product, the yield of hydrogenation product was calculated according to the ratio of the area of the signals δ1.92 (s, CH on the ring), side product and product mole ratio = 0.44 / 1-0.44 = 0.78:1, product yield = 72.4 / (0.78*183.3+1*309.3) / 0.3 = 53%.

**^13^C NMR** (101 MHz, CDCl_3_) (mixture) δ 172.8, 172.4, 83.1, 41.2, 41.1, 39.8, 36.3, 35.6, 35.1, 34.3, 32.5, 32.0, 25.2, 25.1, 25.0, 24.7, 22.8. The signal of the α-B-carbon was not observed.

**HRMS** (ESI) (m/z): Calcd. C_17_H_33_BNO_3_^+^ [M+H]^+^: 310.2548. Found: 310.2551.

**IR** (ν/ cm^−1^, neat):.3240, 3068, 2941, 2865, 1630, 1547, 1452, 1382, 1304, 1277, 1170, 1141, 1043, 969, 928, 855, 694, 629, 580.

**N-isopropyl-3-(1-(4,4,5,5-tetramethyl-1,3,2-dioxaborolan-2-yl)cyclohexyl)propenamide (2n)**

63.3 mg, 55% yield (calculated by ^1^H NMR), white solid. R_f_ = 0.4 (PE/ EA = 1:1), using PE/ EA = 3:1 – 1:1 as eluent (1% Et_3_N).

**^1^H NMR** (400 MHz, CDCl_3_) (mixture) δ 5.43 – 5.15 (m, 1H), 4.08 – 3.87 (m, 1H), 2.11 – 2.03 (m, 2H), 2.03 – 1.97 (m, 1H), 1.83 – 1.71 (m, 1H), 1.68 – 1.44 (m, 8H), 1.31 – 1.00 (m, 22H), 0.92 (s, 4H). The spectra showed a mixture of the product and its hydrogenation side product, the yield of product was calculated according to the ratio of the area of the signals δ 2.11 – 2.03 and δ 2.03 – 1.97 (CH_2_ connected to carbonyl group), side product and product mole ratio = 0.65 / 2.07 = 0.31:1, product yield = 63.3 / (0.31*197.3+1*323.3) / 0.3 = 55%.

**^13^C NMR** (101 MHz, CDCl_3_) (mixture) δ 172.7, 172.5, 83.1, 41.1, 37.4, 36.2, 34.9, 34.5, 33.4, 33.2, 33.1, 26.5, 26.2, 25.0, 24.9, 22.8, 22.8. The signal of the α-B-carbon was not observed.

**HRMS** (ESI) (m/z): Calcd. C_18_H_35_BNO_3_^+^ [M+H]^+^: 324.2705. Found: 324.2697.

**IR** (ν/ cm^−1^, neat): 3298, 2974, 2922, 2850, 1632, 1543, 1454, 1372, 1364, 1337, 1305, 1275, 1243, 1233, 1173, 1135, 1108, 970, 858, 686, 633, 608.

**N-cyclohexyl-4-methyl-4-(4,4,5,5-tetramethyl-1,3,2-dioxaborolan-2-yl)pentanamide (2o)**

61.1 mg, 41% yield (calculated by ^1^H NMR), white solid. R_f_ = 0.4 (PE/ EA = 1:1), using PE/ EA = 5:1 as eluent (1% Et_3_N).

**^1^H NMR** (400 MHz, DMSO-*d6*) (mixture) δ 7.61 (d, J = 8.3 Hz, 1H), 3.55 – 3.48 (m, 1H), 2.05 – 2.00 (m, 2H), 2.00 – 1.92 (m, 2H), 1.74 – 1.68 (m, 4H), 1.58 – 1.43 (m, 4H), 1.17 (s, 12H), 1.12 – 1.03 (m, 4H), 0.85 (s, 6H) .The spectra showed a mixture of the product and its hydrogenation side product, the yield of product was calculated according to the ratio of the area of the signals δ 2.05 – 2.00 and δ 2.00 – 1.92 (CH_2_ connected to carbonyl group), side product and product mole ratio = 1.87 / 2.08 = 0.90:1, product yield = 61.1 / (0.90*197.3+1*323.3) / 0.3 = 41%.

**^13^C NMR** (101 MHz, DMSO-*d6*) (mixture) δ 171.8, 171.6, 83.2, 47.7, 47.7, 36.6, 34.9, 34.0, 33.0, 32.7, 27.7, 25.7, 25.1, 25.1, 25.0, 22.8. The signal of the α-B-carbon was not observed.

**HRMS** (ESI) (m/z): Calcd. C_18_H_35_BNO_3_^+^ [M+H]^+^: 324.2705. Found: 324.2697.

**IR** (ν/ cm^−1^, neat): 3284, 3073, 2931, 2854, 1630, 1547, 1448, 1367, 1345, 1309, 1276, 1247, 1201, 1133, 965, 892, 854, 715,641, 560.

**N-(tert-butyl)-2-(hydroxymethyl)benzamide (2p)**

57.4 mg, 92% isolated yield, yellow solid. R_f_ = 0.3 (PE/ EA = 3:1), using PE/ EA = 10:1 – 3:1 as eluent.

**^1^H NMR** (400 MHz, CDCl_3_) δ 7.47 (dd, J = 7.6, 1.4 Hz, 1H), 7.41 (td, J = 7.4, 1.4 Hz, 1H), 7.38 – 7.29 (m, 2H), 6.19 (s, 1H), 4.57 (s, 2H), 4.38 (s, 1H), 1.47 (s, 9H).

**^13^C NMR** (101 MHz, CDCl_3_) δ 169.8, 139.7, 137.2, 130.8, 130.6, 128.0, 127.4, 64.6, 52.1, 28.7.

All data matched that reported in the literature^5^.

**N-(tert-butyl)-2-(hydroxymethyl)-4-methylbenzamide (2q)**

58.4 mg, 88% isolated yield, yellow solid. R_f_ = 0.3 (PE/ EA = 3:1), using PE/ EA = 10:1 – 3:1 as eluent.

**^1^H NMR** (400 MHz, CDCl_3_) δ 7.37 (d, J = 7.7 Hz, 1H), 7.15 – 7.07 (m, 2H), 6.30 (s, 1H), 4.73 (s, 1H), 4.56 – 4.45 (m, 2H), 2.35 (s, 3H), 1.46 (s, 9H).

**^13^C NMR** (101 MHz, CDCl_3_) δ 169.9, 141.0, 139.7, 134.3, 131.4, 128.5, 127.6, 127.5, 64.7, 52.0, 28.8, 21.2.

**HRMS** (ESI) (m/z): Calcd. C_13_H_20_NO_2_^+^ [M+H]^+^: 222.1489. Found: 222.1486.

**IR** (ν/ cm^−1^, neat): 3225, 2967, 2923, 1628, 1611, 1550, 1489, 1449, 1390, 1361, 1328, 1278, 1223, 1169, 1147, 1102, 1047, 990, 871, 823, 768, 729, 699, 624.

**N-(tert-butyl)-2-(hydroxymethyl)-4-methoxybenzamide (2r)**

55.4 mg, 78% isolated yield, white solid. R_f_ = 0.4 (PE/ EA = 3:1), using PE/ EA = 5:1 as eluent.

**^1^H NMR** (400 MHz, CDCl_3_) δ 7.42 (d, J = 8.5 Hz, 1H), 6.90 (d, J = 2.6 Hz, 1H), 6.81 (dd, J = 8.5, 2.6 Hz, 1H), 5.99 (s, 1H), 4.71 (t, J = 6.8 Hz, 1H), 4.55 (d, J = 5.7 Hz, 2H), 3.84 (s, 3H), 1.47 (s, 9H).

**^13^C NMR** (101 MHz, CDCl_3_) δ 169.6, 161.3, 142.5, 129.3, 129.0, 116.0, 112.9, 65.1, 55.4, 52.0, 28.8.

**HRMS** (ESI) (m/z): Calcd. C_13_H_20_NO_3_^+^ [M+H]^+^: 238.1438. Found: 238.1438.

**IR** (ν/ cm^−1^, neat): 3225, 2962, 1629, 1610, 1575, 1544, 1507, 1490, 1446, 1423, 1392, 1363, 1328, 1301, 1278, 1220, 1161, 1107, 1056, 1035, 988, 942, 869, 813, 771, 734, 717, 679, 623, 600.

**N-(tert-butyl)-4-chloro-2-(hydroxymethyl)benzamide (2s)**

35.4 mg, 49% isolated yield, white solid. R_f_ = 0.4 (PE/ EA = 3:1), using PE/ EA = 5:1 as eluent.

**^1^H NMR** (400 MHz, CDCl_3_) δ 7.41 (d, J = 8.2 Hz, 1H), 7.36 (d, J = 2.1 Hz, 1H), 7.30 (dd, J = 8.1, 2.2 Hz, 1H), 6.15 (s, 1H), 4.54 (s, 2H), 4.37 (s, 1H), 1.50 (s, 9H).

**^13^C NMR** (101 MHz, CDCl_3_) δ 168.8, 141.7, 136.5, 135.4, 130.6, 128.7, 127.9, 64.1, 52.3, 28.7.

**HRMS** (ESI) (m/z): Calcd. C_12_H_17_ClNO_2_^+^ [M+H]^+^: 242.0942. Found: 242.0940.

**IR** (ν/ cm^−1^, neat): 3217, 3077, 2965, 2925, 1634, 1593, 1563, 1548, 1475, 1453, 1433, 1391, 1364, 1324, 1255, 1222, 1192, 1152, 1112, 1091, 1042, 989, 929, 892, 874, 829, 770, 739, 722, 710, 688, 614.

**4-bromo-N-(tert-butyl)-2-(hydroxymethyl)benzamide (2t)**

47% yield (NMR yield, with catechol impurity), white solid. R_f_ = 0.3 (PE/ EA = 3:1), using PE/ EA = 3:1 as eluent.

**^1^H NMR** (400 MHz, CDCl_3_) δ 7.52 (d, J = 2.0 Hz, 1H), 7.46 (dd, J = 8.1, 2.0 Hz, 1H), 7.34 (d, J = 8.2 Hz, 1H), 6.18 (s, 1H), 4.54 (d, J = 5.9 Hz, 2H), 4.38 (t, J = 6.7 Hz, 1H), 1.47 (s, 9H).

**^13^C NMR** (101 MHz, CDCl_3_) δ 168.9, 141.7, 135.9, 133.6, 131.0, 128.9, 124.9, 64.1, 52.3, 28.7.

**HRMS** (ESI) (m/z): Calcd. C_12_H_17_BrNO_2_^+^ [M+H]^+^: 286.0437. Found: 286.0451.

**IR** (ν/ cm^−1^, neat): 3211, 2965, 1633, 1589, 1554, 1474, 1452, 1391, 1364, 1326, 1219, 1190, 1083, 1043, 990, 883, 827, 770, 827, 770, 740, 709, 680.

**N-(tert-butyl)-2-(1-hydroxyethyl)benzamide (2u)**

41.6 mg, 63% isolated yield, white solid. R_f_ = 0.25 (PE/ EA = 3:1), using PE/ EA = 5:1 as eluent.

**^1^H NMR** (400 MHz, CDCl_3_) δ 7.47 – 7.34 (m, 3H), 7.31 – 7.22 (m, 1H), 6.14 (s, 1H), 5.03 – 4.87 (m, 1H), 4.80 (d, J = 2.2 Hz, 1H), 1.56 – 1.49 (m, 3H), 1.46 (d, J = 2.3 Hz, 9H).

**^13^C NMR** (101 MHz, CDCl_3_) δ 170.8, 143.5, 136.6, 130.5, 127.4, 127.3, 126.9, 68.1, 52.1, 28.7, 21.9.

**HRMS** (ESI) (m/z): Calcd. C_13_H_20_NO_2_^+^ [M+H]^+^: 222.1489. Found: 222.1488.

**IR** (ν/ cm^−1^, neat): 3461, 3297, 2973, 2929, 1643, 1630, 1598, 1576, 1532, 1455, 1392, 1363, 1317, 1270, 1221, 1200, 1118, 1070, 1048, 1012, 898, 876, 796, 760, 664, 610.

**N-(tert-butyl)-4-hydroxy-4-phenylbutanamide (2v)**

46% yield (NMR yield, with EtOAc), white solid. R_f_ = 0.3 (PE/ EA = 1:1), using PE/ EA = 3:1 as eluent.

**^1^H NMR** (400 MHz, CDCl_3_) δ 7.39 – 7.21 (m, 5H), 5.45 (s, 1H), 4.77 (dd, J = 7.8, 4.3 Hz, 1H), 4.02 (s, 1H), 2.25 (t, J = 6.6 Hz, 2H), 2.00 (dd, J = 14.3, 7.3 Hz, 2H), 1.34 (s, 9H).

**^13^C NMR** (101 MHz, CDCl_3_) δ173.0, 144.7, 128.4, 127.3, 125.8, 73.7, 51.4, 34.4, 33.9, 28.8.

**HRMS** (ESI) (m/z): Calcd. C_14_H_22_NO_2_^+^ [M+H]^+^: 236.1645. Found: 236.1640.

**IR** (ν/ cm^−1^, neat): 3295, 3083, 2967, 2924, 2361, 1642, 1547, 1452, 1392, 1363, 1272, 1223, 1059, 913, 748, 699.

**N-(tert-butyl)-4-hydroxy-4-(p-tolyl)butanamide (2w)**

32.3 mg, 43% isolated yield, white solid. R_f_ = 0.35 (PE/ EA = 1:1), using PE/ EA = 3:1 as eluent.

**^1^H NMR** (400 MHz, CDCl_3_) δ 7.25 (d, J = 7.7 Hz, 2H), 7.15 (d, J = 7.8 Hz, 2H), 5.40 (s, 1H), 4.73 (dd, J = 7.5, 4.7 Hz, 1H), 3.74 (s, 1H), 2.34 (s, 3H), 2.24 (t, J = 6.7 Hz, 2H), 2.09 – 1.96 (m, 2H), 1.34 (s, 9H).

**^13^C NMR** (101 MHz, CDCl_3_) δ 172.9, 141.6, 136.9, 129.1, 125.7, 73.6, 51.4, 34.3, 33.9, 28.8, 21.1.

**HRMS** (ESI) (m/z): Calcd. C_15_H_24_NO_2_^+^ [M+H]+: 250.1802. Found: 250.1798.

**IR** (ν/ cm^−1^, neat): 3290, 3082, 2964, 2919, 1634, 1564, 1439, 1409, 1388, 1328, 1278, 1222, 1071, 1026, 934, 894, 856, 825, 774, 706, 675, 591.

**(S)-N-(tert-butyl)-2-methoxy-4-methyl-4-(4,4,5,5-tetramethyl-1,3,2-dioxaborolan-2-yl)pentanamide (2x)**

42.2 mg, 43% isolated yield, white solid. R_f_ = 0.30 (PE/ EA = 3:1), using PE/ EA = 10:1 as eluent.

**^1^H NMR** (400 MHz, CDCl_3_) δ 6.21 (s, 1H), 3.48 (dd, J = 11.1, 3.2 Hz, 1H), 3.31 (s, 3H), 1.75 (dd, J = 14.0, 11.1 Hz, 1H), 1.53 (dd, J = 14.0, 3.2 Hz, 1H), 1.36 (s, 9H), 1.24 (d, J = 5.8 Hz, 12H), 0.94 (s, 6H).

**^13^C NMR** (126 MHz, CDCl_3_) δ 172.8, 82.8, 81.7, 58.2, 50.5, 44.7, 28.8, 26.5, 24.9, 24.6, 23.0. The signal of the α-B-carbon was not observed.

**HRMS** (ESI) (m/z): Calcd. C_17_H_35_BNO_4_^+^ [M+H]+: 328.2654. Found: 328.2661.

**IR** (ν/ cm^−1^, neat): 3270, 2971, 1644, 1556, 1473, 1457, 1385, 1364, 1340, 1326, 1303, 1260, 1228, 1195, 1136, 1114, 1067, 1049, 1021, 968, 857, 809, 747, 694, 672.

**(S)-1-(tert-butylamino)-4-methyl-1-oxo-4-(4,4,5,5-tetramethyl-1,3,2-dioxaborolan-2-yl)pentan-2-yl acetate (2y)**

83.7 mg, 79% isolated yield, colorless oil. R_f_ = 0.5 (PE/ EA = 1:1), using PE/ EA = 10:1 - 3:1 as eluent (1% Et_3_N).

**^1^H NMR** (400 MHz, CDCl_3_) δ 5.85 (s, 1H), 4.90 (dd, J = 8.9, 2.8 Hz, 1H), 2.12 (s, 3H), 1.90 – 1.81 (m, 1H), 1.76 – 1.70 (m, 1H), 1.35 (s, 9H), 1.25 (d, J = 3.2 Hz, 12H), 0.96 (s, 6H).

**^13^C NMR** (101 MHz, CDCl_3_) δ 170.3, 170.0, 83.4, 73.7, 51.1, 42.2, 28.8, 25.3, 24.8, 24.7, 24.3, 21.1. The signal of the α-B-carbon was not observed.

**HRMS** (ESI) (m/z): Calcd. C_18_H_35_BNO_5_^+^ [M+H]^+^: 356.2603. Found: 356.2613.

**IR** (ν/ cm^−1^, neat): 3327, 2973, 2934, 1743, 1672, 1523, 1475, 1455, 1391, 1367, 1309, 1223, 1136, 1077, 967, 853. 690.

**(S)-N-(tert-butyl)-2-(1,3-dioxoisoindolin-2-yl)-4-methyl-4-(4,4,5,5-tetramethyl-1,3,2-dioxaborolan-2-yl)pentanamide (2z)**

59.1 mg, 45% isolated yield, white solid. R_f_ = 0.3 (PE/ EA = 5:1), using PE/ EA = 10:1 as eluent (1% Et_3_N).

**^1^H NMR** (400 MHz, CDCl_3_) δ 7.76 (dd, J = 5.4, 3.1 Hz, 2H), 7.63 (dd, J = 5.5, 3.1 Hz, 2H), 6.36 (s, 1H), 4.63 (dd, J = 9.2, 2.1 Hz, 1H), 2.41 (dd, J = 15.2, 9.2 Hz, 1H), 1.95 (dd, J = 15.2, 2.1 Hz, 1H), 1.29 (s, 9H), 1.15 (d, J = 9.2 Hz, 12H), 0.92 (s, 3H), 0.81 (s, 3H).

**^13^C NMR** (101 MHz, CDCl_3_) δ 168.0, 167.4, 132.9, 131.1, 122.3, 82.6, 53.6, 50.4, 38.7, 27.8, 24.6, 23.8, 23.3, 22.7. The signal of the α-B-carbon was not observed.

**HRMS** (ESI) (m/z): Calcd. C_24_H_36_BN_2_O_5_^+^ [M+H]^+^: 443.2712. Found: 443.2714.

**IR** (ν/ cm^−1^, neat): 3379, 2965, 2921, 1774, 1713, 1691, 1526, 1476, 1454, 1386, 1313, 1260, 1222, 1137, 1083, 1016, 848, 797, 719, 690.

**(3R,5R,8R,9S,10S,13S,14S,17R)-17-((S)-5-(tert-butylamino)-5-oxo-2-(4,4,5,5-tetramethyl-1,3,2-dioxaborolan-2-yl)pentan-2-yl)-10,13-dimethylhexadecahydro-1H-cyclopenta[a]phenanthren-3-yl acetate (2aa)**

130.4 mg, 72% isolated yield, white solid. R_f_ = 0.5 (PE/ EA = 3:1), using PE/ EA = 10:1 - 5:1 as eluent (1% Et_3_N).

**^1^H NMR** (400 MHz, DMSO-*d6*) δ 7.34 (s, 1H), 4.59 (ddd, J = 16.1, 11.0, 4.3 Hz, 1H), 2.18 – 1.89 (m, 7H), 1.89 – 1.72 (m, 5H), 1.72 – 1.56 (m, 3H), 1.56 – 1.44 (m, 3H), 1.44 – 1.26 (m, 9H), 1.22 (s, 12H), 1.19 – 1.12 (m, 4H), 1.09 (d, J = 9.4 Hz, 4H), 1.00 (dd, J = 14.0, 10.6 Hz, 4H), 0.90 (s, 3H), 0.87 (d, J = 6.5 Hz, 3H), 0.61 (s, 3H).

**^13^C NMR** (101 MHz, DMSO-*d6*) δ 172.6, 170.3, 74.0, 56.4, 56.1, 50.2, 42.7, 41.6, 35.8, 35.4, 35.0, 34.6, 33.6, 32.4, 32.1, 29.0, 28.2, 27.1, 26.7, 26.4, 24.3, 23.5, 21.6, 20.9, 18.9, 12.3. The signal of the α-B-carbon was not observed.

**HRMS** (ESI) (m/z): Calcd. C_36_H_63_BNO_5_^+^ [M+H]^+^: 600.4794. Found: 600.4796.

**IR** (ν/ cm^−1^, neat): 3287, 3077, 2928, 2866, 1733, 1640, 1596, 1550, 1450, 1362, 1324,1242, 1156, 1091, 1024, 969, 885, 844, 708, 606.

**N-(((1R,4aS,10S,10aR)-7-isopropyl-1,4a-dimethyl-10-(4,4,5,5-tetramethyl-1,3,2-dioxaborolan-2-yl)-1,2,3,4,4a,9,10,10a-octahydrophenanthren-1-yl)methyl)benzamide (2ab)**

132.4 mg**,** 46% yield (calculated by ^1^H NMR), white solid. R_f_ = 0.3 (PE/ EA = 3:1), using PE/ EA = 10:1 - 3:1 as eluent (1% Et_3_N).

**^1^H NMR** (400 MHz, CDCl_3_) (mixture) δ 7.72 (ddt, J = 8.6, 3.2, 2.0 Hz, 4H), 7.52 – 7.31 (m, 6H), 7.20 – 6.83 (m, 6H), 6.59 (dd, J = 9.6, 3.1 Hz, 1H), 6.24 – 6.10 (m, 2H), 6.05 (dd, J = 9.7, 2.7 Hz, 1H), 3.67 (dd, J = 13.8, 7.9 Hz, 1H), 3.49 – 3.26 (m, 2H), 3.15 (dd, J = 13.8, 5.1 Hz, 1H), 2.99 – 2.73 (m, 4H), 2.29 (dt, J = 16.2, 3.4 Hz, 2H), 2.21 – 2.12 (m, 1H), 2.00 – 1.93 (m, 1H), 1.92 – 1.45 (m, 11H), 1.44 – 1.29 (m, 5H), 1.28 – 1.16 (m, 18H), 1.10 (d, J = 14.9 Hz, 6H), 1.00 (s, 5H).The spectra showed a mixture of the product and its hydrogenation side product, the yield of product was calculated according to the ratio of the area of the signals δ 3.67 (dd, J = 13.8, 7.9 Hz, 1H), 3.49 – 3.26 (m, 2H), side product and product mole ratio = 2.28/2: 1 = 1.14:1, product yield = 132.4/ (1.14*389.6+1*515.5) / 0.3 = 46%.

**^13^C NMR** (101 MHz, CDCl_3_) (mixture) δ 167.9, 167.7, 147.1, 146.3, 145.6, 145.5, 134.9, 134.8, 132.5, 131.4, 128.9, 128.6, 128.6, 128.4, 128.2, 127.2, 127.0, 126.9, 126.9, 125.9, 124.8, 124.3, 123.9, 121.8, 83.7, 50.4, 49.8, 46.9, 45.9, 38.4, 37.7, 37.6, 37.2, 36.4, 35.7, 35.7, 33.6, 33.4, 30.5, 25.5, 24.9, 24.0, 24.0, 20.8, 19.3, 19.1, 18.8, 18.7, 18.4. The signal of the α-B-carbon was not observed.

**HRMS** (ESI) (m/z): Calcd. C_33_H_47_BNO_5_^+^ [M+H]^+^: 516.3643. Found: 516.3652.

**IR** (ν/ cm^−1^, neat): 3350, 2951, 2926, 2851, 1637, 1578, 1535, 1491, 1444, 1383, , 1319, 1293, 1231, 1077, 1026, 959, 904, 882, 823, 798, 721, 698, 674, 635, 607.

### 4.3 Failed examples

Here are some failed examples that we attempted previously. Specifically, when we introduced a double bond or a six-membered ring into the molecule, we were unable to obtain the corresponding borylation product. This may be due to the inability to form a six-membered ring transition state. Additionally, primary alkyl C-H bond and aryl C-H bond substrates with higher bond energy were found to be unsuitable for this reaction, indicating that the remote C-H bond energy may influence the 1,5-HAT process of this reaction. Furthermore, when we replaced the tert-butyl group with methyl or benzyl, we were unable to obtain the corresponding tertiary C-H bond borylation product. This may be attributed to the fact that the nitrogen-linked group has a greater impact on the N-H bond energy.

## Mechanism studies

### 5.1 Reaction with TEMPO

In a glovebox under a nitrogen atmosphere, a 10mL Schlenk tube with a stir bar was sequentially added B_2_cat_2_ (0.75 mmol, 1.5 equiv., 178 mg), **1a** (0.3 mmol, 1.0 equiv., 108 mg), TEMPO (0.9 mmol, 3.0 equiv., 141 mg), Eosin Y (5%, 0.015 mmol, 10.5 mg) and 3 mL DMA was added, followed by DIPEA (0.6 mmol, 2.0 equiv., 105 μL). The capped Schlenk tube was removed from the glovebox, and the reaction mixture was irradiated by 467nm Kessil 40W blue LED for 12 hours, with a fan for cooling. After cooling to room temperature, pinacol (142 mg, 4.0 equiv.) and 1.0 mL triethylamine were added, and the mixture was stirred at room temperature for 1 hour.

Water was added to the reaction mixture, and the aqueous layer was extracted with EtOAc (20ml x 2). If phase separation was slow, brine was added. The organic phases were combined and washed with 50 mL of saturated brine, the organic phase was dried over anhydrous sodium sulfate and filtered, then concentrated under reduced pressure, purified by column chromatography to give the TEMPO-trapped product **2ad** in 16% yield as a white solid, the borylation product was not detected. The product was monitored by thin-layer chromatography with phosphomolybdic acid (PMA) stain.

**N-(tert-butyl)-4-methyl-4-((2,2,6,6-tetramethylpiperidin-1-yl)oxy)pentanamide (3)**

15.7 mg, 16% isolated yield, white solid. R_f_ = 0.3 (PE/ EA = 10:1), using PE/ EA = 20:1 as eluent.

**^1^H NMR** (400 MHz, CDCl_3_) δ 5.26 (s, 1H), 2.32 – 2.24 (m, 2H), 1.94 – 1.85 (m, 2H), 1.73 (s, 2H), 1.46 (d, J = 2.9 Hz, 2H), 1.35 (s, 9H), 1.26 (s, 8H), 1.10 (d, J = 17.5 Hz, 12H).

**^13^C NMR** (101 MHz, CDCl_3_) δ 173.1, 78.0, 59.2, 51.0, 40.8, 39.2, 34.8, 32.5, 28.9, 26.9, 20.8, 17.1.

**HRMS** (ESI) (m/z): Calcd. C_19_H_39_N_2_O_2_^+^ [M+H]^+^: 327.3006. Found: 327.3004.

**IR** (ν/ cm^−1^, neat): 3277, 3081, 2972, 2931, 1642, 1556, 1454, 1373, 1360, 1336, 1294, 1258, 1229, 1173, 1132, 1118, 956, 929, 918, 879, 712, 636.

### 5.2 Radical clock reaction

4-cyclopropylbutanoic acid was prepared according to literature^6^. **1ac** was prepared by **General procedure** to afford a colorless oil in 39% yield.

In a glovebox under a nitrogen atmosphere, a 10mL Schlenk tube with a stir bar was sequentially added B_2_cat_2_ (0.75 mmol, 1.5 equiv., 60 mg), **1ac** (0.1 mmol, 1.0 equiv., 37 mg), Eosin Y (5%, 0.005 mmol, 3.5 mg) and 2 mL DMA was added, followed by DIPEA (0.2 mmol, 2.0 equiv., 35 μL). The capped Schlenk tube was removed from the glovebox, and the reaction mixture was irradiated by 467nm Kessil 40W blue LED for 12 hours, with a fan for cooling. After cooling to room temperature, pinacol (47 mg, 4.0 equiv.) and 0.5 mL triethylamine were added, and the mixture was stirred at room temperature for 1 hour.

Water was added to the reaction mixture, and the aqueous layer was extracted with EtOAc (20 mL x 2). If phase separation was slow, brine was added. The organic phases were combined and washed with 30 mL of saturated brine, the organic phase was dried over anhydrous sodium sulfate and filtered, then concentrated under reduced pressure, purified by column chromatography to give the ring-opening borylation product **2ac** as colorless oil, 32% yield. (Using KMnO_4_ stain for detection).

**N-((argiocarbonyl)oxy)-N-(tert-butyl)-4-cyclopropylbutanamide (1ac)**

39% overall yield, colorless oil.

**^1^H NMR** (400 MHz, CDCl_3_) δ 8.23 (d, J = 8.1 Hz, 2H), 7.81 (d, J = 8.2 Hz, 2H), 2.33 (dt, J = 15.4, 7.6 Hz, 1H), 2.14 (dt, J = 16.0, 7.5 Hz, 1H), 1.70 (pd, J = 7.3, 1.2 Hz, 2H), 1.49 (s, 9H), 1.17 (q, J = 7.2 Hz, 2H), 0.65 – 0.51 (m, 1H), 0.40 – 0.27 (m, 2H), -0.03 – -0.09 (m, 2H).

**^13^C NMR** (101 MHz, CDCl_3_) δ 174.8, 164.5, 135.8 (q, J = 33.0 Hz), 130.4, 130.2, 126.0 (q, J = 3.6 Hz), 123.3 (q, J = 273.3 Hz), 62.8, 34.0, 34.0, 27.5, 24.1, 10.5, 4.3.

**HRMS** (ESI) (m/z): Calcd. C_19_H_25_F_3_NO_3_^+^ [M+H]^+^: 372.1781. Found: 372.1775.

**IR** (ν/ cm^−1^, neat): 2987, 2933, 1768, 1678, 1412, 1365, 1323, 1251, 1235, 1204, 1172, 1132, 1065, 1045, 1011, 861, 822, 770, 700.

**N-(tert-butyl)-7-(4,4,5,5-tetramethyl-1,3,2-dioxaborolan-2-yl)hept-4-enamide (2ac)**

32% yield, colorless oil. R_f_ = 0.5 (PE / EA = 3:1), using PE / EA = 10:1 as eluent.

**^1^H NMR** (400 MHz, CDCl_3_) δ 5.53 (dt, J = 13.6, 6.3 Hz, 1H), 5.44 – 5.34 (m, 1H), 5.27 (s, 1H), 2.36 – 2.22 (m, 2H), 2.16 – 2.02 (m, 4H), 1.33 (d, J = 2.6 Hz, 9H), 1.24 (d, J = 1.7 Hz, 12H), 0.84 (t, J = 7.7 Hz, 2H).

**^13^C NMR** (101 MHz, CDCl_3_) δ 171.9, 133.5, 127.5, 83.0, 51.1, 37.6, 28.9, 28.8, 26.8, 24.8, 23.5, 21.7. The signal of the α-B-carbon was not observed.

**HRMS** (ESI) (m/z): Calcd. C_17_H_33_BNO_3_^+^ [M+H]^+^: 310.2548. Found: 310.2555.

**IR** (ν/ cm^−1^, neat): 3308, 2974, 2927, 1646, 1545, 1423, 1368, 1321, 1267, 1225, 1143, 1109, 967, 868, 847, 802, 672.

### 5.3 Ultraviolet–visible absorption spectroscopy

UV-vis absorption spectrum was measured by a UV-visible spectrophotometer (Thermo Evolution^TM^ 220), and the concentration of all compounds was 5 x 10^-4^ M in DMA, and the wavelength range was 300 – 700 nm. The data was analyzed by Origin 2022, and the absorption spectrum was normalized for comparison.


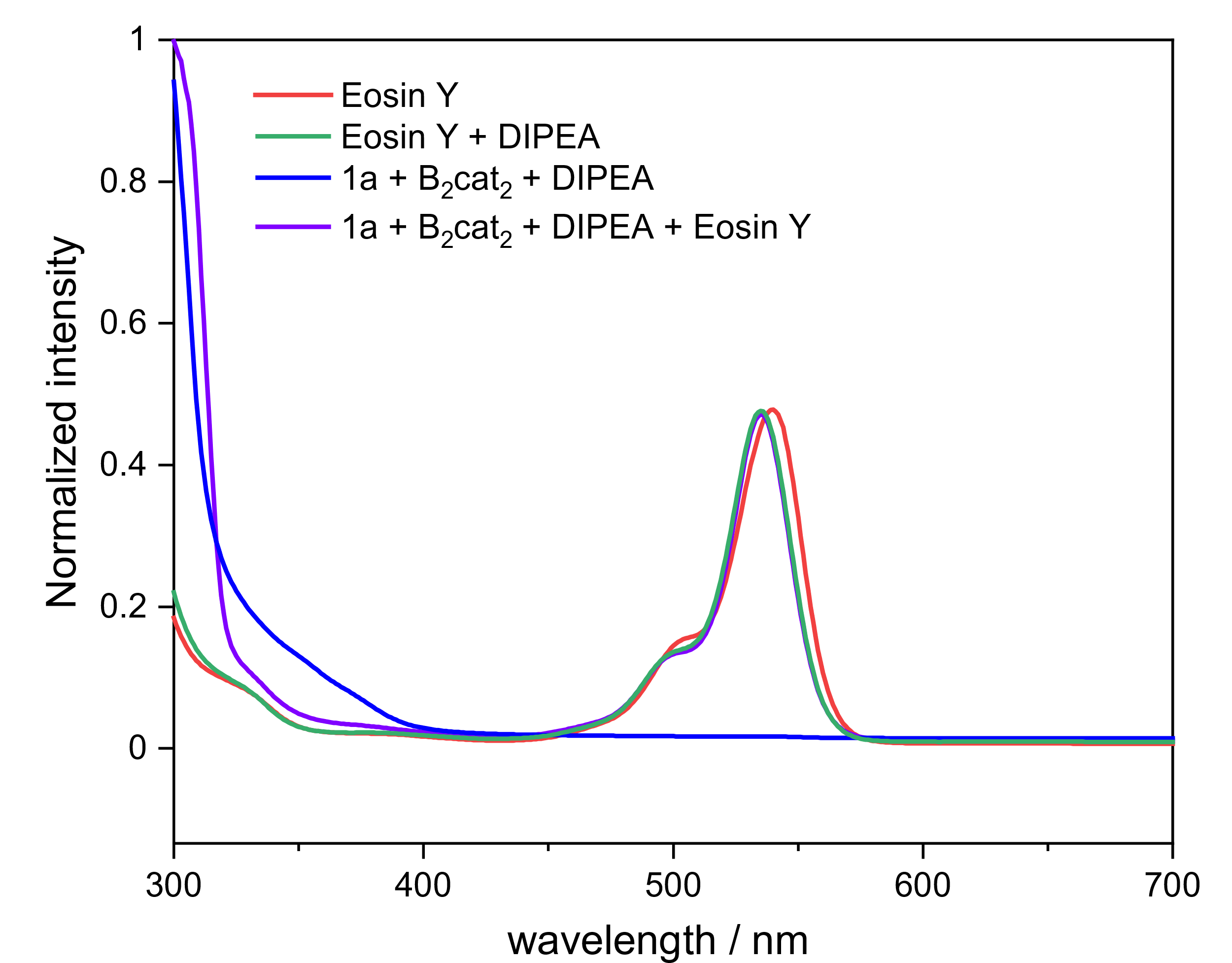


**Supplementary Figure 2** UV-vis absorption spectrum.

It showed that the photocatalyst Eosin Y was the only absorbing species in the reaction near the excitation wavelength (λ_max_ = 467 nm), absorbing peak is at 539nm, when DIPEA was added, the absorbing peak moved to 535 nm.

### 5.4 Light on-off experiment

To study the necessity of continuous irradiation with blue LED for the radical chain process, we conducted a light on-off experiment. The experiment following the standard procedure: In a glovebox under a nitrogen atmosphere, sequentially added B_2_cat_2_ (0.75 mmol, 1.5 equiv., 178 mg), the substrate (0.3 mmol, 1.0 equiv.), Eosin Y (5%, 0.015 mmol, 10.5 mg) and 3 mL DMA to a 10 mL Schlenk tube with a stir bar, followed by DIPEA (0.6 mmol, 2.0 equiv., 105 μL), decane (19.2 mg) was added as internal standard, The capped Schlenk tube was removed from the glovebox, and the reaction mixture was irradiated by 467nm Kessil 40W blue LED, with a fan for cooling. The reaction tube was irradiated discontinuously and after each light-phase (2 h) and each dark phase (2 h) for 14 hours in total. After each phase, 100 μL reaction mixture was taken via syringe in glovebox, 5 mg pinacol and 50 μL triethylamine were added, and the mixture was stirred at room temperature for 1 hour. After that, the reaction mixture was washed with H_2_O (2 mL), extracted using ethyl acetate (2 mL x 2), and the yield was then measured by GC-FID.

The light on-off experiment showing no product formation in the dark phases. This indicates the requirement for continuous irradiation in this reaction. Without light, this radical chain process will not proceed.

| Time / h | Yield of **2a** / % |
| --- | --- |
| 2 | 13 |
| 4 | 13 |
| 6 | 20 |
| 8 | 20 |
| 10 | 37 |
| 12 | 37 |
| 14 | 48 |

**Supplementary Table 2** Results of the light on-off experiment for the formation of product **2a**.


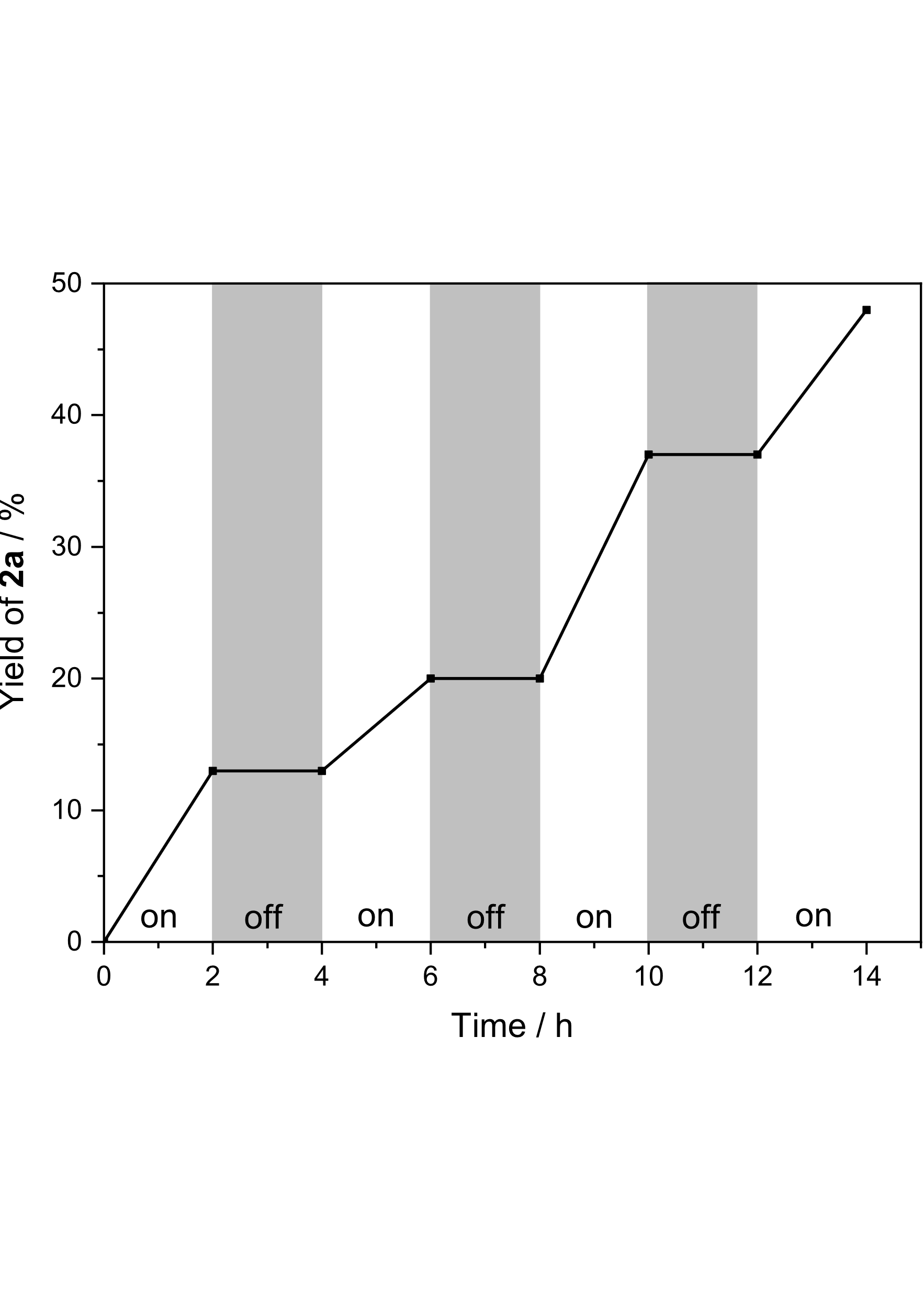


**Supplementary Figure 3** Visible light irradiation on/off experiment.

### 5.5 Reaction with radical trapping reagent CHANT and mass spectrum analysis

**5.5.1. Reaction with radical trapping reagent CHANT**

**N-cyclohexyl-2-(((2,2,6,6-tetramethylpiperidin-1-yl)oxy)methyl)acrylamide (CHANT)**

The trap **CHANT** was synthesized according to literature^7^.

**^1^H NMR** (400 MHz, CDCl_3_) 6.60 (d, J = 8.0 Hz, 1H), 6.08 (d, J = 1.7 Hz, 1H), 5.48 (d, J = 1.6 Hz, 1H), 4.51 (d, J = 1.0 Hz, 2H), 3.84 (tdt, J = 11.5, 7.9, 3.9 Hz, 1H), 2.05 – 1.94 (m, 2H), 1.74 (dp, J = 11.3, 3.8 Hz, 2H), 1.62 (d, J = 3.0 Hz, 1H), 1.52 – 1.44 (m, 4H), 1.47 – 1.30 (m, 4H), 1.26 – 1.13 (m, 9H), 1.11 (s, 6H).

All data matched that reported in the literature^7^.

In a glovebox under a nitrogen atmosphere, a 10mL Schlenk tube with a stir bar was sequentially added B_2_cat_2_ (0.75 mmol, 1.5 equiv., 178 mg), **1a** (0.3 mmol, 1.0 equiv., 108 mg), **CHANT** (0.06 mmol, 0.2 equiv., 19.4 mg), Eosin Y (5%, 0.015 mmol, 10.5 mg) and 3 mL DMA was added, followed by DIPEA (0.6 mmol, 2.0 equiv., 105 μL). The capped Schlenk tube was removed from the glovebox, and the reaction mixture was irradiated by 467nm Kessil 40W blue LED for 12 hours, with a fan for cooling. After cooling to room temperature, pinacol (142 mg, 4.0 equiv.) and 1.0 mL triethylamine were added, and the mixture was stirred at room temperature for 1 hour. Water was added to the reaction mixture, and the aqueous layer was extracted with EtOAc (20ml x 2). If phase separation was slow, brine was added. The organic phases were combined and washed with 30 mL of saturated brine, the organic phase was dried over anhydrous sodium sulfate and filtered, then concentrated under reduced pressure, the sample was sent for mass analysis.

A high resolution solariX XR FTMS (solariX) mass spectrometer (m/z ±0.0001 precision, >10^6^ maximum resolution, mass accuracy less than 1ppm (internal)) using positive ion mode electrospray ionization (Pos ESI-MS) was used for MS characterization of radical trapping samples. Tandem MS was performed similarly to standard MS. The mass result was analyzed by Origin 2022 by OriginLab.

**5.5.2. Radical trapping Mechanism**

As shown in **Supplementary Figure 4**, Radical trapping proceeds via a homolytic substitution reaction S_H_2′. The key design feature is the presence of a good radical leaving group (a nitroxide) at the allylic position of a terminal alkene. Reaction of a short-lived radical with the trap releases the nitroxide radical and yields a stable, non-radical product^7^.

**Supplementary Figure 4**: S_H_2′ Trapping mechanism of CHANT. Cy = cyclohexyl. TEMPO = 2,2,6,6-Tetramethylpiperidine 1-oxyl.

As shown in **Supplementary Figure 5**, the possible trapped radicals in reaction mechanism are nitrogen-centered radical **R1** and carbon-centered radical **R2,** they are isomers (C_20_H_36_N_2_O_2_).

**Supplementary Figure 5**: Possible mechanism of the reaction. Trapped radicals are **R1** (shown in green) and **R2** (shown in blue).

**5.5.3. Mass spectrum analysis**





**Supplementary Figure 6** Mass spectrum from the reaction with 0.2 equiv. CHANT, showing peaks corresponding to unreacted CHANT (m/z 323.2692) and trapped **R1/R2** (C_20_H_37_N_2_O_2_^+^, m/z 337.2849, shown in red).

**Supplementary Table 3** Species identified from radical trapping of the reaction.

| Identified species | Chemical formula | Predicted  *m/z* | Observed  *m/z* | Relative  intensity  / % | Implies  radical |
| --- | --- | --- | --- | --- | --- |
| [CHANT+H]^+^ | C_19_H_35_N_2_O_2_^+^ | 323.2693 | 323.2693 | 100 | / |
|   or   | C_20_H_37_N_2_O_2_^+^ | 337.2850 | 337.2849 | 14.6 | R1/R2 |


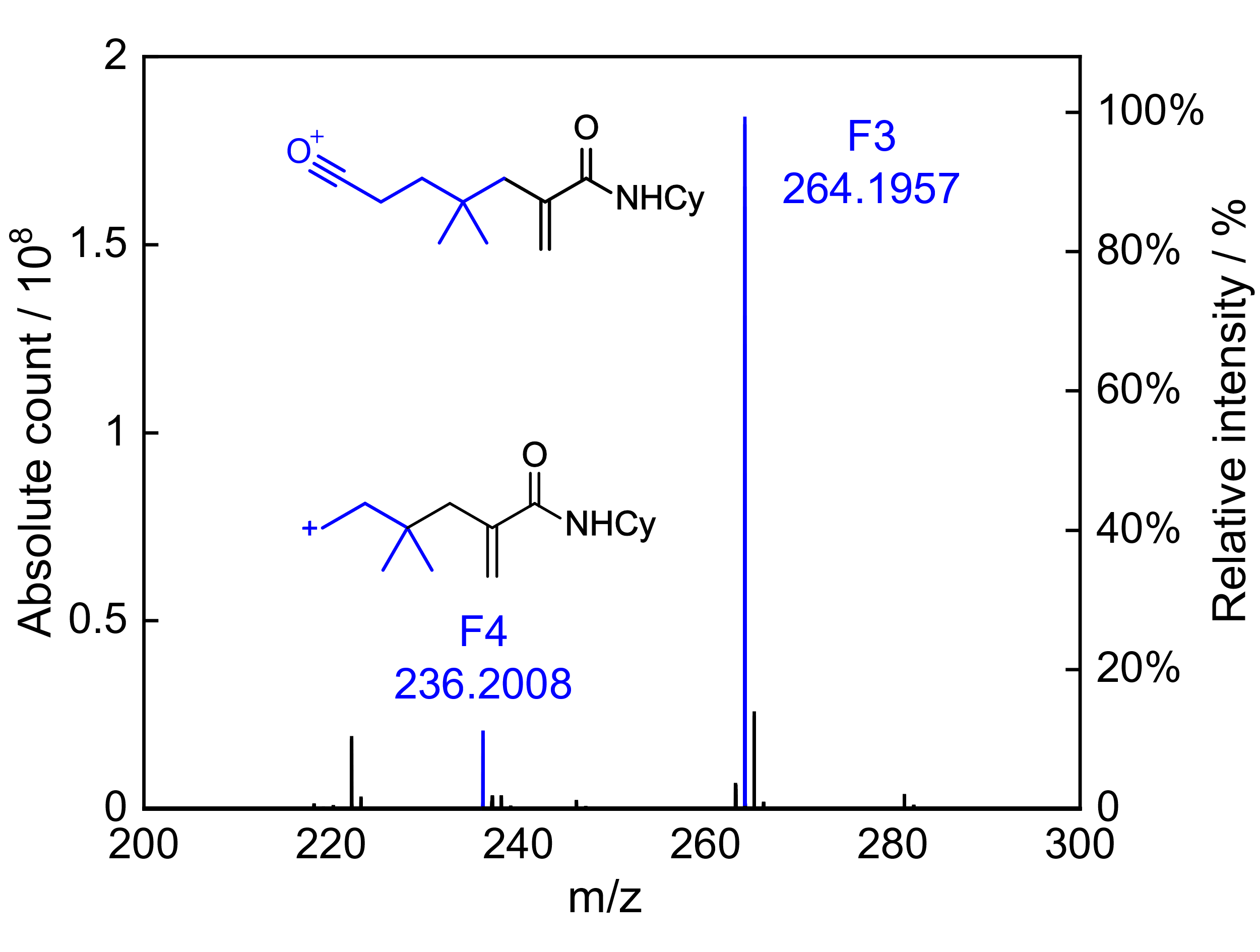

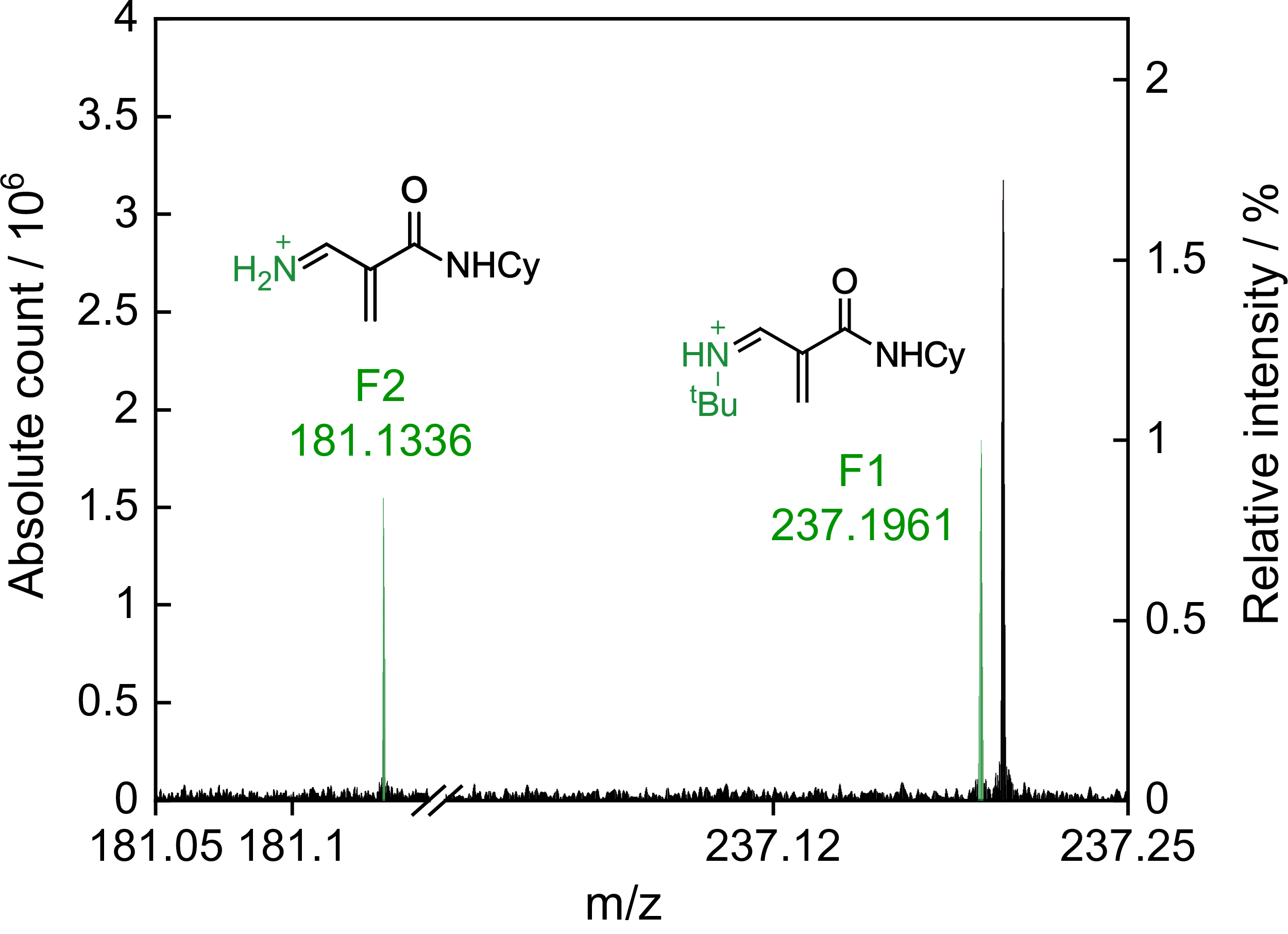


**Supplementary Figure 7.** Tandem mass spectrum performed upon the peak corresponding to trapped **R1**/**R2** (C_20_H_37_N_2_O_2_^+^, m/z 337.2849) from post-trapping of the reaction.

In order to distinguish trapped **R1/R2**, the tandem mass spectrometry was analyzed at m/z 337.2489, the two low-intensity peaks **F1** (m/z 237.1961) and **F2** (m/z 181.1336) which contain two nitrogen atoms, could only be attributed to the trapped **R1**, the fragments showed that the acyl group RCO was dissociated. The most strong-intensity peaks **F3** (m/z 264.1957) is a stable acylium ions, and **F4** (m/z 236.2008) is **F3** losing CO. They could only be attributed to the trapped **R2**, and many other strong peaks could also be attributed to it. This experiment proved that the existence of **R1** and **R2**. The intensity of the trapped **R2** is 100 times greater than the trapped R1, assuming a similar ionization efficiency of the trapped **R1** and **R2** species, it suggests that the 1,5-HAT process is very fast and the carbon-centered radical **R2** is the resting state for this radical chain process.

**Supplementary Table 4** Observed fragment m/z and suggested structures identified from tandem MS upon peak corresponding to **R1 (shown in green)** or **R2 (shown in blue)** from post-trapping of the reaction.

| Identified species | Chemical formula | Predicted  *m/z* | Observed  *m/z* | Relative  intensity  / % | Implies  radical |
| --- | --- | --- | --- | --- | --- |
|  | C_16_H_26_NO_2_^+^ | 264.1958 | 264.1957 | 100 | R2 |
|  | C_10_H_16_NO_2_^+^ | 182.1176 | 182.1176 | 17.6 | R2 |
|  | C_15_H_26_NO^+^ | 236.2009 | 236.2008 | 11.1 | R2 |
|  | C_14_H_24_NO^+^ | 222.1852 | 222.1852 | 10.3 | R2 |
|   or   | C_16_H_29_N_2_O_2_^+^ | 281.2224 | 281.2224 | 1.9 | R1/R2 |
|  | C_14_H_25_N_2_O^+^ | 237.1961 | 237.1961 | 1.0 | R1 |
|  | C_10_H_17_N_2_O^+^ | 181.1335 | 181.1336 | 0.9 | R1 |

## DFT computational data

All density functional theory (DFT) calculations were performed with Gaussian 09w and supported by National Supercomputing Center in Shenzhen. All geometry optimizations and vibrational frequency analysis were computed at the B3LYP (or uB3LYP for open shell systems) level of theory with 6-31+G(d) basis set. Based on the gas phase optimized structures, the single point (SP) energies were calculated with the M062X (or uM062X for open shell systems) functional and a basis set of 6-311+G(d,p), and solvent energy corrections were calculated using the SMD model with DMA as the solvent. The Cartesian coordinates of the structures were shown in Supplementary Data 1.

### 6.1 Table of energies and lowest frequencies


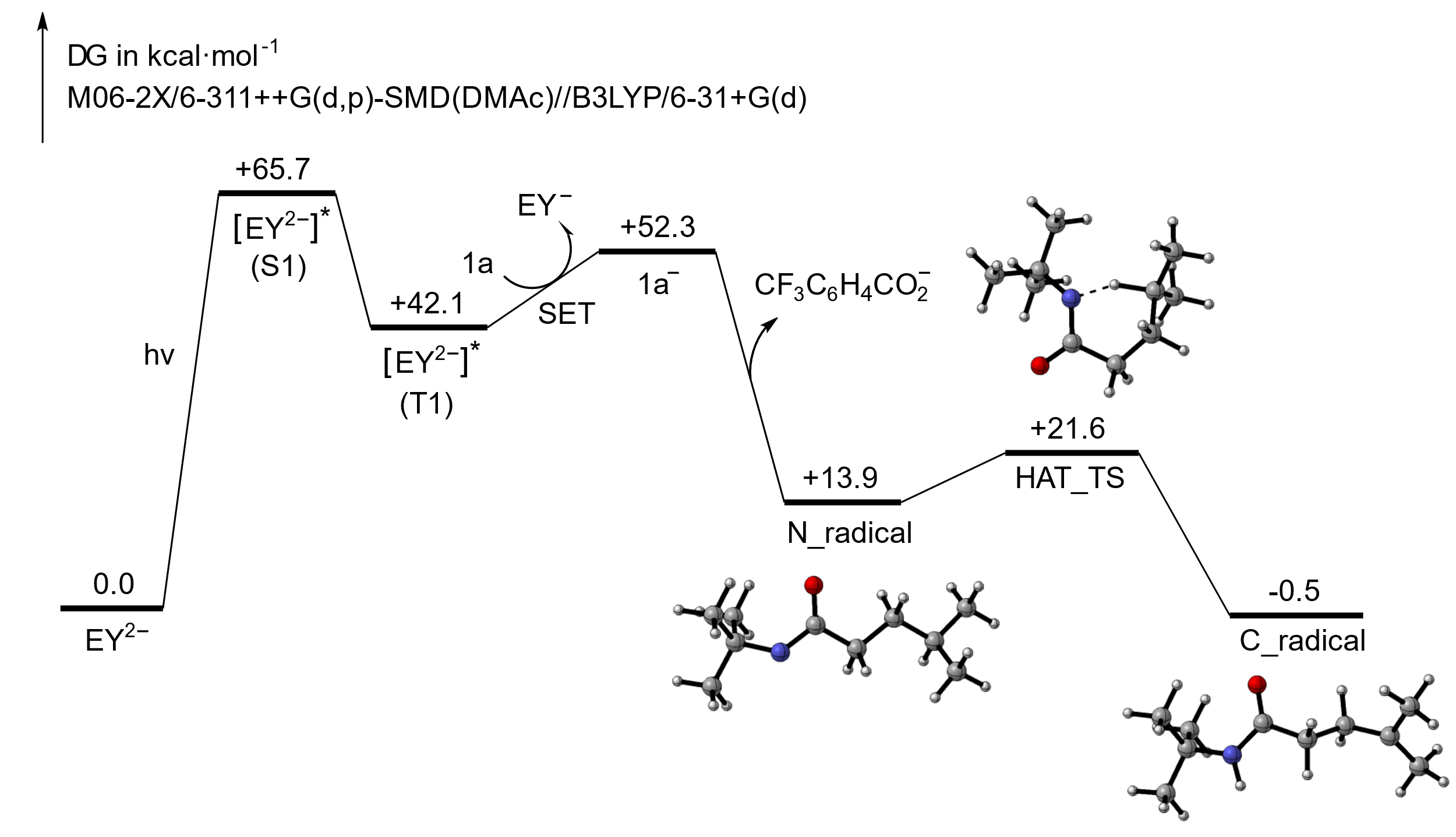


**Supplementary Table 5** The correlation between the type of remote C−H bonds and reaction parameters.

In **Supplementary Table 6**, the energies are in units of Hartree (1 Hartree = 627.5 kCal/mol = 2625 kJ/mol), and the lowest frequencies are in units of cm^-1^. A ‘negative frequency’ means imaginary frequency, e.g. -173.53 means 173.53*i* cm^-1^.

**Supplementary Table 6** Energies and lowest frequencies.

| **Structure** | **SPE** | **ZPE** | **TCH** | **TCG** | **G** | **Lowest frequency** |
| --- | --- | --- | --- | --- | --- | --- |
| **EY^2-^** | -11438.739667 | 0.208151 | 0.233755 | 0.149234 | -11438.590433 | 22.31 |
| **[EY^2-^]^*^ (S1)** | -11438.634966 | 0.208151 | 0.233755 | 0.149234 | -11438.485730 | 22.31 |
| **[EY^2-^]^*^ (T1)** | -11438.667697 | 0.205410 | 0.231365 | 0.144373 | -11438.523324 | 16.72 |
| **EY^-^** | -11438.559394 | 0.208713 | 0.233676 | 0.150310 | -11438.409084 | 14.06 |
| **1a** | -1280.189811 | 0.399552 | 0.426900 | 0.339141 | -1279.850670 | 10.54 |
| **1a^-^** | -1280.283594 | 0.395999 | 0.423584 | 0.334978 | -1279.948616 | 12.82 |
| **CF_3_C_6_H_4_CO_2_^-^** | -757.366837 | 0.106143 | 0.117759 | 0.067031 | -757.299806 | 12.40 |
| **N_radical** | -522.952275 | 0.286403 | 0.302872 | 0.242267 | -522.710008 | 28.74 |
| **HAT_TS** | -522.939138 | 0.282200 | 0.297624 | 0.241421 | -522.697717 | -1394.15 |
| **C_radical** | -522.973917 | 0.286066 | 0.303064 | 0.240865 | -522.733052 | 20.52 |
| **I_N_radical** | -444.342763 | 0.229755 | 0.243583 | 0.189048 | -444.153715 | 25.28 |
| **I_TS** | -444.321419 | 0.225721 | 0.238161 | 0.188614 | -444.132805 | -1682.06 |
| **I_C_radical** | -444.355864 | 0.229025 | 0.243084 | 0.188961 | -444.166903 | 27.88 |
| **Ⅱ_ N_radical** | -483.647045 | 0.258247 | 0.273427 | 0.215770 | -483.431275 | 28.80 |
| **Ⅱ_TS** | -483.629644 | 0.254093 | 0.268039 | 0.214966 | -483.414678 | -1565.01 |
| **Ⅱ_ C_radical** | -483.665452 | 0.257559 | 0.273095 | 0.214805 | -483.450647 | 24.56 |
| **Ⅳ_ N_radical** | -483.646798 | 0.258668 | 0.273826 | 0.214983 | -483.431815 | 18.95 |
| **Ⅳ_TS** | -483.629637 | 0.254499 | 0.214980 | 0.214980 | -483.414657 | -1389.29 |
| **Ⅳ_ C_radical** | -483.668828 | 0.258165 | 0.273963 | 0.213519 | -483.455309 | 22.46 |

### 6.2 TD-DFT Computational data

**Supplementary Table 7** TD-DFT calculation results for **EY^2-^** using the optimized S0 structure.

| **Structure** | **Excited**  **State** | **Transition**  **Energy/ eV** | **Reported Transition Energy^Ref.^/ eV** | **Absorbing Wavelength /nm** | **Oscillator Strength** |
| --- | --- | --- | --- | --- | --- |
| **EY^2-^** | T1 | 1.8283 | 1.89^8^ | 678.15 | 0 |
|  | S1 | 2.8491 | 3.1^9^ | 435.17 | 0.9657 |
|  | S2 | 3.7241 |  | 332.92 | 0.0178 |
|  | S3 | 4.0648 |  | 305.02 | 0.0868 |
|  | S4 | 4.229 |  | 293.18 | 0.0008 |
|  | S5 | 4.4028 |  | 281.60 | 0.0009 |

## Further transformation of products and gram-scale synthesis

### 7.1 Oxidation to alcohol

The borylation product **2a** was dissolved (59.4 mg, 0.20 mmol) in THF (2.0 mL) and was cooled to 0 °C, then 10% NaOH (1.0 mL) was added, H_2_O_2_ (4.0 equiv., 0.8 mmol, 18.5 μL) was added dropwise. The reaction was stirred at 0 °C for 15 min. Ethyl acetate (20 mL) was added, and the mixture was washed with water (10 mL), brine (10 mL) and dried over Na_2_SO_4_. After removal of the solvent, the residue was purified by column chromatography on silica gel with PE/EtOAc as eluent to give 26.6 mg (white solid, 71% yield) of alcohol.

**N-(tert-butyl)-4-hydroxy-4-methylpentanamide (4)**

**^1^H NMR** (400 MHz, CDCl_3_) δ 5.45 (s, 1H), 3.15 (q, J = 7.0 Hz, 1H), 2.27 (t, J = 7.1 Hz, 2H), 1.79 (t, J = 7.2 Hz, 2H), 1.34 (s, 9H), 1.23 (s, 6H).

**^13^C NMR** (101 MHz, CDCl_3_) δ 173.4, 69.9, 51.2, 38.4, 32.4, 29.4, 28.7.

**HRMS** (ESI) (m/z): Calcd. C_10_H_22_NO^+^ [M+H]^+^: 188.1645. Found: 188.1644.

**IR** (ν/ cm^−1^, neat): 3271, 2966, 2928, 1649, 1556, 1491, 1454, 1417, 1360, 1324, 1294, 1255, 1221, 1162, 1133, 936, 823, 799, 698, 672, 639, 606.

### 7.2 Vinylation of boronic ester

The reaction condition was according to literature^10^. To a solution of borylation product **2a** (59.4 mg, 0.20 mmol) in THF (2.0 mL) was added vinylmagnesium bromide (1.0 M solution in THF, 4.0 equiv., 0.8 mL). The mixture was stirred as room temperature for 1 h. To the above solution was cooled to -78 °C and iodine (4.0 equiv., 0.8 mmol, 203 mg) in methanol (1.0 mL) was added. The reaction mixture was stirred at the -78 °C for 1 h followed by addition of a solution of NaOMe (8.0 equiv., 1.6 mmol, 86.4 mg) in methanol (2.0 mL). After warming to room temperature, the resultant mixture was stirred for another 1.5 h. It was then diluted with pentane (20 mL) and washed with a solution of Na_2_S_2_O_3_ (3.0 mL). Ethyl acetate (20 mL) was added, and the mixture was washed with water (10 mL), brine (10 mL) and dried over Na_2_SO_4_. After removal of the solvent, the reaction mixture was concentrated. Purification by silica gel chromatography gave 17.4 mg (44% yield) as a white solid.

**N-(tert-butyl)-4,4-dimethylhex-5-enamide (5)**

**^1^H NMR** (400 MHz, CDCl_3_) δ 5.73 (dd, J = 17.4, 10.8 Hz, 1H), 5.20 (s, 1H), 4.99 – 4.85 (m, 2H), 2.03 – 1.96 (m, 2H), 1.63 (dd, J = 8.3, 2.8 Hz, 2H), 1.33 (s, 9H), 0.99 (s, 6H).

**^13^C NMR** (101 MHz, CDCl_3_) δ 172.7, 147.6, 111.1, 51.0, 37.8, 36.3, 33.2, 28.8, 26.6.

**HRMS** (ESI) (m/z): Calcd. C_12_H_24_NO^+^ [M+H]^+^: 198.1852. Found: 198.1847.

**IR** (ν/ cm^−1^, neat): 2958, 2924, 2854, 1728, 1659, 1459, 1380, 1283, 1124, 1073, 1039, 799, 742, 704.

### 7.3 Transformation to potassium trifluoroborate salts

In a 25 mL reaction tube with a magnetic stir bar, the borylation product **2a** (59.4 mg, 0.20 mmol) was dissolved in MeCN (2.0 mL). Water (2.0 mL) and KHF_2_ (0.80 mmol, 4.0 equiv., 62.5 mg) were added. The reaction mixture was stirred at room temperature for 12 h, concentrated, and azeotroped with MeOH (5 mL×3). The resulting material was then placed on high vacuum overnight. The crude product was extracted with hot acetone, filtered and then concentrated. Et_2_O (10 mL) was added to the crude, and the mixture waited overnight to precipitate a solid, after filtration, the trifluoroborate salt was obtained as a white solid (35.0 mg, 63% yield).

**potassium (5-(tert-butylamino)-2-methyl-5-oxopentan-2-yl)trifluoroborate (6)**

**^1^H NMR** (400 MHz, Acetone-*d6*) δ 6.49 (s, 1H), 2.12 – 2.06 (m, 2H), 1.46 – 1.33 (m, 2H), 1.30 (s, 9H), 0.67 (s, 6H).

**^13^C NMR** (101 MHz, Acetone-*d6*) δ 175.1, 49.8, 37.5, 34.0, 28.2, 24.6. The signal of the α-B-carbon was not observed.

**^19^F NMR** (471 MHz, Acetone-*d6*) δ -150.11.

**HRMS** (ESI) (m/z): Calcd. C_10_H_20_BF_3_NO^-^ [M-K]^‑^: 238.1596. Found: 238.1594.

**IR** (ν/ cm^−1^, neat): 3634, 3441, 3373, 3255, 3076, 2960, 2928, 2863, 1672, 1632, 1563, 1512, 1452, 1363, 1313, 1261, 1219, 1023, 934, 662, 470.

### 7.4 Gram-scale synthesis

In a glovebox under a nitrogen atmosphere, sequentially added B_2_cat_2_ (25 mmol, 2.5 equiv.,5.95 g), substrate **1a** (10 mmol, 1.0 equiv., 3.59 g), Eosin Y (5%, 0.5 mmol, 346 mg) and 80 mL DMA to a 200 mL thick-walled pressure bottle with a stir bar, followed by DIPEA (20 mmol, 2.0 equiv., 3.5 mL). The capped bottle was removed from the glovebox, and the reaction mixture was irradiated by 467nm Kessil 40W blue LED for 48 hours with a fan cooling. As the catechol boronate esters are sensitive to hydrolysis, RBcat was transformed to RBpin for isolation. After cooling to room temperature, pinacol (4.7 g, 4.0 equiv.) and 30 mL triethylamine were added, and the mixture was stirred at room temperature for 1 hour. 500 mL water was added to the reaction mixture, the aqueous layer was extracted with EtOAc (300ml x 4). If phase separation was slow, brine was added. The organic phases were combined and washed with 500 mL of saturated brine, the organic phase was dried over anhydrous sodium sulfate and filtered, then concentrated under reduced pressure, purified by column chromatography to give the product, 1.59 g, 59% yield.


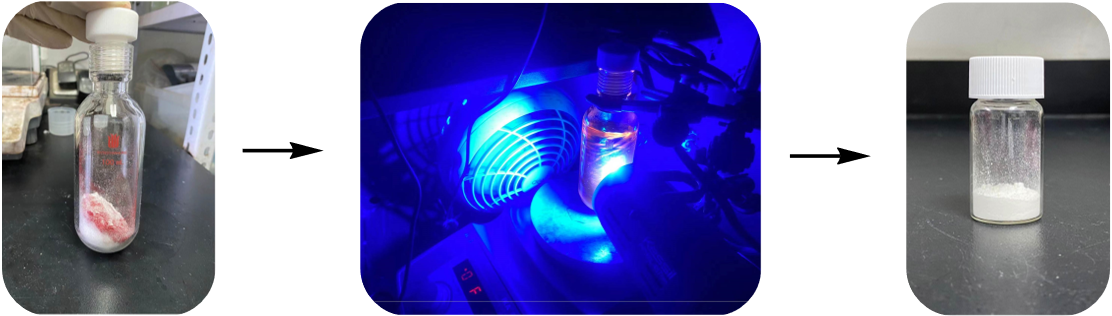


**Supplementary Figure 8** Gram-scale reaction setup. *Left:* the reaction mixture*; middle: the tube* irradiated by blue LED, with a fan cooling; *right:* the product **2a** as a white solid.

# Supplementary References

1. Chen, H.; Guo, L.; Yu, S., Primary, Secondary, and Tertiary gamma-C(sp(3))-H Vinylation of Amides via Organic Photoredox-Catalyzed Hydrogen Atom Transfer. *Org Lett* **2018,** *20* (19), 6255-6259.

2. Ruan, X.-Y.; Zhang, T.; Li, W.-A.; Yin, Y.-Z.; Han, Z.-Y.; Gong, L.-Z., Photoinduced and palladium-catalyzed hydrogen atom transfer triggered 1,2-difunctionalization of 1,3-dienes with hydroxamides. *Science China Chemistry* **2022,** *65* (5), 863-869.

3. Chen, H.; Jin, W.; Yu, S., Enantioselective Remote C(sp3)–H Cyanation via Dual Photoredox and Copper Catalysis. *Organic Letters* **2020,** *22* (15), 5910-5914.

4. Jin, W.; Yu, S., Photoinduced and Palladium-Catalyzed Remote Desaturation of Amide Derivatives. *Organic Letters* **2021,** *23* (17), 6931-6935.

5. Lesimple, P.; BIGG, D. H., Aluminum chloride mediated aminolysis of lactones: a general method for the preparation of ω-hydroxyalkylamides. *Synthesis (Stuttgart)* **1991,** (4), 306-308.

6. Janusz, J. M.; Young, P. A.; Ridgeway, J. M.; Scherz, M. W.; Enzweiler, K.; Wu, L. I.; Gan, L.; Darolia, R.; Matthews, R. S.; Hennes, D.; Kellstein, D. E.; Green, S. A.; Tulich, J. L.; Rosario-Jansen, T.; Magrisso, I. J.; Wehmeyer, K. R.; Kuhlenbeck, D. L.; Eichhold, T. H.; Dobson, R. L. M.; Sirko, S. P.; Farmer, R. W., New Cyclooxygenase-2/5-Lipoxygenase Inhibitors. 1. 7-tert-Butyl-2,3-dihydro-3,3-dimethylbenzofuran Derivatives as Gastrointestinal Safe Antiinflammatory and Analgesic Agents:  Discovery and Variation of the 5-Keto Substituent. *Journal of Medicinal Chemistry* **1998,** *41* (7), 1112-1123.

7. Williams, P. J. H.; Boustead, G. A.; Heard, D. E.; Seakins, P. W.; Rickard, A. R.; Chechik, V., New Approach to the Detection of Short-Lived Radical Intermediates. *Journal of the American Chemical Society* **2022,** *144* (35), 15969-15976.

8. Mau, A. W.-H.; Johansen, O.; Sasse, W. H. F., XANTHENE DYES AS SENSITIZERS FOR THE PHOTOREDUCTION OF WATER. *Photochemistry and Photobiology* **1985,** *41* (5), 503-509.

9. Daly, S.; Kulesza, A.; Knight, G.; MacAleese, L.; Antoine, R.; Dugourd, P., The Gas-Phase Photophysics of Eosin Y and its Maleimide Conjugate. *The Journal of Physical Chemistry A* **2016,** *120* (20), 3484-3490.

10. Scott, H. K.; Aggarwal, V. K., Highly Enantioselective Synthesis of Tertiary Boronic Esters and their Stereospecific Conversion to other Functional Groups and Quaternary Stereocentres. *Chemistry - A European Journal* **2011,** *17* (47), 13124-13132.
