# Supplementary material for: Transition metal-free visible light photoredox-catalyzed remote C(sp3)−H borylation enabled by 1,5-hydrogen atom transfer
Source: Commun Chem. 2023 Jul 24;6:156. doi: 10.1038/s42004-023-00960-z (PMC10366130; doi:10.1038/s42004-023-00960-z)
Supplement: Supplementary file 2 — Description of Additional Supplementary Files [file 42004_2023_960_MOESM2_ESM.pdf]

Description of Additional Supplementary Files

File name: Supplementary Data 1

Description: Cartesian coordinates of the structures

File name: Supplementary Data 2

Description: NMR spectra
