# Supplementary material for: Transition metal-free visible light photoredox-catalyzed remote C(sp3)−H borylation enabled by 1,5-hydrogen atom transfer
Source: Commun Chem. 2023 Jul 24;6:156. doi: 10.1038/s42004-023-00960-z (PMC10366130; doi:10.1038/s42004-023-00960-z)
Supplement: Supplementary file 3 — Supplementary Data 1 [file 42004_2023_960_MOESM3_ESM.docx]

**Cartesian coordinates of the structures**

**EY^2-^**

C -3.63920000 0.29336000 -0.15478800

C -3.67622000 -1.16998600 -0.08572300

C -2.34919800 -1.75608100 -0.00670400

C -1.18927100 -1.00614700 -0.00319800

C -1.21582700 0.41810200 -0.08576900

C -2.49627500 1.03686800 -0.15334100

O -0.00025800 -1.67236500 0.06319100

C 1.18889100 -1.00638600 -0.00322800

C 1.21574100 0.41784200 -0.08576000

C 0.00002700 1.13823900 -0.08606100

C 2.34866600 -1.75655200 -0.00677200

C 3.67581800 -1.17078500 -0.08590700

C 3.63906100 0.29261400 -0.15477700

C 2.49628800 1.03636300 -0.15332100

O 4.73206600 -1.83374600 -0.10179400

Br 5.33362700 1.18076500 -0.26408600

Br -2.24980800 -3.65357200 0.08041900

O -4.73258000 -1.83274600 -0.10174200

Br -5.33358300 1.18185900 -0.26391000

C 0.00022100 2.60768600 -0.39006200

C 0.00098900 3.60862600 0.59832500

C 0.00167700 4.95331200 0.19944300

C 0.00151000 5.31424300 -1.14772200

C 0.00045300 4.31717300 -2.12998100

C -0.00022100 2.97487400 -1.74826800

C 0.00071700 3.25347200 2.10349300

O 0.00128000 2.01725500 2.34425400

O -0.00002900 4.22419600 2.90186600

H -2.54745700 2.11856500 -0.19362200

H 2.54768100 2.11803900 -0.19358700

H 0.00218200 5.69551700 0.99300300

H 0.00211100 6.36531700 -1.43463100

H 0.00018500 4.57956900 -3.18660800

H -0.00098800 2.19501400 -2.50712800

Br 2.24888900 -3.65401800 0.08063700

**[EY^2-^]* (S1)**

C -3.63920000 0.29336000 -0.15478800

C -3.67622000 -1.16998600 -0.08572300

C -2.34919800 -1.75608100 -0.00670400

C -1.18927100 -1.00614700 -0.00319800

C -1.21582700 0.41810200 -0.08576900

C -2.49627500 1.03686800 -0.15334100

O -0.00025800 -1.67236500 0.06319100

C 1.18889100 -1.00638600 -0.00322800

C 1.21574100 0.41784200 -0.08576000

C 0.00002700 1.13823900 -0.08606100

C 2.34866600 -1.75655200 -0.00677200

C 3.67581800 -1.17078500 -0.08590700

C 3.63906100 0.29261400 -0.15477700

C 2.49628800 1.03636300 -0.15332100

O 4.73206600 -1.83374600 -0.10179400

Br 5.33362700 1.18076500 -0.26408600

Br -2.24980800 -3.65357200 0.08041900

O -4.73258000 -1.83274600 -0.10174200

Br -5.33358300 1.18185900 -0.26391000

C 0.00022100 2.60768600 -0.39006200

C 0.00098900 3.60862600 0.59832500

C 0.00167700 4.95331200 0.19944300

C 0.00151000 5.31424300 -1.14772200

C 0.00045300 4.31717300 -2.12998100

C -0.00022100 2.97487400 -1.74826800

C 0.00071700 3.25347200 2.10349300

O 0.00128000 2.01725500 2.34425400

O -0.00002900 4.22419600 2.90186600

H -2.54745700 2.11856500 -0.19362200

H 2.54768100 2.11803900 -0.19358700

H 0.00218200 5.69551700 0.99300300

H 0.00211100 6.36531700 -1.43463100

H 0.00018500 4.57956900 -3.18660800

H -0.00098800 2.19501400 -2.50712800

Br 2.24888900 -3.65401800 0.08063700

**[EY^2-^]* (T1)**

C -3.69821600 0.43620500 -0.12353400

C -3.79023700 -1.00686700 -0.04559100

C -2.47826900 -1.64180800 0.01502500

C -1.29119700 -0.94225000 -0.01956200

C -1.25480100 0.48347600 -0.11930000

C -2.51559800 1.14120800 -0.15295500

O -0.12898200 -1.67251800 0.04812700

C 1.09063800 -1.05196000 -0.02999300

C 1.19337400 0.37046100 -0.11184200

C -0.00205100 1.16369300 -0.15424600

C 2.20930000 -1.85801300 -0.04170600

C 3.56902500 -1.33733500 -0.14743600

C 3.61452800 0.10873000 -0.24203700

C 2.50268600 0.91427400 -0.21797200

O 4.58491500 -2.07706300 -0.16527300

Br 5.33984600 0.91014000 -0.40816000

Br -2.44778800 -3.53667100 0.13806900

O -4.87100000 -1.64908900 -0.02910200

Br -5.35017700 1.40287200 -0.18530300

C 0.06175100 2.63578500 -0.37150500

C 0.45608200 3.57365700 0.61373800

C 0.49436600 4.93620300 0.27369000

C 0.16005700 5.38985200 -1.00147400

C -0.24636400 4.46824300 -1.97341300

C -0.29675400 3.11283700 -1.65082500

C 0.82368900 3.19034300 2.06728700

O 1.06304300 1.97056800 2.28063200

O 0.85528400 4.13710500 2.89598400

H -2.53404500 2.22346500 -0.19312300

H 2.61001900 1.98970100 -0.26925000

H 0.78194500 5.62532000 1.06276400

H 0.20615600 6.45311100 -1.23555200

H -0.52038800 4.79988900 -2.97416100

H -0.61163300 2.39116700 -2.40220100

Br 2.01325700 -3.73848700 0.06598000

**EY^-^**

C -3.64122700 0.33324600 -0.17490400

C -3.65730200 -1.12966800 -0.13531200

C -2.33397600 -1.72165400 0.01269900

C -1.18145700 -0.95978000 0.11867600

C -1.23001700 0.45096600 0.08213800

C -2.49187200 1.07052800 -0.07528900

O -0.00000800 -1.63486300 0.24831100

C 1.18140700 -0.95982000 0.11861900

C 1.23003900 0.45090100 0.08225800

C 0.00001600 1.25488100 0.35485600

C 2.33397200 -1.72172700 0.01266100

C 3.65719200 -1.12973700 -0.13534200

C 3.64128600 0.33311400 -0.17467100

C 2.49193400 1.07043500 -0.07499600

O 4.70129900 -1.80195900 -0.23021500

Br 5.31248600 1.21262400 -0.37805800

Br -2.22850000 -3.60423300 0.05140900

O -4.70136100 -1.80182500 -0.23004400

Br -5.31247400 1.21275700 -0.37821400

C 0.00005600 2.63700700 -0.27341500

C 0.00012400 3.61620200 0.71198900

C 0.00012400 4.97626900 0.40069400

C 0.00012100 5.33510700 -0.94842500

C 0.00004800 4.34765400 -1.94841900

C -0.00001200 2.98766000 -1.62239600

C 0.00009000 2.95891300 2.04533800

O 0.00004600 1.61907200 1.85353000

O 0.00014300 3.48482900 3.13995200

H -2.54918500 2.15314100 -0.09713400

H 2.54927400 2.15304800 -0.09669900

H 0.00012800 5.72008000 1.19255200

H 0.00012200 6.38472100 -1.23142500

H -0.00000800 4.64616100 -2.99392900

H -0.00009700 2.22723900 -2.39856200

Br 2.22836100 -3.60427400 0.05093100

**1a**

C -2.50242900 -0.80710700 -1.07178200

H -1.82123700 -1.40940200 -0.46687000

H -2.50821000 -1.20927600 -2.09091100

C -3.93356500 -0.87407000 -0.49057600

H -3.92501500 -0.50568900 0.54403300

H -4.56877200 -0.19223100 -1.07093300

C -4.55725700 -2.28429600 -0.52494800

H -4.51945700 -2.63970000 -1.56700600

C -3.79497700 -3.29187400 0.35052800

H -4.27976900 -4.27540100 0.32238100

H -2.75898200 -3.43099700 0.02043600

H -3.77086700 -2.96141400 1.39759100

C -6.03501400 -2.21540300 -0.10682500

H -6.60094200 -1.52635200 -0.74592500

H -6.51035300 -3.20170100 -0.17322000

H -6.13374400 -1.86844400 0.93062800

C -2.03428700 0.63464600 -1.20750600

O -2.29182900 1.29014600 -2.20854200

N -1.26607000 1.19114000 -0.17245200

C -1.22193500 2.69091400 0.06686900

C -0.49224100 3.34686300 -1.12151300

H -1.05126100 3.24120400 -2.05077400

H -0.36244600 4.41381300 -0.90543700

H 0.50133200 2.90462300 -1.25536300

C -2.64944800 3.24374900 0.22897700

H -2.60709600 4.31941900 0.43548600

H -3.23763100 3.09543700 -0.68040800

H -3.16031200 2.75880600 1.06868000

C -0.41048800 2.96658600 1.34394600

H 0.60633200 2.56634800 1.27134500

H -0.32917300 4.05149200 1.46737900

H -0.89112200 2.56516900 2.24018700

O -1.44473800 0.45845800 1.04561800

C -0.35155800 -0.14822900 1.63285200

O -0.54219700 -0.59221400 2.74112300

C 0.98502700 -0.24757200 0.95631000

C 1.19190600 -0.48080700 -0.41024700

C 2.09618200 -0.20006400 1.81755700

C 2.48526900 -0.65330100 -0.90766600

H 0.36037100 -0.54330000 -1.09797100

C 3.38616400 -0.34727900 1.32039300

H 1.93330800 -0.04947500 2.87958700

C 3.58131800 -0.57517900 -0.04735600

H 2.63381000 -0.84163700 -1.96536600

H 4.23729200 -0.29299500 1.99239000

C 4.98610400 -0.76664400 -0.56119500

F 5.04794500 -0.79205400 -1.91241700

F 5.52539400 -1.93243500 -0.11665300

F 5.81376400 0.22415600 -0.14040600

**1a^-^**

C -2.30254200 -0.86928300 -1.12638300

H -1.47035700 -1.29969900 -0.56634700

H -2.25199400 -1.22598800 -2.16222700

C -3.64928600 -1.29006700 -0.50361200

H -3.67893700 -0.95598400 0.54171100

H -4.45527500 -0.76561300 -1.03629700

C -3.92605900 -2.80633000 -0.56065800

H -3.85058800 -3.12172400 -1.61437700

C -2.90964500 -3.62250300 0.25424200

H -3.15296800 -4.69314900 0.22251400

H -1.88834000 -3.50412800 -0.12310600

H -2.90873100 -3.30654600 1.30571400

C -5.35834700 -3.10273900 -0.08578800

H -6.09978900 -2.55664000 -0.68355900

H -5.58883600 -4.17407400 -0.15826300

H -5.49009500 -2.80402800 0.96332200

C -2.17589000 0.64692700 -1.20936400

O -2.59746300 1.26332100 -2.19432400

N -1.54862300 1.28166900 -0.15895200

C -1.61418100 2.76782700 0.06039300

C -0.97261700 3.48572500 -1.14372800

H -1.53183200 3.30970900 -2.06324700

H -0.94715300 4.56419100 -0.94043800

H 0.05842800 3.14253500 -1.28514300

C -3.08226000 3.19961000 0.24999500

H -3.13338600 4.27723000 0.45308500

H -3.67177100 2.98826600 -0.64708200

H -3.52438000 2.66982800 1.10179000

C -0.80542800 3.12008600 1.32055700

H 0.23631500 2.79829500 1.22735100

H -0.81968000 4.20994500 1.44294200

H -1.22725900 2.66338200 2.21915800

O -1.57291900 0.52462700 1.03730800

C -0.34156000 0.07318000 1.67984600

O -0.57138200 -0.20427200 2.86449300

C 0.91192200 -0.06257800 1.00456400

C 1.22574400 0.14312700 -0.38719500

C 2.01620100 -0.48564400 1.83654400

C 2.49914400 -0.06199400 -0.88171100

H 0.46171400 0.46448200 -1.07967700

C 3.28075200 -0.68513600 1.33523400

H 1.81529600 -0.65336200 2.88950000

C 3.56250700 -0.49013000 -0.04684200

H 2.68284300 0.10077000 -1.94065400

H 4.07635500 -1.00571500 2.00419300

C 4.93101300 -0.64289700 -0.55813500

F 4.99543200 -0.81326500 -1.91419900

F 5.77125000 0.44260300 -0.30880600

F 5.61088200 -1.70335400 -0.00430200

**CF_3_C_6_H_4_CO_2_^-^**

C 0.31562600 -1.21966900 -0.02757500

C -1.07997400 -1.20696500 -0.01305900

C -1.79442700 -0.00120400 -0.00513800

C -1.07328300 1.20176200 -0.01323800

C 0.32126900 1.20641600 -0.02824100

C 1.02231400 -0.00892900 -0.03887100

H 0.85603700 -2.16251500 -0.03658100

H -1.65287400 -2.12969800 -0.00958900

H -1.64163900 2.12728900 -0.00977000

H 0.86728400 2.14679400 -0.03748200

C 2.51649000 -0.00249500 -0.00088900

C -3.34884600 0.00295300 0.00904300

O -3.87964600 1.14355300 0.01401500

O -3.88592100 -1.13466000 0.01407300

F 3.06572500 1.00862800 -0.73635300

F 3.07214800 -1.15718000 -0.46547900

F 3.01998500 0.16141700 1.26589000

**N_radical**

N -1.53990500 -0.58747600 0.39549600

C -2.82373700 -0.15598800 -0.15515900

C -2.69888700 0.83563400 -1.32838200

H -2.12659400 0.39335300 -2.15336600

H -3.69459800 1.08704600 -1.71158200

H -2.20772600 1.76171800 -1.01793700

C -3.55164000 -1.43824800 -0.60841000

H -3.63066700 -2.14939700 0.22013800

H -4.56012800 -1.19222500 -0.96049100

H -3.01243100 -1.92604600 -1.42853000

C -3.61014300 0.49155400 1.01829700

H -4.62165000 0.73719100 0.67194600

H -3.68722200 -0.20345200 1.86053100

H -3.11286200 1.40364400 1.35633800

C -0.42084700 0.22337100 0.43250900

O -0.48356800 1.38097100 0.84900100

C 0.87901900 -0.47486600 0.06709000

H 1.01016700 -1.29601700 0.78347600

H 0.73992200 -0.96139700 -0.91074400

C 2.09382800 0.45870800 0.04539300

H 2.23328400 0.89327800 1.04424100

H 1.87677200 1.30442900 -0.62015800

C 3.40345600 -0.21690000 -0.41048200

H 3.22201900 -0.67567800 -1.39606500

C 4.51006100 0.83620400 -0.58197800

H 4.73161100 1.32967500 0.37395300

H 5.43973300 0.38007800 -0.94426000

H 4.21532500 1.61374700 -1.29759700

C 3.86303900 -1.32652700 0.54964700

H 4.03031300 -0.92378800 1.55812200

H 3.13105800 -2.13847800 0.63093800

H 4.80664700 -1.77076000 0.20941400

**HAT_TS**

C 1.95501200 -0.47405400 -0.00198700

C 1.15343700 1.94028700 -0.09413500

C 2.17655400 0.91556300 -0.60891400

H 1.19914100 2.86931200 -0.67264200

H 1.37208900 2.22644000 0.94357500

H 2.08994200 0.83935400 -1.70010200

H 3.19890200 1.26565400 -0.39613800

H 0.70780900 -0.48328800 -0.12539900

N -0.51183200 0.15163700 -0.36940200

C -1.72125100 -0.61031400 -0.00424500

C -2.86612400 -0.16813200 -0.94666000

H -3.75629100 -0.77857500 -0.74863100

H -3.11060100 0.88426500 -0.78841000

H -2.57575200 -0.30833900 -1.99371800

C -1.40922600 -2.09688900 -0.25580800

H -2.30042500 -2.70450600 -0.06346700

H -1.09686900 -2.26048600 -1.29327500

H -0.61074400 -2.45383800 0.40619100

C -2.11865000 -0.39563200 1.46918200

H -3.01138400 -0.98588900 1.70932400

H -1.31201900 -0.71786000 2.13936000

H -2.33734900 0.65741300 1.66489600

C -0.30816700 1.47608300 -0.10732400

O -1.22526200 2.28525800 0.07238100

C 2.49259600 -1.62001700 -0.84308700

H 3.59153800 -1.56558600 -0.90139000

H 2.23583700 -2.59512600 -0.41266400

H 2.10597400 -1.58268800 -1.86791300

C 2.28563300 -0.59387700 1.47904600

H 1.97000700 -1.56351700 1.88188400

H 3.37379000 -0.51497200 1.62968700

H 1.81244600 0.19060000 2.07918700

**C_radical**

C -2.79551500 0.21902400 -0.05791800

C -2.94054600 0.40425300 1.46460200

H -2.54325800 1.37835500 1.77405600

H -3.99900200 0.35970300 1.74861800

H -2.40151400 -0.37930800 2.00245300

C -3.52566800 1.35440600 -0.79385200

H -3.42732000 1.25116600 -1.88234100

H -4.59339300 1.33329800 -0.55225100

H -3.13477400 2.33667500 -0.49827800

C -3.36845800 -1.14222300 -0.49681500

H -4.43093400 -1.20491900 -0.23220800

H -3.27828600 -1.26725400 -1.58260600

H -2.83751000 -1.96144400 -0.00604100

C -0.35116000 -0.49318300 -0.03582300

O -0.50943300 -1.42927300 0.74847000

C 1.02624100 -0.16831300 -0.61298900

H 1.31863400 -1.01803300 -1.24254500

H 0.99871700 0.71765200 -1.26004900

C 2.08307800 0.02858700 0.50458700

H 2.01695200 -0.84844400 1.16675100

H 1.79466000 0.89961100 1.10856300

C 3.48113100 0.20173700 -0.02167000

C 4.24131100 1.46110500 0.26202200

H 4.75382600 1.42386300 1.24345400

H 5.02470300 1.64208700 -0.48653000

H 3.58499700 2.34063600 0.28680700

C 4.24243200 -1.00789600 -0.47414900

H 4.81529400 -1.46227300 0.35723500

H 3.58242400 -1.79368700 -0.86205000

H 4.97413100 -0.76334900 -1.25641300

N -1.36744000 0.32110900 -0.44895300

H -1.12787300 1.05712100 -1.09967900

**I_N_radical**

N 0.68325300 -0.30632100 -0.66371400

C 1.96219600 -0.19410800 0.03647800

C 1.82097200 -0.01685500 1.56097600

H 1.28463400 -0.86544400 2.00367700

H 2.81273300 0.02837500 2.02556500

H 1.28587500 0.90425600 1.80738000

C 2.74813100 -1.48096200 -0.28676200

H 2.83991800 -1.61412800 -1.36954400

H 3.75256800 -1.42678400 0.14867900

H 2.24345500 -2.36224500 0.12527700

C 2.69929900 1.02553800 -0.58379700

H 3.70953800 1.07886700 -0.15948500

H 2.78080400 0.91465200 -1.66982500

H 2.16402000 1.95169600 -0.36253800

C -0.45750500 0.36501500 -0.26283800

O -0.43576600 1.57127400 -0.01497700

C -1.72904800 -0.46711700 -0.29273000

H -1.83335400 -0.87593800 -1.30760000

H -1.57458600 -1.34253500 0.35719500

C -2.98690100 0.30230500 0.12208000

H -3.11611600 1.16325400 -0.54417300

H -2.84392900 0.71860300 1.12698600

C -4.24068600 -0.57968600 0.09386700

H -4.14607800 -1.43318900 0.77783000

H -4.42653900 -0.97957300 -0.91136600

H -5.12833200 -0.01059600 0.39411300

**I_TS**

C 1.87193000 -1.66015900 0.26199300

C 2.22399400 0.81317600 0.09910600

C 2.72759700 -0.56739100 -0.35240400

H 1.92411200 -1.73988500 1.35176700

H 1.88831500 -2.63505400 -0.23074800

H 2.71696300 1.62500200 -0.44587100

H 2.45923200 0.97211100 1.16176500

H 2.67745300 -0.63970300 -1.44599900

H 3.78518100 -0.68948700 -0.07470500

H 0.72789900 -1.13706800 -0.00385500

N -0.03103700 -0.08680800 -0.30297500

C -1.47133200 -0.24418700 -0.01581400

C -2.25756100 0.62815700 -1.02298800

H -3.33291800 0.45972300 -0.88455300

H -2.03783500 1.68681300 -0.87114900

H -1.99364200 0.35816900 -2.05136200

C -1.81743800 -1.72514400 -0.25497900

H -2.89264900 -1.88396900 -0.11848300

H -1.55030600 -2.02938300 -1.27333300

H -1.28903600 -2.37730900 0.45119300

C -1.81488300 0.15504800 1.43182700

H -2.88673300 0.01547200 1.61775500

H -1.26479800 -0.46875000 2.14732800

H -1.56594100 1.20352800 1.61568100

C 0.71103800 1.03154700 -0.03492100

O 0.22648700 2.16264500 0.06305800

**I_C_radical**

C 1.94095100 -0.14682600 0.05733200

C 2.09176900 0.61871500 1.38609000

H 1.77882500 -0.00805500 2.22976000

H 3.14063700 0.90018400 1.53935600

H 1.48414600 1.52682500 1.38195800

C 2.77053200 -1.44033600 0.10385200

H 2.67586000 -2.00762200 -0.83115500

H 3.82996700 -1.20213000 0.24405800

H 2.45926500 -2.08348400 0.93719800

C 2.39661200 0.72538600 -1.12798500

H 3.44725900 1.01251200 -1.00009700

H 2.30475400 0.17363600 -2.07122800

H 1.79252500 1.63343300 -1.19440700

C -0.55397600 0.25936100 -0.23814700

O -0.48249600 1.48612600 -0.16262100

C -1.89038000 -0.44464900 -0.47990400

H -2.15912500 -0.27069800 -1.53136000

H -1.80531800 -1.53091300 -0.34829600

C -3.00659500 0.11359000 0.42408300

H -2.99270800 1.21185200 0.31656400

H -2.76160600 -0.08126400 1.47752900

C -4.35319700 -0.43769400 0.09843300

N 0.52907500 -0.56495600 -0.13065600

H 0.35715800 -1.55879200 -0.20558100

H -4.65420600 -0.58913700 -0.93541600

H -5.11529100 -0.54594700 0.86415200

**Ⅱ_ N_radical**

N 1.23415100 0.32423900 0.66478800

C 2.48775500 0.17359000 -0.07284400

C 2.29536100 -0.03366800 -1.58798900

H 1.75804100 0.81368800 -2.03184100

H 3.27119200 -0.10402000 -2.08219000

H 1.73887300 -0.95120300 -1.79752000

C 3.30565700 1.45281100 0.19705400

H 3.43477600 1.60720100 1.27318600

H 4.29439000 1.37167900 -0.26916600

H 2.80301100 2.33403600 -0.21760900

C 3.22313900 -1.04546500 0.55053600

H 4.21868500 -1.12565300 0.09672100

H 3.34031000 -0.91323700 1.63090700

H 2.66574200 -1.96665400 0.36604300

C 0.06839100 -0.33213300 0.31446000

O 0.05903000 -1.54371600 0.09356800

C -1.18476300 0.52655200 0.36435400

H -1.24171100 0.97027100 1.36811600

H -1.03712500 1.37656400 -0.31991500

C -2.47153600 -0.23012400 0.02222000

H -2.59468000 -1.06581500 0.72262500

H -2.37501700 -0.68427700 -0.97279900

C -3.71307600 0.67169800 0.06244000

H -3.58323700 1.50521400 -0.64363700

C -5.00733400 -0.08059000 -0.26976200

H -5.18227300 -0.89894500 0.44010500

H -5.87738600 0.58588900 -0.23216900

H -4.96472800 -0.51812500 -1.27536700

H -3.80172200 1.12741400 1.05963500

**Ⅱ_TS**

C 2.06170100 -0.73091600 0.43696800

C 1.55305300 1.73661300 0.32173600

C 2.45873400 0.59963200 -0.18880200

H 2.29790000 -0.77918700 1.50771800

H 1.77991500 2.68523700 -0.17179500

H 1.70532100 1.87973800 1.40084100

H 2.36949000 0.52994100 -1.28209500

H 3.51350800 0.82968700 0.02520200

H 0.79952600 -0.56895300 0.49584600

N -0.33059000 0.19499100 0.47888200

C -1.55369800 -0.49581200 0.02290900

C -1.67938200 -0.49979000 -1.51275400

H -2.58940000 -1.03112800 -1.81733900

H -1.72855200 0.52062700 -1.90203600

H -0.82276900 -1.01007100 -1.97053500

C -1.47816800 -1.93938900 0.55286300

H -2.39955300 -2.47814700 0.30565800

H -0.63999400 -2.48602800 0.10436000

H -1.35413300 -1.94824000 1.64162300

C -2.76911300 0.21265900 0.66647900

H -3.68511800 -0.33510300 0.41150700

H -2.66802800 0.23016400 1.75720200

H -2.85415100 1.23969000 0.30568600

C 0.06989700 1.44044400 0.07814800

O -0.69828200 2.29306300 -0.37908800

C 2.42994600 -1.99366100 -0.31155700

H 3.52475100 -2.09422100 -0.37882900

H 2.05090100 -2.89155700 0.18935200

H 2.04295700 -1.98056700 -1.33777500

**Ⅱ_ C_radical**

C -2.45071800 0.19197600 0.10876800

C -2.51212200 -0.35298700 1.54840600

H -2.11274200 0.38245300 2.25696600

H -3.55187900 -0.56611300 1.82467500

H -1.93089700 -1.27383000 1.63714000

C -3.23949900 1.50819500 0.01373800

H -3.20734500 1.91960500 -1.00356200

H -4.28996000 1.33649800 0.27059100

H -2.84403400 2.26067100 0.70829200

C -3.02463200 -0.83507600 -0.88599600

H -4.07125100 -1.05122200 -0.63976600

H -2.98749200 -0.44338900 -1.90960600

H -2.45769600 -1.76838500 -0.84869500

C 0.00146800 -0.34975000 -0.30848100

O -0.09560400 -1.54666200 -0.03633400

C 1.33137200 0.25795100 -0.75612400

H 1.51854200 -0.10012200 -1.77822500

H 1.27858900 1.35317200 -0.80710500

C 2.49802200 -0.17294300 0.15000900

H 2.46190400 -1.27330200 0.23811100

H 2.33054600 0.20402400 1.17128700

C 3.83230000 0.28317500 -0.34619800

C 5.02736200 0.30535500 0.54982900

H 5.39791000 -0.71290600 0.77286800

H 5.86283000 0.85656700 0.10282700

H 4.79624300 0.76546200 1.52150300

N -1.04872700 0.52138200 -0.25037000

H -0.85824100 1.48414000 -0.49442100

H 3.98956400 0.33492700 -1.42332900

**Ⅳ_ N_radical**

N 1.81957800 0.14197400 0.53273100

C 3.02424200 0.10447000 -0.27567900

C 3.50704400 1.55567100 -0.47322600

H 3.71287400 2.02964100 0.49263700

H 4.42758600 1.55434100 -1.06816400

H 2.75907600 2.15662500 -1.00185400

C 4.08551000 -0.73929400 0.45532100

H 5.01293400 -0.74953900 -0.13030600

H 4.29702300 -0.31982700 1.44519900

H 3.74055300 -1.77042600 0.57745200

C 0.67824800 -0.48461600 0.05919800

O 0.75733500 -1.54974900 -0.55685300

C -0.62985600 0.16533500 0.47145900

H -0.68343600 0.11307000 1.56904200

H -0.55547900 1.23626700 0.23764200

C -1.86671700 -0.47598800 -0.16533400

H -1.83736800 -1.55437800 0.03114600

H -1.81374200 -0.36857900 -1.25858000

C -3.20788300 0.09611200 0.33710400

C -4.37809700 -0.73772100 -0.20878700

H -4.28611400 -1.79254400 0.07776600

H -5.33927600 -0.36862100 0.17003800

H -4.41308000 -0.69226500 -1.30575800

H -3.21916900 0.01010400 1.43565900

H 2.81801300 -0.35142000 -1.25399000

C -3.39196000 1.57978900 -0.02245500

H -2.61750400 2.21566000 0.42218100

H -3.36000500 1.72289100 -1.11156500

H -4.36179800 1.95062700 0.33156500

**Ⅳ_TS**

C 1.87123100 -0.44867500 0.04867100

C 0.81652700 1.87225000 -0.05847400

C 1.86982600 0.91521100 -0.65442600

H 0.75383500 2.79949800 -0.63430100

H 1.08648800 2.14553400 0.97039000

H 1.65037600 0.77267500 -1.72093100

H 2.86906800 1.37387300 -0.59778400

H 0.64772300 -0.52733800 0.31941000

N -0.63073300 -0.01267300 0.48223500

C -1.71395200 -0.97108700 0.23479600

C -2.07723500 -1.13904100 -1.24871100

H -2.82940600 -1.92916700 -1.36351600

H -2.48406600 -0.21056500 -1.65929700

H -1.19733200 -1.42520200 -1.83764400

C -2.93756700 -0.60791400 1.09928500

H -3.69102300 -1.40151700 1.02030800

H -2.64992800 -0.50600400 2.15103300

H -3.37659000 0.33514800 0.76335300

C -0.58252200 1.25133300 -0.03833000

O -1.58651800 1.87679300 -0.39486000

C 2.25138300 -1.62198800 -0.83929500

H 3.30227700 -1.53325000 -1.15831900

H 2.15033400 -2.57866400 -0.31293100

H 1.63727300 -1.66055400 -1.74650000

H -1.32631000 -1.92925700 0.60764400

C 2.55703500 -0.45970000 1.40820900

H 2.39373600 -1.40884200 1.93193100

H 3.64391100 -0.33191400 1.28417900

H 2.19854400 0.34758000 2.05586700

**Ⅳ_ C_radical**

C 3.05581100 0.04722300 0.03057400

C 3.36581700 -1.23612900 0.81520500

H 3.30235800 -2.11900800 0.16550600

H 4.37815100 -1.19614100 1.23586200

H 2.65695500 -1.35994300 1.64005400

C 4.03014200 0.26995700 -1.13044200

H 3.81650200 1.21008800 -1.65123700

H 5.06113200 0.30853200 -0.76154800

H 3.97315500 -0.54973300 -1.86054200

C 0.61591100 0.53703700 0.22109500

O 0.73772800 1.05050200 1.33324000

C -0.74074800 0.43157900 -0.46987400

H -1.05281200 1.45329400 -0.72075400

H -0.67080800 -0.12305800 -1.41441300

C -1.80466000 -0.22005400 0.44903100

H -1.77332700 0.32017500 1.40837200

H -1.49732100 -1.25257100 0.66283500

C -3.19126200 -0.20305700 -0.13348200

C -3.94602400 -1.48557300 -0.30674900

H -4.45927700 -1.79306600 0.62567500

H -4.72792600 -1.39842200 -1.07331600

H -3.28562700 -2.31629300 -0.58712000

N 1.68017800 0.04078900 -0.47802900

H 1.50468700 -0.41229600 -1.36566200

H 3.10805300 0.89612700 0.71928100

C -3.96155300 1.08341300 -0.13610100

H -4.53884700 1.21228900 0.79985200

H -3.30854500 1.96078700 -0.22083800

H -4.69017500 1.12331800 -0.95728000
