# Supplementary material for: Transition metal-free visible light photoredox-catalyzed remote C(sp3)−H borylation enabled by 1,5-hydrogen atom transfer
Source: Commun Chem. 2023 Jul 24;6:156. doi: 10.1038/s42004-023-00960-z (PMC10366130; doi:10.1038/s42004-023-00960-z)
Supplement: Supplementary file 4 — Supplementary Data 2 [file 42004_2023_960_MOESM4_ESM.docx]

**NMR Spectra**

**Spectra of hydroxamic acid derivatives**

**Spectra of products**

**Figure 1** 1D NOESY experiment with selective excitation of **2h** ^1^H NMR spectrum (in blue) in **H1** (in green) and **H2** (in red). No NOE signals between them were detected.

**Figure 2** 1D NOESY experiment with selective excitation of **2i** ^1^H NMR spectrum (in blue) in **H1** (in green) and **H2** (in red). No NOE signals between them were detected.

**Figure 3** 1D NOESY experiment with selective excitation of **2j** ^1^H NMR spectrum (in purple) in **H1** (in blue) , **H2** (in green) and **H3** (in red). No NOE signals between them were detected.


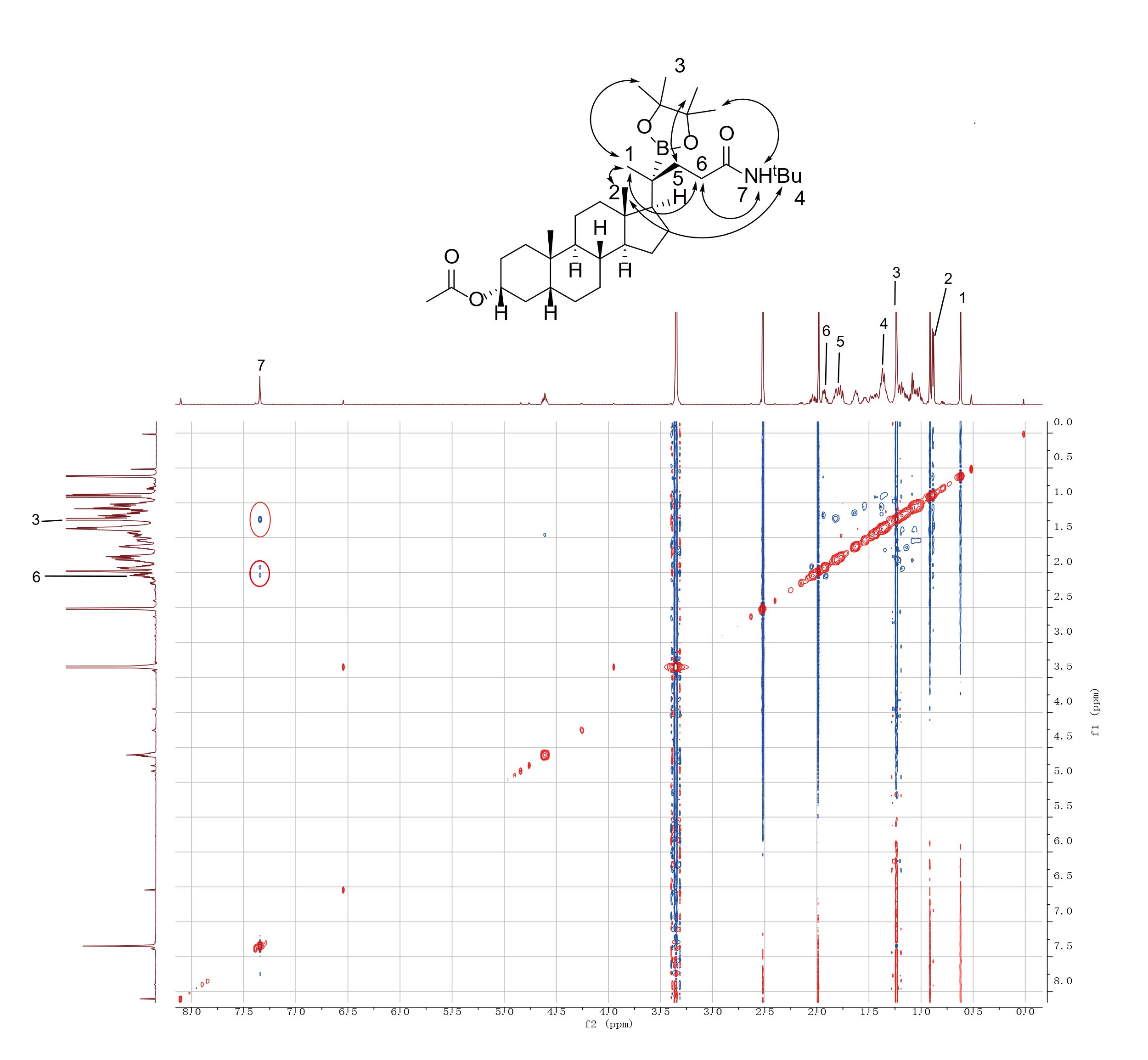

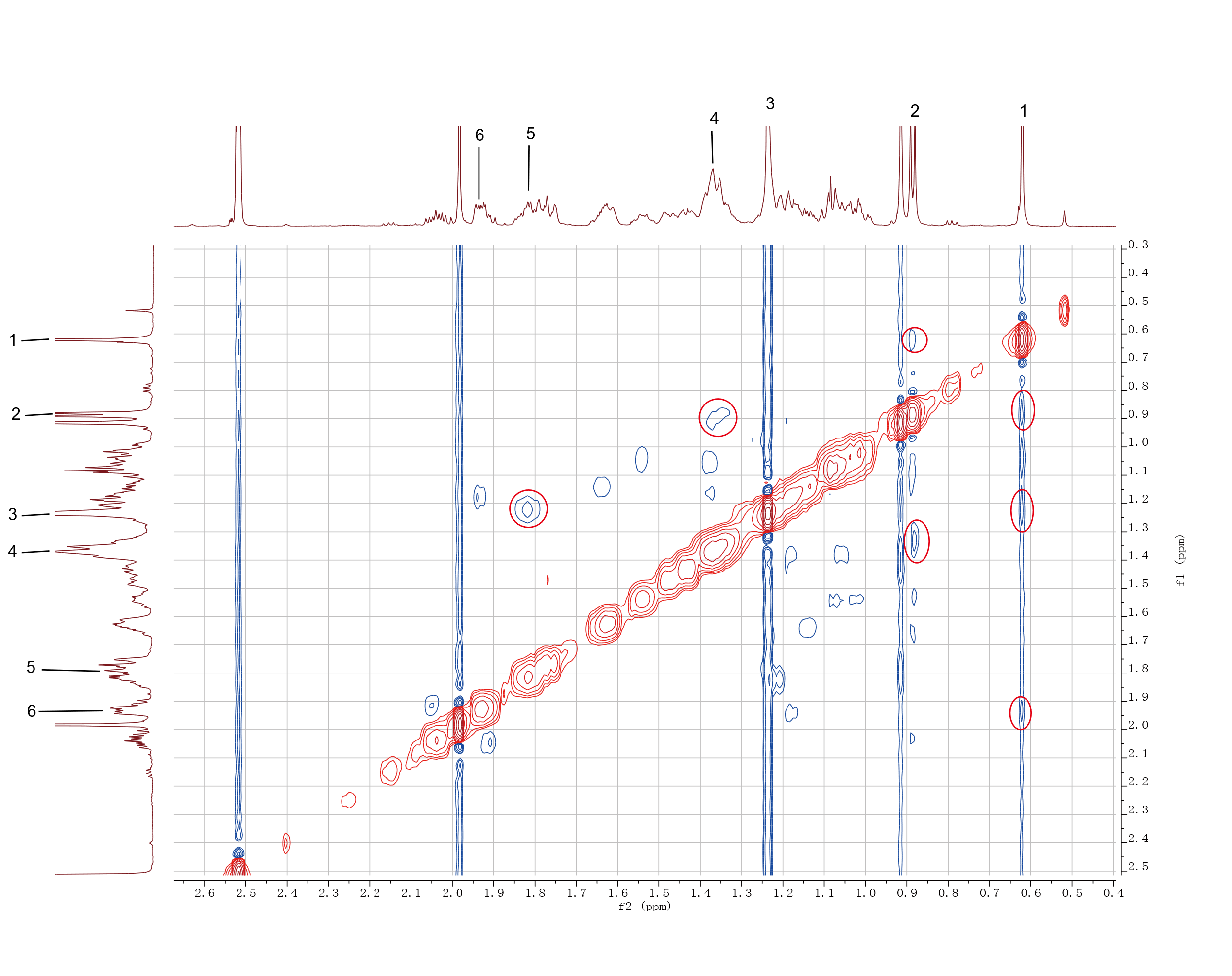


**Figure 4** 2D ^1^H-^1^H NOESY NMR spectra of **2aa** in DMSO-*d6* and certain NOE interactions observed (marked by red circle).
